# Decoy-PROTAC for specific degradation of “Undruggable” STAT3 transcription factor

**DOI:** 10.1038/s41419-025-07535-x

**Published:** 2025-03-21

**Authors:** Shiqing Li, Xin Wang, Jiabao Huang, Xiuping Cao, Yana Liu, Shiyan Bai, Tao Zeng, Qi Chen, Chunsen Li, Chunhua Lu, Huanghao Yang

**Affiliations:** 1 New Cornerstone Science Laboratory, MOE Key Laboratory for Analytical Science of Food Safety and Biology, College of Chemistry, Fuzhou University, Fuzhou, People’s Republic of China; 2 State Key Laboratory of Structural Chemistry, Fujian Institute of Research on the Structure of Matter, Chinese Academy of Sciences, Fuzhou, Fujian People’s Republic of China; 3 Interdisciplinary Institute for Medical Engineering, Fuzhou University, Fuzhou, People’s Republic of China

**Keywords:** Proteolysis, Cancer therapy

## Abstract

Signal transducer and activator of transcription 3 (STAT3) is widely recognized as an attractive target for cancer therapy due to its significant role in the initiation and progression of tumorigenesis. However, existing STAT3 inhibitors have suffered from drawbacks including poor efficacy, limited specificity, and undesirable off-target effects, due to the challenging nature of identifying active sites or allosteric regulatory pockets on STAT3 amenable to small-molecule inhibition. In response to these obstacles, we utilize the innovative proteolysis targeting chimera (PROTAC) technology to create a highly specific decoy-targeted protein degradation system for STAT3 protein, termed D-PROTAC. This system fuses DNA decoy that targets STAT3 with an E3 ligase ligand, utilizing a click chemistry approach. Experimental results demonstrate that D-PROTAC efficiently mediates the degradation of the STAT3 protein across various cancer cell types, leading to the downregulation of crucial downstream STAT3 targets, inhibiting tumor cell growth, triggering cell cycle arrest and apoptosis, and suppressing tumor immune evasion. Furthermore, D-PROTAC is capable of achieving significant tumor suppression in xenograft models. Overall, our research validates that D-PROTAC can successfully target and eliminate the “undruggable” STAT3, showcasing specificity and potent antitumor effects. This strategy will suggest a promising avenue for the development of targeted therapies against the critical functions of STAT3 in human cancers and potentially other diseases.

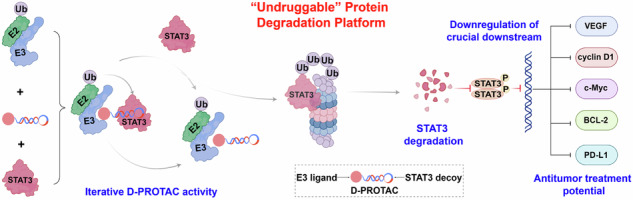

## Introduction

Transcription factors (TFs), as key regulators of gene expression, play a critical role in determining cellular function [1]. Generally, misregulation of TFs may lead to inappropriate gene expression patterns that drive oncogenesis [2, 3]. One prominent example of such a TF is signal transducer and activator of transcription 3 (STAT3), which plays a role in cell proliferation, survival, differentiation, and angiogenesis [4]. STAT3 is activated primarily through direct phosphorylation of tyrosine residues induced by its upstream ligands, including Janus kinase (JAK), tyrosine kinases, cytokines, and non-receptor tyrosine kinases [5, 6]. The activated STAT3 monomer forms a dimer structure via the Src Homology 2 (SH2) domain and is transferred to the nucleus by karyopherin, where it binds to specific DNA sequences, functioning in gene transcription regulation [7, 8]. Importantly, STAT3 can be activated in a wide range of human tumors, including solid and hematological tumors, and its overexpression has been observed in various patient-derived tumor tissue samples [9]. Compelling evidence suggests that suppressing expression or blocking activation of STAT3 significantly inhibits tumor progression, and most normal adult tissues are spared from the effects of STAT3 deletion [10]. Therefore, STAT3 has been recognized as an attractive potential therapeutic target in cancer.

However, the development of STAT3 inhibitors for clinical application has been challenging and slow due to the lack of active sites or allosteric regulatory pockets, which has led to STAT3 being considered as an “undruggable” target [11]. Currently, the most common approach for attenuating STAT3 signaling has been predominantly focused on inhibiting its upstream kinases [12, 13]. For instance, inhibitors targeting upstream JAK kinases like ruxolitinib can reduce STAT3 phosphorylation and activity [14]. Alternatively, using antisense oligonucleotides that bind to STAT3 mRNA, blocking its translation [15, 16]. Although targeting upstream of STAT3 can successfully reduce its activity, the associated risk of off-target toxicity remains a significant concern. Directly targeting STAT3 protein enables more specific anti-cancer activity while minimizing off-target reactions. The main methods for direct STAT3 inhibition include disrupting its SH2 domains [17], DNA binding domains (DBD) [18], or N-terminal domains (NTD) [19]. Generally, direct inhibitors of STAT3 are divided into three categories: peptides [20], small molecules [21], and oligonucleotides [22]. For instance, the peptide C16 derived from the STAT3-binding sites of SH2 domain containing adapter protein F (SHF), disrupts dimer formation and inhibits STAT3 activity [17]. Although some of these STAT3 inhibitors have shown practicality in preclinical studies, low water solubility and limited selectivity issues have prevented their clinical approval. Oligonucleotides represent a new therapeutic strategy for “undruggable” cancer targets. For example, the DNA decoy that binds to the DBD of STAT3 can improve the selectivity for STAT3 [23], allowing for further clinical development.

**Scheme 1:**
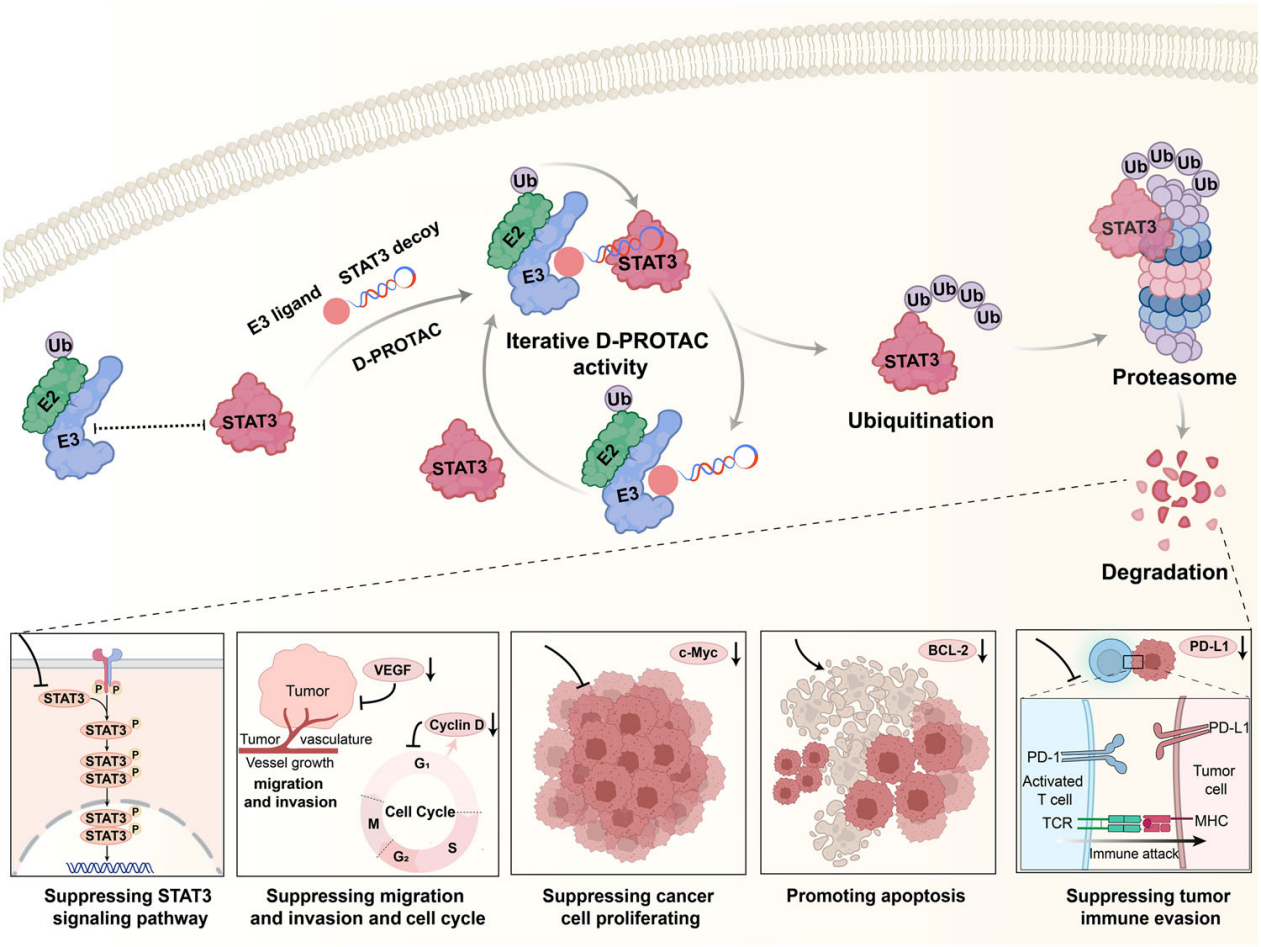
Schematic diagram of the D-PROTAC strategy. D-PROTAC can recruit the VHL E3 ligase to ubiquitinate the STAT3, which is subsequently degraded by the proteasome. D-PROTAC efficiently mediates the degradation of the STAT3 protein in cancer cells, leading to the downregulation of crucial downstream STAT3 targets, inhibiting tumor cell growth, triggering cell cycle arrest and apoptosis, and suppressing tumor immune evasion.

Proteolysis targeting chimera (PROTAC), an emerging technology, shows promise in inducing targeted protein degradation [24]. It utilizes a heterobifunctional molecule to simultaneously recruit the protein of interest (POI) and E3 ligases. By linking ligands that bind to each of them, PROTAC facilitates the degradation of specific proteins through the ubiquitin-proteasome system. Compared to traditional inhibitors, PROTAC not only degrades but also functionally inhibits POI, providing stronger efficacy, selectivity, and potential for clinical applications [25, 26]. In this study, we utilized a decoy strategy to construct a decoy-targeted chimera for specific and efficient degradation of STAT3 protein (Scheme 1). Specifically, we employed a DNA decoy as a ligand to target STAT3 by binding to its DBD, termed STAT3-D. The STAT3-D was then attached to a small molecule ligand of the E3 ligases Von Hippe-Lindau (VHL-L) through a click chemistry reaction, generating bifunctional biomolecules termed as decoy-targeted protein degradation chimeras (D-PROTAC). Upon entry into the cell, D-PROTAC selectively bound to STAT3 via the DNA decoy. At the same time, D-PROTAC could recruit the VHL to form a ternary complex known as STAT3-D-PROTAC-VHL. Within this complex, the E3 ligases mediated the ubiquitination of STAT3 by the E2 ubiquitin-conjugating enzyme. The ubiquitinated STAT3 was recognized and degraded by the proteasome. This strategy allows for selective targeted degradation of STAT3 protein, based on the selective recognition and binding of the DNA decoy to STAT3. D-PROTAC mediates the efficient and highly selective degradation of STAT3 protein. This leads to the downregulation of key downstream targets of STAT3, inhibiting tumor cell growth. Also, D-PROTAC induces cell cycle arrest and apoptosis, and suppresses tumor immune evasion, thereby demonstrating the selectivity and antitumor potential. In vivo studies further assessed the therapeutic potential of D-PROTAC in xenograft mouse models. The experimental results validate the potential of using decoy oligonucleotides to construct PROTACs, overcoming the “undruggable” nature of STAT3. Therefore, the presented strategy introduces an innovative approach for regulating “undruggable” targets in tumor cells, providing a therapeutic strategy for treatment of STAT3-regulated cancers.

## Materials and methods

### Chemicals and materials

DNA Oligonucleotides were synthesized by Sangon Biotech. VHL Ligand-Linker Conjugates 8 (T17909) and MG132 (T2154) were purchased from TargetMol Chemicals. Phytohemagglutinin (PHA, MB2559) and phorbol myristate acetate (PMA, MB5349) were from Meilunbio. Fixative solution (4% formaldehyde, methanol-free, BL539A) was from Biosharp. Crystal violet ammonium oxalate solution (0.4%, G1070) was obtained from Solarbio. Antibodies for STAT1 (ab234400, 1:1000), STAT2 (ab32367, 1:5000), STAT4 (ab284408, 1:1000), STAT5a + STAT5b (ab194898, 1:1000), STAT6 (ab32520, 1:2000), BCL-2 (ab182858, 1:1000), c-Myc (ab32072, 1:1000), and VEGF (ab32152, 1:5000) were all obtained from Abcam. Antibodies for STAT3 (4904, 1:2000), p-STAT3 (Y705) (9145, 1:2,000), PD-L1(13684, 1:1000), cyclin D1 (55506, 1:1000), β-actin (4970, 1:1000), and Anti-rabbit IgG (7074, 1:2000) were from Cell Signaling Technology. The ELISA kit for IL-2 (EK102) was obtained from MultiSciences. Annexin V-FITC apoptosis assay kit (C1062L), cell cycle (C1052) and Calcein/PI cell viability/cytotoxicity assay kit (C2015M) were from Beyotime. Cell Counting Kit-8 (HY-K0301) was obtained from MedChemExpress.

### Synthesis of D-PROTAC

#### Preparation of D-PROTAC

We synthesized D-PROTACs with different linker lengths. The 3’ ends of the DNA decoy were extended by 7, 11, 15, 19, 23, and 27 bases, respectively, (Table S1). An appropriate amount of PBS was taken to dilute these six DNA decoy with different linker lengths to 10 μM, and then were denatured at 95 °C in a metal bath for 5 min. These samples were then rapidly inserted into ice for renaturation and left to stand at room temperature for 1 h. They were mixed in a ratio of DNA: VHL Ligand-Linker Conjugates 8 = 1:100, and shaken on a 37 °C shaker for 12 h.

##### Purification of D-PROTAC

The solution that had been shaken for 12 h was added to an ultrafiltration centrifuge tube with a target molecular weight of 3 K and centrifuged at 7500 rpm for 25 min in a centrifuge. After centrifugation, ultrapure water was added to a total volume of 400 μL, and it was centrifuged again in a centrifuge at 7500 rpm for 25 min. After centrifugation, the above steps were repeated once more. D7-PROTAC, D11-PROTAC, D15-PROTAC, D19-PROTAC, D23-PROTAC and D27-PROTAC were prepared. They were sealed and stored in the dark at −20 °C.

### Characterization of D-PROTAC

#### Mass spectrometry

After being prepared, D-PROTACs with six different linker lengths (D7-PROTAC, D11-PROTAC, D15-PROTAC, D19-PROTAC, D23-PROTAC, D27-PROTAC) were concentrated to 20 μM by ultrafiltration and sent for analysis to Sangon Biotech.

##### Polyacrylamide gel electrophoresis (PAGE)

PAGE was performed to investigate the synthesis of the products. All of the products including STAT3-D were mixed with 6×DNA loading buffer. Electrophoresis was performed with 12% polyacrylamide gel at 100 V in 1×Tris-borate-EDTA (TBE, pH 8.0) for 60 min. After staining with Gel-Red, the gel was recorded by gel imaging analysis system.

### Cell culture

HeLa, MCF-7, Jurkat, Caco-2, MCF-10A, and LO2 cell lines were purchased from American Type Culture Collection (cat#CCL-2 for HeLa, cat#HTB-22 for MCF-7, cat#TIB-152 for Jurkat, cat#HTB-37 for Caco-2, cat#CRL-10317 for MCF-10A, and cat#CRL-2706 for LO2). MCF-7, Jurkat and LO2 cells were cultured in RPMI-1640 medium (16600082, Thermo Fisher Scientific) with 10% fetal bovine serum (FBS, Gibco) and 1% penicillin–streptomycin (PS, MA0110, Meilunbio). HeLa cell was cultured in MEM medium (11875101, Thermo Fisher Scientific) with 10% FBS, 1% PS and 1% sodium pyruvate (11360070, Gibco). Caco-2 cell was cultured in DMEM medium (11875101, Thermo Fisher Scientific) with 20% FBS, and 1% PS. MCF-10A cell was cultured in cell special medium (CM-0525, Procell). Both cell lines were grown at 37 °C under 5% CO_2_/95% air in a humidified incubator.

### Western blot (WB)

HeLa, MCF-7 or Caco-2 lines were seeded (2 × 10^5^ cells/well) on 6-well transparent plates and subjected to starvation treatment for 24 h. The test compounds were added following starvation, and cells were incubated for additional some hours, then washed with cold-ice PBS for 2 times and 60 μL of ice-cold lysis buffer containing 1% protease and phosphatase inhibitors (K1015, Apexbio). Cells were scraped off after 5 min on ice and centrifuged for 12,000 rpm for 15 min at 4 °C to obtain the protein lysate. The protein extract was denatured at 100 °C bath and analyzed on 10% SDS-PAGE gels. The gels were blotted onto PVDF membrane (1610374, Bio-Rad) and blocked with 5% BSA Buffer (5% Albumin Bovine V from Bovine serum in TBST) for 2 h at room temperature. BSA (V900933) was from Sigma-Aldrich and TBST (9997) was from Cell Signaling Technology. Then, corresponding specific primary antibodies were added and incubated overnight at 4 °C. After the completion of primary antibody incubation, the PVDF membrane was washed three times with 1×TBST buffer solution and secondary antibodies conjugated with horseradish peroxidase (HRP) were added, followed by incubation at room temperature for 1.5 h. Subsequently, the PVDF membrane was washed three times with 1×TBST to remove excess secondary antibodies. Finally, ECL high-sensitivity chemiluminescent substrate was added, and protein imaging and band analysis were performed on the PVDF membrane using a Bio-Rad ChemDoc imaging system.

### Modeling of the STAT3-D11-PROTAC-VHL complex and STAT3-D11-PROTAC-VHL-ElonginC-ElonginB complex

The following complexes were analyzed: Transcription factor STAT3B/DNA complex (PDB ID: 1BG1), VHL (PDB ID: 1ZA8) and VHL-ElonginC-ElonginB complex (PDB ID: 1VCB). The original oligonucleotide sequence of STAT3B was replaced by STAT3-D11 (5’-AAACATTTCCCG-TAAATCGAAAGATTTACGGGAAATG-3’-VHL-L). VHL-L has been optimized with Gaussian16 software at DFT(B3LYP-D3) level, employing the def2svp basis set. In order to obtain the optimal conformation of D11-PROTAC, MD simulations have been performed in the NVT (constant number of atoms, volume and temperature) ensemble using an integration time step of 0.5 fs within Amber22 package. Then D11-PROTAC was attached to the STAT3, VHL and complex VHL-ElonginC-ElonginB with molecular docking. To prepare the protein for docking, all waters and ions were removed from the structure except for the conserved catalytic water. The protein receptors and the ligand were pre-processed with AutoDockTools. All protein macromolecules were kept rigid and minimal number of rotatable bonds was allowed for the ligand. Docking simulations were performed with Autodock4 using the Lamarckian genetic algorithm with default parameters and 20 runs. After completing the docking screens, D11-PROTAC was attached to the complex VHL-ElonginC-ElonginB and STAT3.

### Stability testing of D-PROTAC

The D11-PROTAC molecules at a concentration of 0.5 μM were separately added to 1×TAE buffer solution with pH 5.0 and PBS buffer solution containing 10% FBS. These solutions were then incubated at a constant temperature of 37 °C for 0, 6, 12, 24, 48, and 72 h, respectively. At the end of the incubation period, gel electrophoresis imaging was performed using a 12% PAGE gel to observe the band changes and assess the stability of the prepared D11-PROTAC molecules in the acidic buffer solution and 10% FBS.

### Confocal microscopy

Cellular fluorescence images were collected on a Nikon-Eclipse-Ti confocal microscope equipped. HeLa or MCF-7 cells (2.5 × 10^4^) were placed in a glass-bottom confocal dish and incubated overnight at 37 °C. Then, HeLa or MCF-7 cells were treated with 250 nM of cy3-D11-PROTAC, in serum-free cell culture medium. After incubated for 3 h at 37 °C, the cells were washed twice times with PBS solution. Subsequently, the cells were visualized by confocal microscopy.

### Cellular uptake analysis by flow cytometry

MCF-7 and HeLa cells (2 × 10^5^ cells/well) were separately seeded in a 6-well cell culture plate. After 24 h, the cells were subjected to a 24 h starvation treatment using starvation medium. The cells were then co-incubated with Cy3-labeled D11-PROTAC and Cy3-labeled STAT3-D11 at a concentration of 250 nM under 37 °C conditions for 3 h. Subsequently, the cells were digested with trypsin and centrifuged at 1000 rpm for three min, and the supernatant was discarded. The cells were washed and resuspended in PBS, followed by centrifugation. This washing and centrifugation process was repeated twice. Finally, flow cytometry was used to analyze the cells.

### Migration assay

HeLa and MCF-7 cells were, respectively, seeded in a 6-well plate and allowed to adhere until the cells covered the bottom surface of the wells. After starving the cells with corresponding starvation medium for 24 h, a straight scratch was made uniformly across the bottom surface of the 6-well plate using a pipette tip. Then, the cells were gently washed with PBS to remove excess cell debris. The cells were then observed using an inverted fluorescence microscope, and photographs were taken to record the condition of the scratch area at this moment, representing the baseline (0 h) of cell migration. Subsequently, 1.5 μM of STAT3-D11, VHL-L, and D11-PROTAC were added, respectively, to the 6-well plate, and the cells were cultured further in a CO_2_ incubator. At different time points (24 and 48 h), the scratch area of the cells was observed again using an inverted fluorescence microscope, and the cell growth and migration in the scratch area were recorded.

### Transwell invasion assay

Matrigel (082724, Abwbio) was diluted in serum-free culture medium at a ratio of 1:8. The diluted Matrigel (100 μL) was added vertically to the upper chamber of the Transwell (3422, Corning), evenly spread on the bottom, and then placed in an incubator for 3 h to allow Matrigel to polymerize into a gel matrix. The excess liquid in the upper chamber was removed, and 100 μL of serum-free culture medium was added to each well and left in the incubator for 30 min for basement membrane hydration. In the lower chamber of a 24-well plate, 500 μL of culture medium containing 10% FBS was added, and the Transwell chambers with coated Matrigel were placed in the wells. Cells were digested and adjusted to a density of 2.5 × 10^5^/mL. Then, 200 μL of cell suspension was added to each well of the Transwell chambers. Plates were maintained at 37 °C and 5% CO_2_ for 24 h. Subsequently, 1.5 μM of STAT3-D11, VHL-L, and D11-PROTAC were added to each well and incubated for 24 h. Invasion was stopped washing the well three with PBS and using a cotton bud to remove the remaining cell of the top part of the membrane, being careful not to compromise the Matrigel. The membrane was fixed with 10% formalin (30 min at room temperature) and stained with crystal violet for 20 min. Cells were counted under the microscope.

### Cell cycle analysis

For cell cycle analysis, HeLa or MCF-7 cells (3 × 10^5^ cells/well) were seeded into 6-well plates (3516, Corning) and subjected to starvation treatment. Following starvation, the cells were treated with 1.5 μM of D11-PROTAC. The cell culture medium was collected into a centrifuge tube for future use. The cells were digested with trypsin until they could be gently pipetted or blown down by a pipette or pipette tip. The previously collected cell culture medium was added, and all the adherent cells were gently pipetted down, and the cells were gently dispersed. They were then collected again in a centrifuge tube. The cells were centrifuged at approximately 1000 × *g* for 3–5 min to pellet them. The supernatant was removed, and approximately 1 mL of pre-chilled PBS was added to resuspend the cells, which were then transferred to a 1.5 mL centrifuge tube. The cells were centrifuged again to pellet them, and the supernatant was removed. The bottom of the centrifuge tube was gently flicked to appropriately disperse the cells and prevent cell clumping. Approximately 1 mL of ice-cold 70% ethanol was added, and a gentle mixing was performed to homogenize the cells. The cells were fixed at 4 °C for 24 h. After fixation, the cells were centrifuged at approximately 1000 × *g* for 3–5 min to pellet them. The supernatant was removed, and approximately 1 mL of pre-chilled PBS was added to resuspend the cells. The cells were centrifuged again to pellet them, and the supernatant was removed. The bottom of the centrifuge tube was gently flicked to appropriately disperse the cells and prevent cell clumping. Each cell sample tube was then added with 0.5 mL of PI staining solution with PI plus RNase A (PI: RNase A = 1:9), and the cell pellet was slowly and thoroughly resuspended. The cells were incubated at 37 °C in the dark for 30 min. After staining, red fluorescence was detected at an excitation wavelength of 488 nm using a flow cytometry.

### Colony formation assay

HeLa or MCF-7 cell lines were collected, resuspended in medium and seeded into 6-well plates with single‑cell suspension. Subsequently, the cells were counted and their density was adjusted to 1000 cells/well. Subsequently, STAT3-D11 and VHL-L at a concentration of 1.5 μM was added as a control, followed by the addition of D11-PROTAC at the same concentration. All compounds were re-added every 24 h for 12 days until cell aggregation became visibly apparent, at which point the culture was stopped. After 12 days, the culture medium was discarded, and the cells were gently washed three times with PBS buffer. Then, 200 μL of 4% cell tissue fixation solution was added and left at room temperature for 15 min. After 15 min, the cell tissue fixation solution was discarded, and the cells were washed three times with PBS. Subsequently, 200 μL of 0.4% crystal violet staining solution was added and left at room temperature for 15 min. After staining, the cells were washed multiple times with PBS to remove excess crystal violet outside the cell aggregates, and then photographed and analyzed.

### CCK-8 assay

Cultured HeLa, MCF-7, MCF-10A, LO2 cells were seeded into 96-well plates (3599, Corning) at the density of 1 × 10^4^ cells/well in a volume of 100 μL and allowed to adhere and grow at 37 °C for 24 h. After this, the cells are subjected to starvation treatment using starvation medium (MEM medium with 1% PS and 1% sodium pyruvate, RPMI-1640 medium with 1% PS or DMEM medium with 1% PS) for another 24 h. Following starvation, the cells were treated with D-PROTAC at a range of concentrations for 48 h. CCK8 solution (10 μL/well) was added, and the incubation continued for 2 h. Then, the OD values (450 nm) were measured using a microplate reader.

The data normalized to the untreated control and presented as “% Inhibition” using the following formula: (OD_A_ – OD_B_)/(OD_C_ – OD_B_) 100%, which was used to determine IC50 values. OD_A_ represents groups with different concentrations of D11-PROTAC, OD_B_ represents blank groups with only medium, and OD_C_ represents control groups.

### Calcein-AM/PI double staining

To further evaluate cell growth of HeLa and MCF-7, Calcein-AM/PI double staining kit (C2015M, Beyotime) was used according to the manufacturer’s instructions. Cultured HeLa and MCF-7 cells were seeded into 96-well plates at the density of 1 × 10^4^ cells/well in a volume of 100 μL and allowed to adhere and grow at 37 °C for 24 h. After this, the cells are subjected to starvation treatment using starvation medium (MEM medium with 1% PS and 1% sodium pyruvate or RPMI-1640 medium with 1% PS) for another 24 h. Following starvation, the cells were treated with D11-PROTAC at 1.5 μM for 48 h. Then, cells were labeled with 100 μL of Calcein AM/PI detection working solution and were incubated at 37 °C in the dark for 30 min. After incubation, followed by fluorescence microscopy to observe the fluorescence images by detecting red (PI, Ex/Em = 535/617 nm) and green (Calcein-AM, Ex/Em = 495/517 nm) signals on SparkCyto.

### Annexin V apoptosis assay

For cell apoptosis detection, Annexin V-FITC apoptosis assay kit (C1062L, Beyotime) was used. Briefly, HeLa or MCF-7 cells (3 × 10^5^ cells/well) were seeded into 6-well plates and subjected to starvation treatment for 24 h. After starvation, the cells were treated with 1.5 μM of D11-PROTAC. The cell culture medium was drawn into a suitable centrifuge tube. Adherent cells were washed once with PBS, and an appropriate amount of cell digestion solution (without EDTA) containing trypsin was added for cell digestion. The cells were incubated at room temperature until gentle pipetting or blowing could dislodge the adherent cells, after which the trypsin-cell digestion solution was aspirated. The previously collected cell culture medium was added, and the cells were gently pipetted down and transferred to a centrifuge tube. They were then centrifuged at 1000 × *g* for 5 min, and the supernatant was discarded. The cells were collected, gently resuspended in PBS, and counted. From the resuspended cells, 5–10 thousand were taken and centrifuged at 1000 × *g* for 5 min. The supernatant was discarded, and 195 μL of Annexin V-FITC binding solution was gently added to resuspend the cells. Five microliters of Annexin V-FITC was added and mixed gently. Then, 10 μL of PI staining solution was added and mixed gently. The cells were incubated at room temperature (20–25 °C) in the dark for 10–20 min, after which they were placed in an ice bath. Flow cytometry (Beckman CytoFLEX Flow Cytometer) was immediately used to detect the cells, with Annexin V-FITC showing green fluorescence and PI showing red fluorescence.

### Coculture experiments and IL-2 detection

MCF-7 or HeLa cells were seeded into a 12-well plate at a density of 1 × 10^4^ cells/well for attachment. Subsequently, the cells were treated with 1.5 μM of D11-PROTAC for 24 h. After that, Jurkat T cells (10 × 10^4^) were pre-stimulated with PHA (10 μg/mL) and PMA (10 ng/mL) for 4 h. MCF-7 cells or HeLa cells were co-cultured with activated Jurkat T cells for 24 h. The level of IL-2, a biomarker of T cell activation, in the supernatant was measured using an ELISA assay kit. The underlying living cancer cells were virtualized with crystal violet staining.

### In vitro cytotoxic T lymphocyte (CTL) activity detection

Cytotoxic T lymphocyte (CTL) activity was analyzed using the CCK8 method. Briefly, MCF-7 cells or HeLa cells were used as target cells (T) and seeded in a 96-well plate at a density of 1 × 10^4^ cells/well. The plate was placed in a CO_2_ incubator and cultured for 24 h. Subsequently, 1.5 μM of STAT3-D11, VHL-L, and D11-PROTAC were added and the cells were further incubated for 24 h. After drug treatment, Jurkat T cells were added as effector cells (E), and the ratios of effector cells to target cells were 5:1, 10:1, 20:1, and 40:1. Target cells and effector cells were co-incubated at 37 °C for 24 h. The CCK8 solution (10 μL) was added to each well, followed by a 4 h incubation. The cytotoxicity of the target cells was determined, and the CTL activity (%) was calculated using the formula: CTL activity (%) = [ODT − (ODS − ODE)]/ODT × 100%, where ODT is the optical density value of the target cell control, ODE is the optical density value of the effector cell control, and ODS is the optical density value of the samples.

### In vivo fluorescence imaging

All the animal experiments were conducted in agreement with the guide for the care and use of laboratory animals (Ministry of Science and Technology of China, 2006). All animal procedures were approved by the Institutional Animal Care and Use Committee of Fuzhou University. We selected female nude mice to establish the subcutaneous xenograft tumor model of the MCF-7 cell. The MCF-7 tumor-bearing mice were intratumoral injected with D11-PROTAC solution (100 μL, 10 μM). In vivo living imaging of mice was performed, and Cy5 signals were analyzed using the Ami X optical imaging system (Spectral Instruments Imaging Co., USA).

### In vivo antitumor assay

For animal studies, the sample sizes were not determined using formal statistical methods but were based on practical considerations, including the expected effect size, variability, and resource availability. *N* = 5 animals per group was used to ensure reliable and reproducible results. All animal models were allocated randomly to each group. In animal experiments, the main investigators were blinded in the process of group allocation and tail vain administration. In other experiments, the main Investigators were not blinded since the experimental design, execution and data analysis were performed by the same person. MCF-7 tumor-bearing mice were randomly divided into two groups: (a) PBS (control group), (b) D11-PROTAC (100 μL, 10 μM). Each group was intratumoral injected every 2 days for a total of five times. The tumor sizes were measured by digital calipers every 2 days. On the 28th day, photos of living mice and tumors were acquired to evaluate the therapeutic efficacy.

### Histological analysis

MCF-7 tumor-bearing mice were subjected to intratumoral injection of different materials. The mice were weighed by an electronic balance every 2 days. After sacrificing mice, tumors and main organs were cut into slices and stained using hematoxylin and eosin (H&E) and terminal deoxynucleotidyl transferase-mediated dUTP nick-end labeling (TUNEL).

Immunofluorescence Staining: The tumors were sectioned and incubated with the primary antibodies and relevant fluorescein-labeled secondary antibodies according to the manufacturer’s protocol. After staining with DAPI, the acquired slices anchored with different fluorescein were captured by inverted fluorescence microscope.

Immunohistochemistry (IHC): The tumors were sectioned and incubated with the primary antibodies anti-Ki 67 (ab16667, Abcam), and relevant secondary antibodies according to the manufacturer’s protocol. After staining with hematoxylin, photographs obtained were taken with an inverted microscope.

## Results

### Design and synthesis of D-PROTAC

**Fig. 1:**
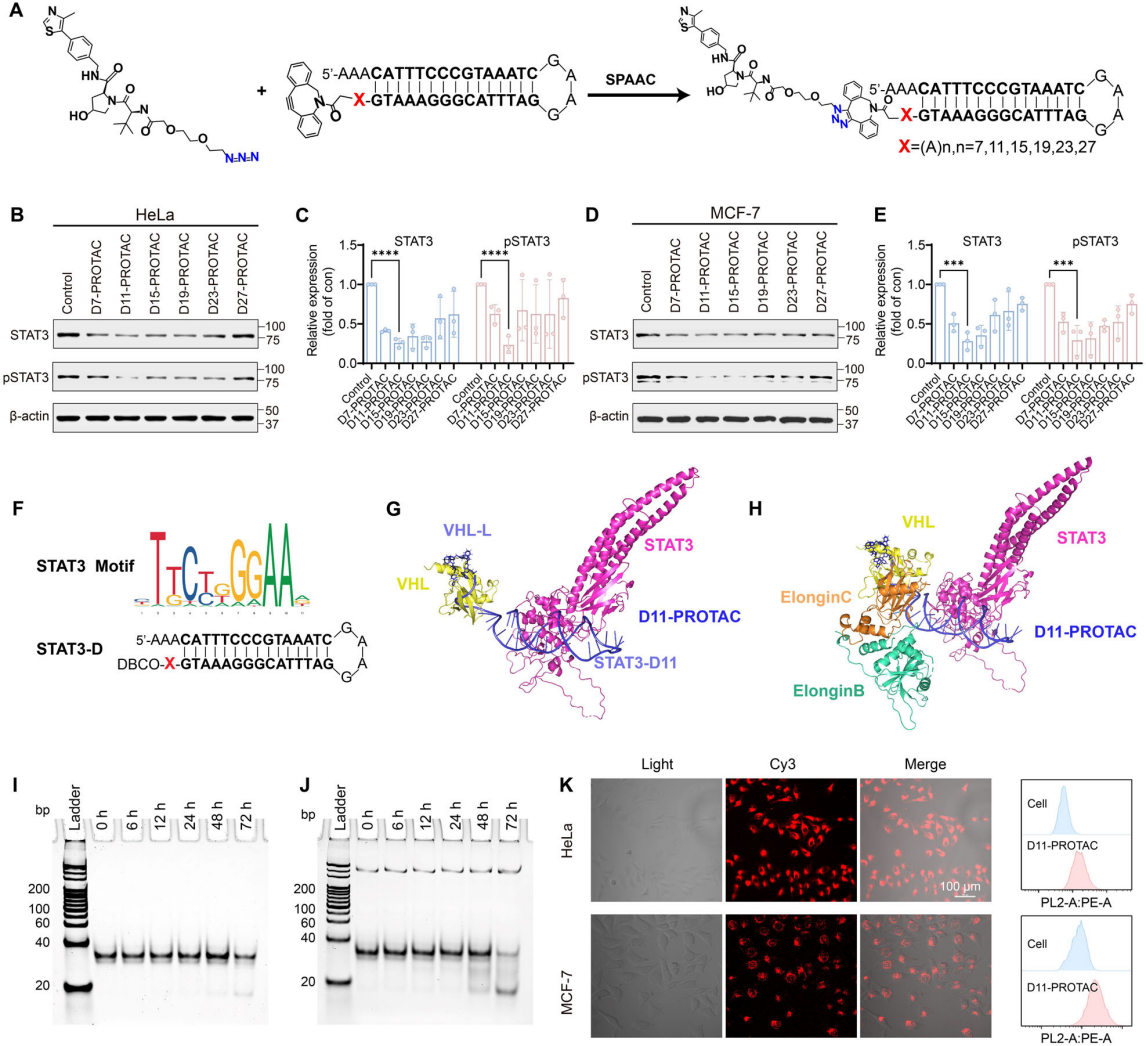
Design and chemical characterization of D-PROTAC as a degrader of STAT3 protein. **A** An azide-functionalized VHL-L was incorporated onto a DBCO-modified DNA decoy (STAT3-D) via a SPAAC reaction, forming a D-PROTAC. The spatial proximity between the STAT3-D and the VHL-L components was tunable by altering the length of the linker. X stands for the A base (adenine). D-PROTAC degraded STAT3 in a linker length-dependent manner. **B** HeLa and **D** MCF-7 cells were treated with the indicated D-PROTAC at a concentration of 1.5 μM for 24 h and analyzed by WB. Quantitative analysis of WB results using ImageJ. STAT3 protein and pSTAT3 protein were analyzed in **C** HeLa and **E** MCF-7 cells. **F** A schematic diagram for the STAT3 motif and STAT3-D. **G** Structural model of the ternary complex of D-PROTAC together with the bound STAT3 (PDB 1BG1 [43]) and VHL (PDB 1ZA8 [44]). **H** Overall structure of STAT3-D-PROTAC-VHL-ElonginC-ElonginB in representation (VHL-ElonginC-ElonginB, PDB 1VCB [45]). Polyacrylamide gel electrophoresis image of D-PROTAC after incubation in **I** pH = 5 buffer and **J** Culture medium supplemented with 10% FBS for 0, 6, 12, 24, 48, and 72 h, respectively. **K** Representative confocal micrographs of HeLa and MCF-7 cells after pretreatment with Cy3-D11-PROTAC. The data are presented as the mean: SD values; *n* = 3. *P < 0.05, **P < 0.01, ***P < 0.001, and ****P < 0.0001 vs. the control group. All controls in this work were the control group without the drug, only the cells under study.

The DNA decoy sequence targeting STAT3 has been demonstrated to effectively target STAT3 in the cytoplasm [23]. In this study, a single-stranded DNA sequence for STAT3 decoy, 5’-AAACATTTCCCG-TAAATCGAAAGATTTACGGGAAATG-3’ (referred to as STAT3-D), was selected for STAT3 targeting. Upon annealing, STAT3-D formed a hairpin structure, exhibiting resistance to nucleases and thermal stability in a cellular environment (Fig. S1). The ligand for VHL, VHL ligand-linker conjugates 8 (VH032-PEG3-N3, referred to as VHL-L), was selected for targeting the E3 ligase [27]. The bifunctional degrader D-PROTAC was constructed utilizing a bioorthogonal copper-free strain-promoted azide−alkyne cycloaddition (SPAAC) reaction [28]. As depicted in Fig. 1A, the SPAAC reaction occurred between an azide-functionalized VHL-L and a dibenzocyclooctyne (DBCO)-modified STAT3-D, yielding the D-PROTAC conjugate. Noteworthy, in D-PROTAC, the spatial proximity between the STAT3-D and the VHL-L components, which is tunable by altering the length of the linker (the ‘X’ in Fig. 1A), plays a pivotal role in modulating the engagement between STAT3 and the E3 ligase.

The interaction between the STAT3 and VHL can be influenced by the length of the D-PROTAC linker. A short linker may restrict the proximity of STAT3 and VHL due to steric hindrance, while an excessively long linker may result in self-inhibition of the D-PROTAC [29, 30]. Therefore, optimizing the linker length can enhance the interaction between STAT3 and VHL, increasing the degradation efficiency of D-PROTAC. To investigate this, six D-PROTACs with different linker lengths were synthesized by incorporating varying numbers of bases (7, 11, 15, 19, 23, 27) at the 3’ end ‘X’ of STAT3-D, designated as STAT3-D7, STAT3-D11, STAT3-D15, STAT3-D19, STAT3-D23, and STAT3-D27. The six D-PROTACs were labeled D7-PROTAC, D11-PROTAC, D15-PROTAC, D19-PROTAC, D23-PROTAC, and D27-PROTAC. Their synthesis was confirmed by mass spectrometry, which showed an increase of 646 Da in molecular weights after conjugation with VHL-L (Fig. S2). Additionally, PAGE confirmed the formation of the six D-PROTACs, as their electrophoretic mobility was slower compared to STAT3-D, providing further evidence of successful construction (Fig. S3).

### Degradation of STAT3 protein by D-PROTAC

STAT3 plays a crucial role in the development of various cancer cells. Therefore, we selected the cervical cancer cell line HeLa and the breast cancer cell line MCF-7 as models to investigate the impact of the six D-PROTACs on STAT3 protein degradation. Western blot (WB) analysis (Figs. 1B–E, S4, and S5) revealed that D-PROTAC with an 11-base linker (D11-PROTAC) demonstrated the most significant degradation of STAT3, including phosphorylated STAT3 (pSTAT3), in both cell lines. This indicated that an 11-base linker optimally positions STAT3 and VHL for effective degradation. Therefore, D11-PROTAC was used in subsequent experiments to further investigate its effects.

### Molecular simulation of D11-PROTAC-mediated STAT3 protein degradation

As mentioned above, D11-PROTAC was the most efficient in degrading STAT3 protein. To validate molecular modeling was used to predict how the ternary complex formed by D11-PROTAC, STAT3, and VHL binds. Within D11-PROTAC, the VHL-L located at the 3’ end of STAT3-D11 could bind to VHL without interference from the DNA part (Fig. 1F, G). STAT3-D11 directly interacted with the STAT3 motif, effectively bringing STAT3 and VHL into closer proximity. This provided an optimal distance for interaction between STAT3 and the E3 ligases, promoting ubiquitination and facilitating STAT3 degradation. VHL forms a complex with ElonginB and ElonginC, known as VBC (VHL-ElonginB-ElonginC), part of the E3 ligase system that mediates protein degradation [31]. To further elucidate the interaction between D11-PROTAC, STAT3, and the E3 ligase system, we further conducted molecular modeling studies on the complex STAT3-D11-PROTAC-VBC (Fig. 1H). The results demonstrate that VHL can bind to both ElonginB and ElonginC to form VBC complex, providing a structural basis for D11-PROTAC recruitment of the E3 ligase system and subsequent ubiquitination of STAT3.

### Stability and cellular uptake of D11-PROTAC

To evaluate the intracellular performance of D11-PROTAC for STAT3 protein degradation, its stability was initially assessed in acidic buffer (pH = 5) and culture medium supplemented with 10% fetal bovine serum (FBS). PAGE analysis (Fig. 1I) demonstrated that D11-PROTAC remained intact even after 72 h of incubation in the acidic buffer, indicating its resistance to acidic conditions. Additionally, D11-PROTAC maintained structural integrity after 48 h in culture medium (Fig. 1J), confirming its stability under physiological conditions for subsequent investigations into STAT3 degradation. Furthermore, the cellular uptake of D11-PROTAC was examined in HeLa and MCF-7 cells using confocal microscopy and flow cytometry with Cy3-labeled D11-PROTAC (Cy3-D11-PROTAC). Confocal microscopy results displayed robust red fluorescence (Cy3 fluorescence) in both HeLa and MCF-7 cells after a 3 h incubation, indicating significant uptake of Cy3-D11-PROTAC in both cell lines (Fig. 1K). Flow cytometry analysis showed that after incubation, HeLa and MCF-7 cells exhibited significantly higher fluorescence intensity in the PE channel compared to control cells (Fig. 1K), confirming the stability and efficient cellular uptake of D11-PROTAC in HeLa and MCF-7 cells.

### D11-PROTAC-induced STAT3 protein degradation and mechanism validation

**Fig. 2:**
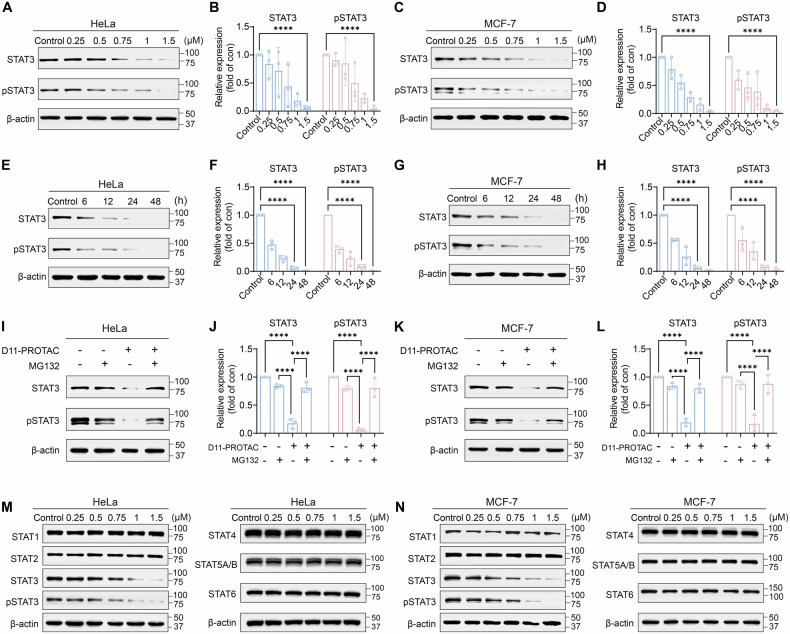
D11-PROTAC potently and selectively degraded STAT3 in multiple cell-based systems. D11-PROTAC degraded STAT3 in dose-dependent and time-dependent manners. **A** HeLa and **C** MCF-7 cells were treated with D11-PROTAC at different concentration (0.25, 0.5, 0.75, 1, and 1.5 µM) for 24 h and analyzed by WB. **E** HeLa and **G** MCF-7 cells were treated with D11-PROTAC at a concentration of 1.5 μM for different times (6, 12, 24, and 48 h) and analyzed by WB. D11-PROTAC mediated STAT3 protein degradation upon co-treatment with proteasome inhibitor MG132. **I** HeLa and **K** MCF-7 cells were pretreated with indicated compounds for 24 h and analyzed by WB. **M**, **N** D11-PROTAC treatment (24 h) reduced STAT3 protein expression, and D11-PROTAC had minimum effects on other STAT family members. **B**, **D**, **F**, **H**, **J**, **L** Quantitative analysis of WB results using ImageJ. STAT3 protein and other STAT family members were analyzed in HeLa and MCF-7 cells. The data are presented as the mean: SD values; *n* = 3. *P < 0.05, **P < 0.01, ***P < 0.001, and ****P < 0.0001 vs. the control group.

Upon confirming the efficient cellular uptake of D11-PROTAC, its ability to degrade STAT3 protein in cancer cells was evaluated using HeLa and MCF-7 cells incubated with varying concentrations of D11-PROTAC. Increased concentrations led to enhanced STAT3 degradation (Figs. 2A, B, and S6). In HeLa cells, degradation started at 0.5 µM and reached 95% at 1.5 µM, with similar results in MCF-7 cells (Figs. 2C, D, and S7). A concentration of 1.5 µM was chosen for a time-dependent study, showing over 90% reduction in STAT3 levels over 24 h in both cell lines (Figs. 2E–H, S8, and S9), confirming D11-PROTAC as a highly effective STAT3 degrader.

To confirm that D11-PROTAC acts through VHL-mediated ubiquitination and proteasomal degradation, the proteasome inhibitor MG132 [32] was used. Pre-treatment of HeLa cells with MG132 followed by D11-PROTAC incubation inhibited STAT3 degradation (Figs. 2I, J, and S10), with similar results in MCF-7 cells (Figs. 2K, L, and S11). This suggests that D11-PROTAC induces STAT3 degradation via a proteasome-dependent pathway. To validate D11-PROTAC-mediated degradation via VHL, a competitive experiment with the VHL ligand VHL-L was conducted. Cells were pretreated with VHL-L and subsequently co-incubated with D11-PROTAC. The results showed that increasing concentrations of VHL-L inhibited STAT3 degradation, suggesting that VHL-L competes with D11-PROTAC for binding to the VHL-E3 ligase, thereby supporting VHL-mediated ubiquitination and proteasomal degradation of STAT3 (Fig. S12). Meanwhile, PROTAC requires binding and recruitment of POI and E3 ligases to form the E3-PROTAC-POI ternary complex, which induces POI ubiquitination and subsequent protein degradation [33]. Incubation of VHL-L, STAT3-D11, and D11-PROTAC in HeLa and MCF-7 cells showed that only D11-PROTAC degraded STAT3 protein (Figs. S13 and S14). This demonstrates that D11-PROTAC can recruit both STAT3 and VHL to form the ternary complex, leading to STAT3 degradation. In summary, these results highlight the mechanism by which D11-PROTAC degrades STAT3 protein, depending on the binding of D11-PROTAC to STAT3 and VHL, followed by proteasomal degradation.

### Specificity of D11-PROTAC for degradation of STAT3 protein

The specific interaction of STAT3’s DBD with particular DNA sequences, typically resembling TTTCN3GTAA (where N represents any nucleotide), is facilitated by the unique non-structural region within STAT3’s DBD [34]. This distinct recognition capability differentiates STAT3 from other STAT family members. Consequently, the designed D11-PROTAC, targeted against the specific DNA decoy that binds to STAT3’s DBD, exhibits selective recognition and degradation of STAT3 protein. The selectivity of D11-PROTAC for degrading STAT3 protein in HeLa and MCF-7 cells was assessed, revealing efficient and specific induction of STAT3 protein degradation without affecting the expression levels of other STAT family members, as confirmed by WB analysis (Figs. 2M, N, S15, and S16). Furthermore, to evaluate the potential application of D11-PROTAC in other cancer types, we tested its efficacy in the colorectal cancer (CRC) cell line Caco-2. The results demonstrated that D11-PROTAC effectively degraded STAT3 in Caco-2 cells without affecting the expression of other STAT family members. These findings support the potential of D11-PROTAC as a targeted therapeutic strategy for CRC, suggesting its broad applicability in the treatment of various STAT3-driven cancers (Fig. S17).

### D11-PROTAC inhibits tumor cell migration and invasion and induces cell cycle arrest

**Fig. 3:**
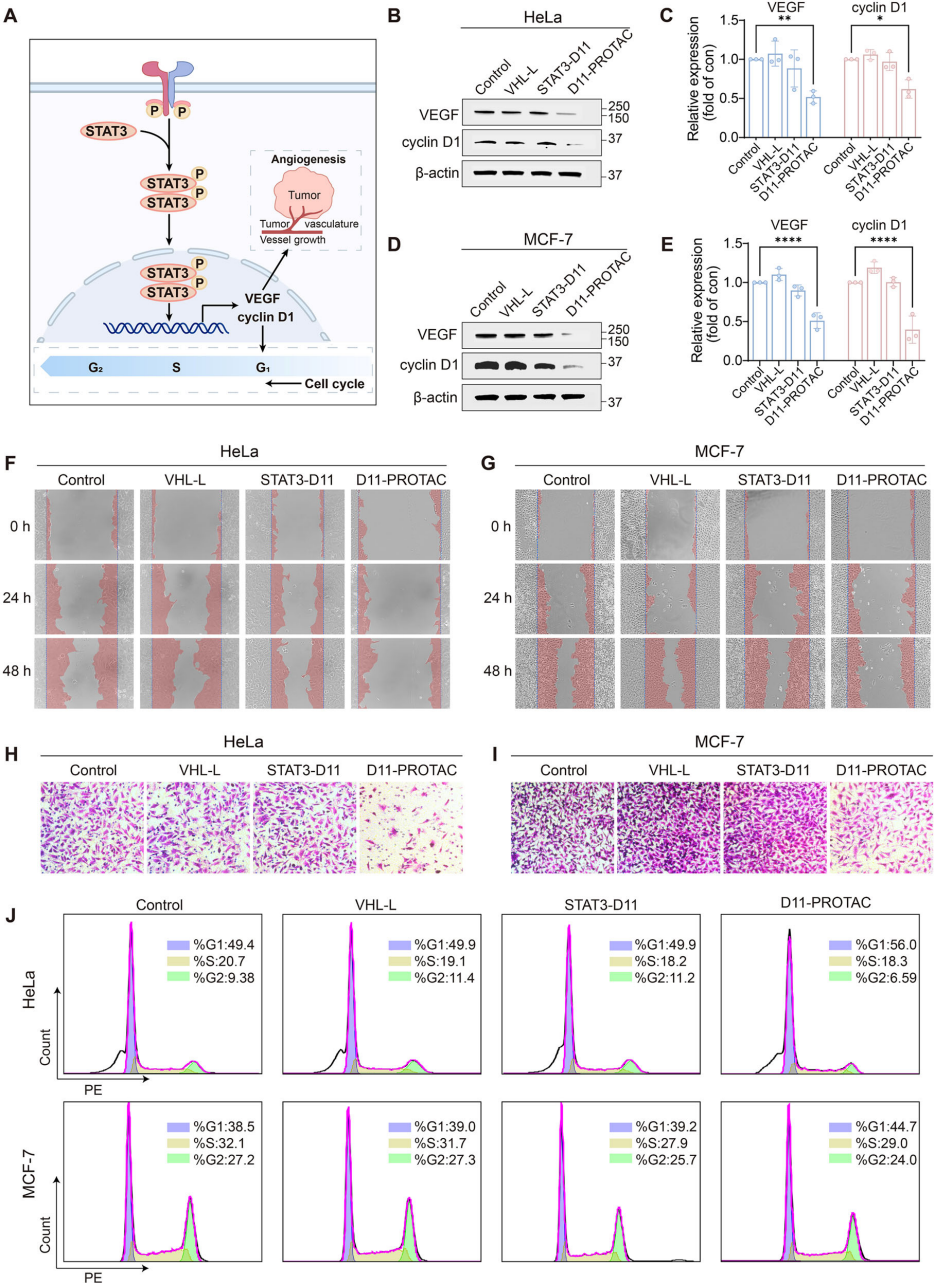
D11-PROTAC inhibits tumor cell migration and invasion and induces cell cycle arrest. **A** D11-PROTAC induces degradation of STAT3 protein resulting in downstream degradation of VEGF and cyclin D1 proteins. **B** HeLa and **D** MCF-7 cells were treated with VHL-L, STAT3-D11, and D11-PROTAC at a concentration of 1.5 µM for 24 h, and VEGF and cyclin D1 proteins were analyzed by WB. It is worth noting that the downstream proteins analyzed by WB in this study were run on the same SDS-PAGE gel and transferred onto the same PVDF membrane, thus the β-actin bands for the HeLa and MCF-7 cells in this Figure are the same as those in the subsequent Figs. 4B, D, and 5B, D. **C**, **E** Quantitative analysis of WB results using ImageJ. **F**, **G** Cell scratch assay: HeLa and MCF-7 cells migration at same concentrations (1.5 µM) of VHL-L, STAT3-D11, and D11-PROTAC, respectively. **H**, **I** Transwell invasion assay: HeLa and MCF-7 cells invasion at same concentrations (1.5 µM) of VHL-L, STAT3-D11, and D11-PROTAC for 24 h, respectively. **J** Cell cycle analysis: HeLa and MCF-7 cells cycle analysis at same concentrations (1.5 µM) of VHL-L, STAT3-D11, and D11-PROTAC for 24 h, respectively. The data are presented as the mean: SD values; *n* = 3. *P < 0.05, **P < 0.01, ***P < 0.001, and ****P < 0.0001 vs. the control group.

Downregulation of STAT3 can decrease vascular endothelial growth factor (VEGF), crucial for tumor vitality and angiogenesis [35]. Reduced VEGF expression from impaired STAT3 activity may inhibit angiogenesis (Fig. 3A), limiting blood and nutrient supply, ultimately affecting tumor growth and metastasis. To assess the impact of STAT3 downregulation on VEGF expression, we performed quantitative PCR (qPCR) analysis. The results showed a significant reduction in VEGF mRNA levels in both HeLa and MCF-7 cells following D11-PROTAC treatment (Figs. S18). WB analysis further confirmed a marked decrease in VEGF protein levels (Figs. 3B–E and S19, S20). With the confirmed downregulation of STAT3 and subsequent decrease in VEGF expression, the impact of D11-PROTAC on the migration and invasion abilities of HeLa and MCF-7 cells was assessed. Cell migration and invasion often occur in the process of metastasis of malignant tumor cells, thus it is generally believed that inhibiting tumor cell migration and invasion is an effective therapeutic means in tumor therapy [36]. A cell scratch assay, simulating cell migration on a 2D plane [37], showed noticeable suppression of cell migration after 48 h of D11-PROTAC treatment (Figs. 3F, G). This highlights D11-PROTAC’s significant inhibitory effect on tumor cell migration. For cell invasion, a transwell invasion assay was used, where tumor cells migrate through a membrane coated with Matrigel, mimicking the extracellular matrix [37]. The assay showed a marked reduction in the number of invasive cells in D11-PROTAC-treated groups (Figs. 3H, I). This demonstrates that D11-PROTAC significantly reduces the invasive abilities of HeLa and MCF-7 cells.

Additionally, reduced STAT3 levels have been shown to suppress cyclin D1 expression, a protein crucial for cell cycle progression, particularly the transition from G1 to S phase [38]. Thus, downregulating STAT3 can regulate the cell cycle by affecting cyclin D1 levels. The qPCR and WB analysis revealed that D11-PROTAC administration reduces cyclin D1 expression in HeLa and MCF-7 cells (Figs. 3B–E and S18–S20). To evaluate the impact of D11-PROTAC on the cell cycle, flow cytometry was employed. Figure 3J demonstrates that D11-PROTAC treatment significantly increased the proportion of HeLa and MCF-7 cells in the G0/G1 phase while decreasing the S phase. This indicates that D11-PROTAC induces cell cycle arrest, preventing cells from progressing to the S phase for DNA replication and maintaining them in the G0/G1 phase.

### D11-PROTAC inhibits tumor cell activity and induces cell apoptosis

**Fig. 4:**
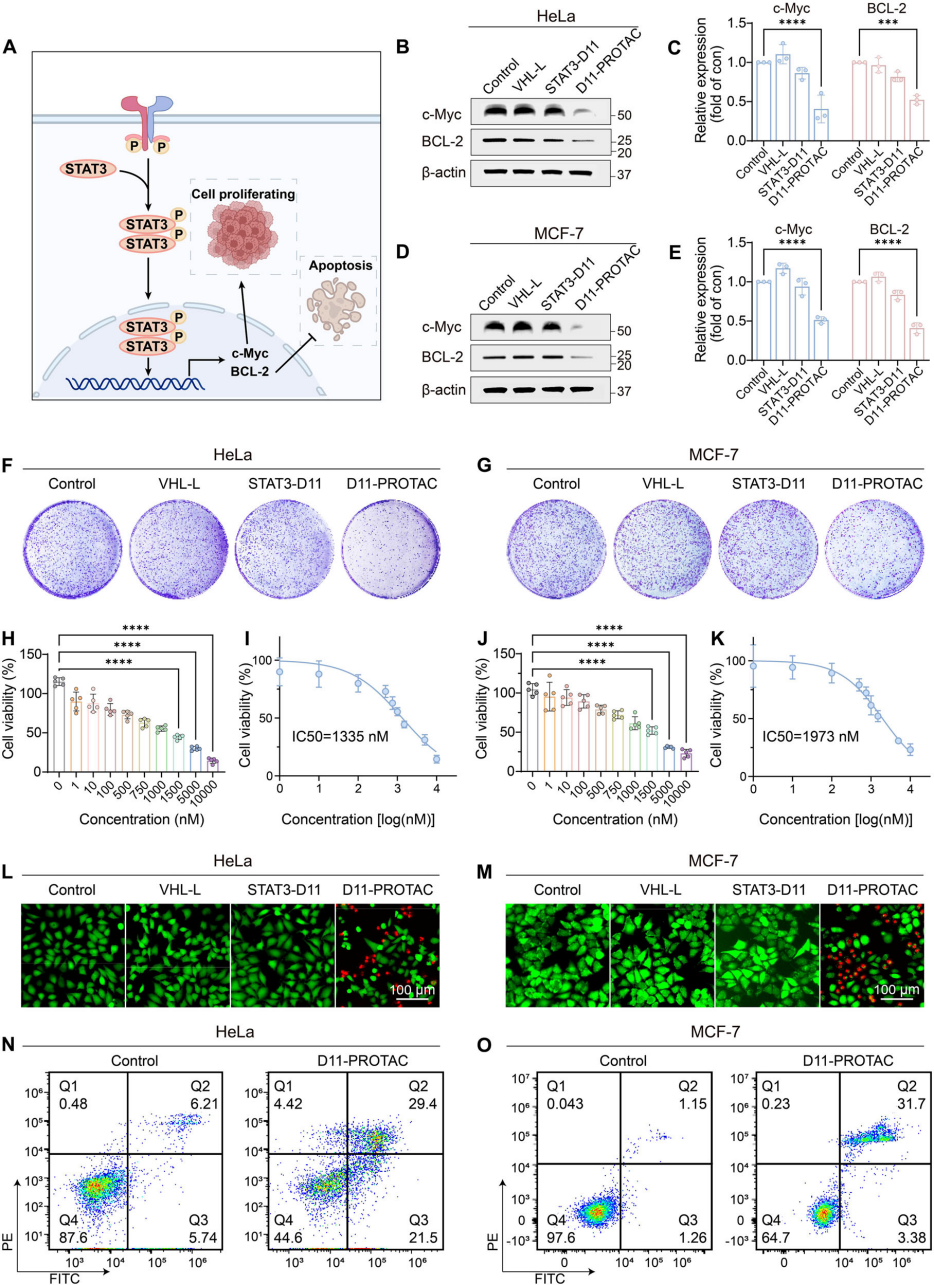
D11-PROTAC inhibits tumor cell activity and induces cell apoptosis. **A** D11-PROTAC induces degradation of STAT3 protein resulting in downstream degradation of c-Myc and BCL-2 proteins. **B** HeLa and **D** MCF-7 cells were treated with VHL-L, STAT3-D11, and D11-PROTAC at a concentration of 1.5 µM for 24 h, and c-Myc and BCL-2 proteins were analyzed by WB (*n* = 3). **C**, **E** Quantitative analysis of WB results using ImageJ. **F**, **G** Colony formation assay: HeLa and MCF-7 cells proliferation at same concentrations (1.5 µM) of VHL-L, STAT3-D11, and D11-PROTAC, respectively. **H**–**K** CCK-8 assay: HeLa and MCF-7 cells viability at different concentrations of D11-PROTAC for 48 h (*n* = 5). **L**, **M** HeLa and MCF-7 cells were stained with a live/dead cell viability/cytotoxicity kit after treatment with VHL-L, STAT3-D11, and D11-PROTAC for 48 h, respectively. **N**, **O** D11-PROTAC treatment induced cell apoptosis of HeLa and MCF-7 cells. The data are presented as the mean: SD values. *P < 0.05, **P < 0.01, ***P < 0.001, and ****P < 0.0001 vs. the control group.

The STAT3 signaling pathway activates c-Myc transcription, increasing c-Myc levels (Fig. 4A). As a transcription factor, c-Myc regulates multiple genes and is often overactivated in cancers, leading to abnormal cell proliferation [39]. Therefore, promoting STAT3 degradation can suppress c-Myc transcription and reduce cancer cell activity. The qPCR and WB results (Figs. 4B–E and S18–S20) showed that D11-PROTAC treatment led to decreased c-Myc expression. To confirm the anti-proliferative effects of D11-PROTAC via c-Myc downregulation, a clonal formation assay was performed in HeLa and MCF-7 cells, revealing significant inhibition of cell proliferation compared to control groups VHL-L and STAT3-D11 (Figs. 4F, G). The antitumor activity was further assessed using the cell counting kit-8 (CCK-8) assay, with D11-PROTAC showing IC50 values of 1335 nM and 1973 nM for HeLa and MCF-7 cells, respectively (Fig. 4H–K). Evaluating drug toxicity to normal cells is vital for clinical development, especially in cancer therapies. While STAT3 is essential for the growth of tumor cells, previous studies have shown that STAT3 is dispensable for the growth of normal cells [10]. Two normal cell lines (LO2 and MCF-10A) were chosen to test the biocompatibility of D11-PROTAC. CCK-8 assays on these cell lines confirmed good biocompatibility and no significant toxicity, supporting the potential of D11-PROTAC as a safe antitumor agent (Fig. S21). Calcein-AM and propidium iodide (PI) staining confirmed a decrease in live (green) cells and an increase in dead (red) cells in the D11-PROTAC-treated group (Figs. 4L, M). These results collectively validated the capability of D11-PROTAC to inhibit tumor cell proliferation and activity. To investigate the growth inhibition mechanism, flow cytometry was used to evaluate apoptosis. D11-PROTAC treatment significantly induced apoptosis in HeLa and MCF-7 cells (Fig. 4N, O). This effect is likely attributed to the downregulation of STAT3, leading to a decrease in the expression of the downstream gene BCL-2 and facilitating apoptosis [40]. The qPCR and WB analysis validated the ability of D11-PROTAC to downregulate the crucial anti-apoptotic protein BCL-2 in HeLa and MCF-7 cells, thereby enhancing apoptotic potential (Figs. 4B–E and S18–S20).

### D11-PROTAC inhibits tumor cell immune escape

**Fig. 5:**
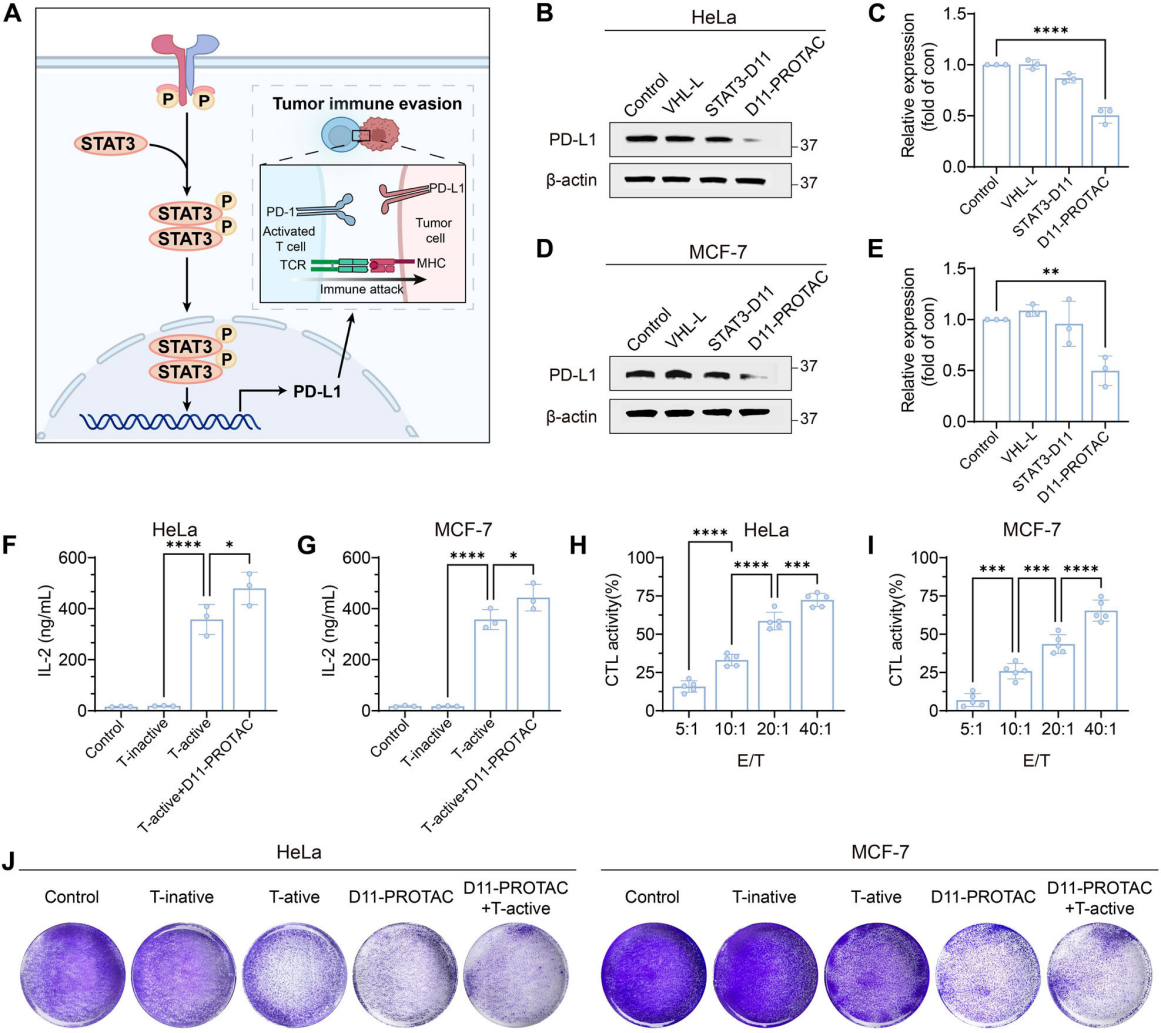
D11-PROTAC inhibits tumor cell immune escape. **A** D11-PROTAC induces degradation of STAT3 protein resulting in downstream degradation of PD-L1 protein. **B**, **D** HeLa or MCF-7 cells were treated with VHL-L, STAT3-D11, and D11-PROTAC at a concentration of 1.5 µM for 24 h, and PD-L1 protein were analyzed by WB. **C**, **E** Quantitative analysis of WB results using ImageJ. **F**, **G** IL-2 levels in the supernatant were measured by ELISA kits (*n* = 3). **H**, **I** Activated Jurkat T cells (effector cells) were cocultured with HeLa or MCF-7 cells (target cells) at different *E*/*T* ratios of 5:1, 10:1, 20:1, and 40:1 for 24 h to assess the cytotoxic activity of Jurkat T cells (*n* = 5). **J** HeLa or MCF-7 cells, pretreated with D11-PROTAC for 24 h, were cocultured with activated Jurkat T cells (ratio = 1:10) for 24 h and subsequently stained with crystal violet for imaging. The data are presented as the mean: SD values. *P < 0.05, **P < 0.01, ***P < 0.001, and ****P < 0.0001 vs. the control group.

The proximal region of the PD-L1 promoter is continuously occupied by STAT3, which directly regulates the expression of PD-L1 (Fig. 5A) [41]. The qPCR and WB results confirmed that D11-PROTAC, by degrading STAT3 protein, reduced PD-L1 expression (Fig. 5B–E and S18–S20). The reduction in PD-L1 expression contributes to the inhibition of tumor immune evasion. To investigate the inhibitory effects of D11-PROTAC on tumor immune evasion and its ability to reverse the negative impact of cancer cells on T cells, an in vitro model was established by co-culturing tumor cells with activated Jurkat cells. IL-2, a signature cytokine for activated T cells, was used as a biomarker to monitor T cell activation and proliferation [42]. The results demonstrated a significant increase in IL-2 levels in the supernatant after incubation with D11-PROTAC (Fig. 5F, G). Further, to assess the antitumor effects of activated Jurkat T cells against tumor cells post-D11-PROTAC treatment, Jurkat T cells (effectors, E) were co-cultured with HeLa or MCF-7 cells (targets, T). A dose-dependent increase in Jurkat T cell activity against target cells was observed following D11-PROTAC incubation, with significantly higher activity at E/T ratios of 20:1 and 40:1 (Fig. 5H, I). Moreover, activated Jurkat T cells showed superior antitumor efficacy against tumor cells treated with D11-PROTAC compared to untreated cells, indicating enhanced antitumor effects of activated Jurkat T cells following D11-PROTAC treatment (Fig. 5J).

### In vivo antitumor evaluation

**Fig. 6:**
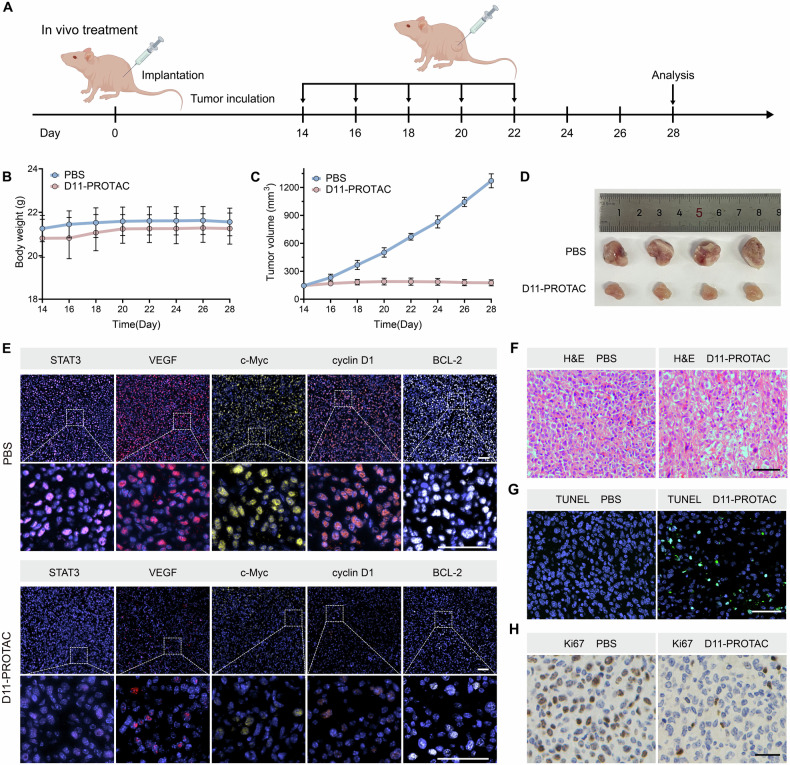
The in vivo antitumor efficacy of D11-PROTAC in mice bearing MCF-7 xenografts. **A** Schematic illustration of antitumor treatment. **B** Changes in the body weight of xenografted mice during the 15-day period of treatment with the different formulations indicated (*n* = 5). **C** Changes in the tumor volume during the 15-day period of treatment via intratumoral injection at a Polyacrylamide gel electrophoresis (PAGE)dosing frequency of every other day (*n* = 5). **D** Representative photos of tumors harvested on the 28th day. **E** Immunofluorescence staining of STAT3 and downstream proteins (VEGF, cyclin D1, c-Myc, and BCL-2) after 15-day period of treatment. **F** H&E and **G** TUNEL stained slices of tumor tissues from different groups, gathered at the end of treatment. **H** Micrographs of tumor tissue sections with Ki67 staining from the treatment groups. Scale bar: 50 μm.

Because of the promising antitumor performance of D11-PROTAC in vitro, an MCF-7 tumor-bearing mouse model was established by subcutaneous injection of MCF-7 cells into the right thigh of the mice to study its antitumor effects in vivo (Fig. 6A). First, Cy5-modified D11-PROTAC was prepared to monitor the distribution of D11-PROTAC in mice after intratumoral injection. Fluorescence imaging was performed at different post-injection times. Cy5 fluorescence remained present at 24 h (Fig. S22), demonstrating that D11-PROTAC could remain in tumor tissues over a prolonged duration. Subsequently, when the tumor volume reached ≈150 mm^3^, the mice were randomly divided into two groups (PBS and D11-PROTAC). Each mouse was injected intratumorally every other day with 100 μL of either PBS or D11-PROTAC (10 μM/dose). Over the 15 days of treatment, all mice were fed under the same conditions and their body weights and tumor volumes were measured every 2 days. As shown in Fig. 6B, D11-PROTAC showed good tolerability, with no significant weight loss or adverse reactions. The PBS group showed a significant increase in tumor volume and D11-PROTAC effectively suppressed the tumor growth (Fig. 6C, D).

To evaluate the detailed mechanism underlying D11-PROTAC antitumor performance, tumors were harvested after the treatment was completed, and then subjected to immunofluorescence staining of STAT3 and downstream proteins (VEGF, cyclin D1, c-Myc, BCL-2, and PD-L1) and TUNEL assays, as well as hematoxylin and eosin (H&E) and ki67 immunohistochemical staining. Immunofluorescence staining demonstrated significant reductions in STAT3 and downstream proteins (VEGF, cyclin D1, c-Myc, BCL-2, and PD-L1) post-treatment, confirming D11-PROTAC’s ability to downregulate these proteins in tumor tissues (Figs. 6E and S23), aligning with its cellular effects. As clearly depicted in Fig. 6F, H&E staining of tumor sections displayed the largest injury area in the D11-PROTAC group compared to PBS, suggesting incremental necrosis induced by D11-PROTAC. Furthermore, TUNEL assays (Fig. 6G) demonstrated the exceptional antitumor efficiency of D11-PROTAC in vivo with conspicuous green fluorescence signals due to intense apoptosis. Ki67 staining (Fig. 6H) revealed the lowest proliferation signal in treated tumors, indicating effective inhibition of tumor cell proliferation. To further probe the biocompatibility of D11-PROTAC, tumors were stripped, and the major organs of the mice were sliced and stained with H&E (Fig. S24). The results showed no obvious physiological damage, suggesting that there was no substantial systemic toxicity associated with D11-PROTAC use in vivo. Overall, D11-PROTAC effectively targets STAT3, demonstrating robust antitumor effects and promising safety as a potential antitumor therapeutic.

## Discussion

In summary, we have successfully devised, synthesized, characterized, and validated the D-PROTAC for precise degradation of STAT3 protein. By triggering the formation of a ternary complex involving STAT3, D-PROTAC, and the E3 ligases VHL, STAT3 undergoes efficient degradation in cancer cells via a mechanism reliant on both the E3 ligases VHL and the proteasome. Our findings provide compelling evidence to support the effectiveness of D-PROTAC as a potent, selective, and efficient STAT3 protein degrader based on the PROTAC approach. Notably, D-PROTAC also elicits downregulation of various downstream target genes regulated by STAT3, resulting in cell cycle arrest, apoptosis promotion, inhibition of tumor migration and proliferation, and suppression of tumor immune evasion. Consequently, D-PROTAC enhances the antitumor effects and holds significant therapeutic potential for the treatment of diseases governed by STAT3. Our research establishes a foundation for the clinical advancement of “undruggable” STAT3 protein degraders, offering promising therapeutic benefits.

## Supplementary information

Supporting Information

western blots original data

QPCR Original Data

## Data Availability

All data supporting the findings of this study are available within the Article and its Supplementary Information.
