# Correction: Decoy-PROTAC for specific degradation of “Undruggable” STAT3 transcription factor

**DOI:** 10.1038/s41419-025-07801-y

**Published:** 2025-07-23

**Authors:** Shiqing Li, Xin Wang, Jiabao Huang, Xiuping Cao, Yana Liu, Shiyan Bai, Tao Zeng, Qi Chen, Chunsen Li, Chunhua Lu, Huanghao Yang

**Affiliations:** 1 New Cornerstone Science Laboratory, MOE Key Laboratory for Analytical Science of Food Safety and Biology, College of Chemistry, Fuzhou University, Fuzhou, People’s Republic of China; 2 State Key Laboratory of Structural Chemistry, Fujian Institute of Research on the Structure of Matter, Chinese Academy of Sciences, Fuzhou, Fujian People’s Republic of China; 3 Interdisciplinary Institute for Medical Engineering, Fuzhou University, Fuzhou, People’s Republic of China

**Keywords:** Proteolysis, Cancer therapy

Correction to: *Cell Death and Disease* 10.1038/s41419-025-07535-x, published online 21 March 2025

Following publication of this article, the authors realized there was an inadvertent error in Fig. 3I that needed correction. In Fig. 3I, the image of the Control group was mistakenly used due to initial mislabeling. The correct image of the Control group is provided in the corrected Fig. 3I below. This inadvertent error did not impact the conclusions of the article.

**Original Figure 3** 
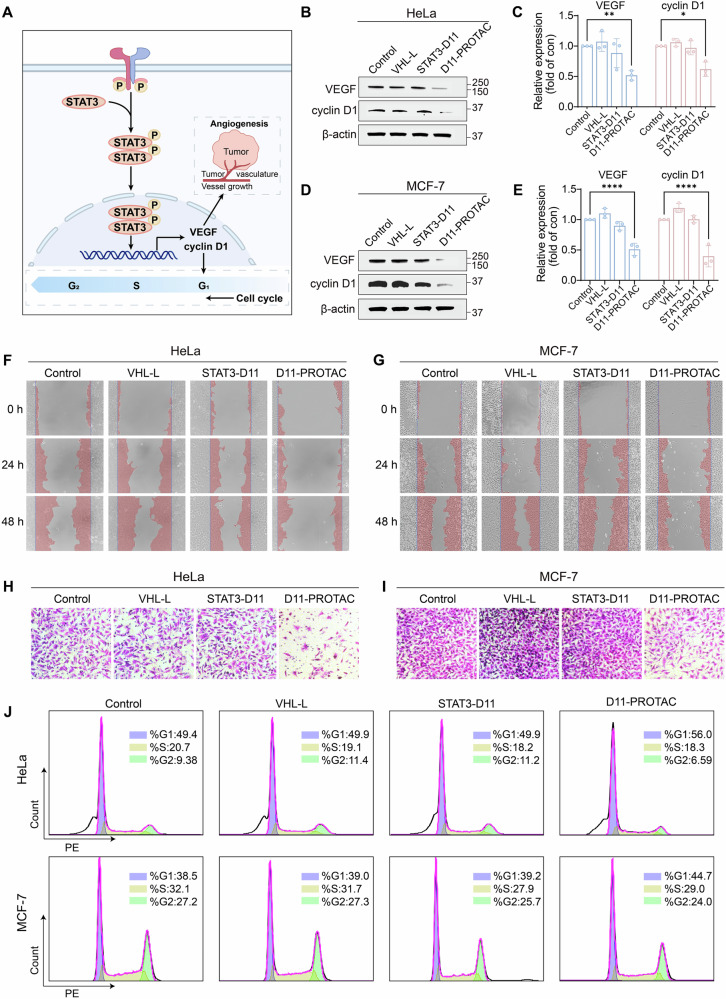


**Amended Figure 3** 
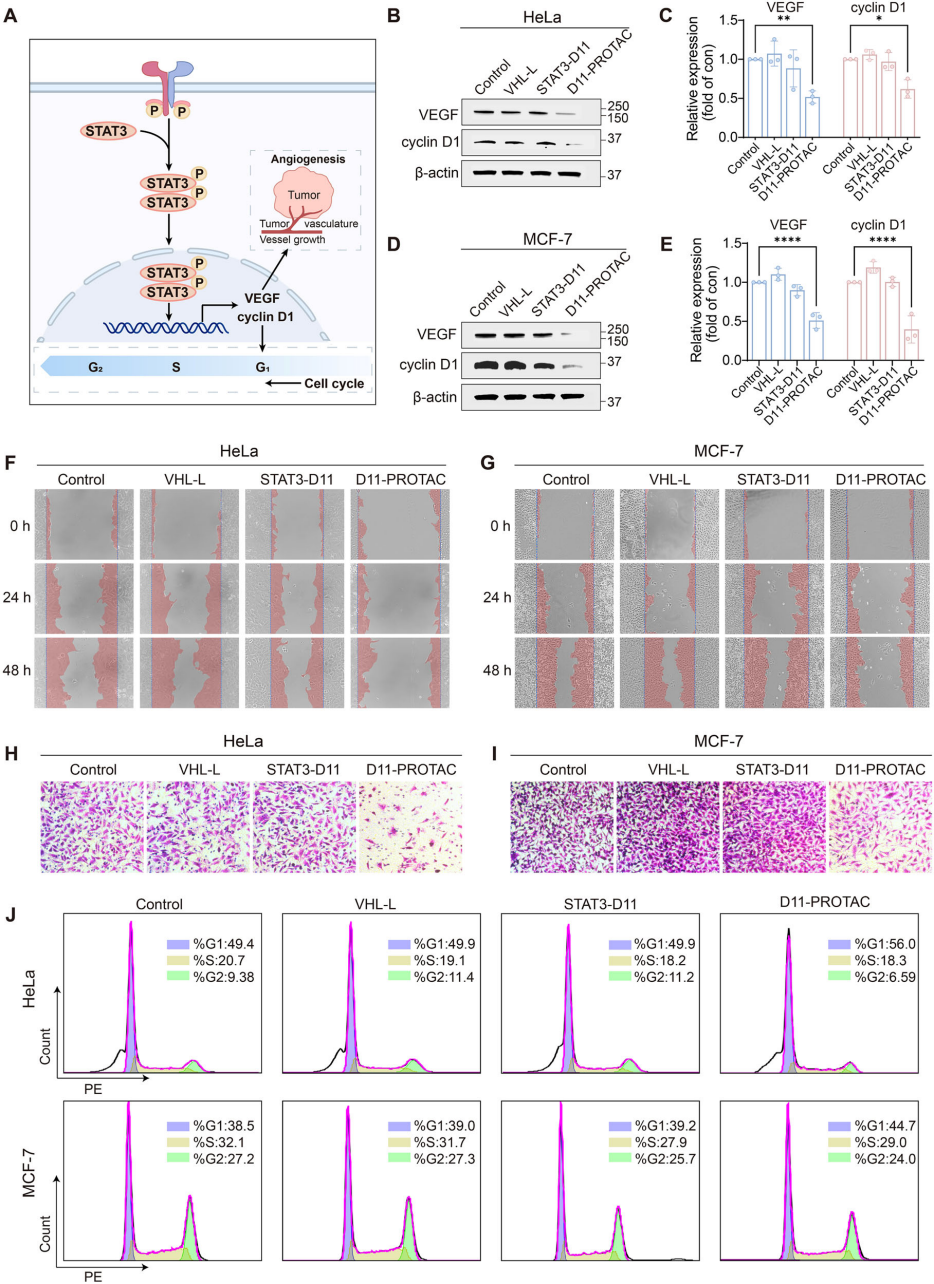


The original article has been corrected.

