# Supplementary material for: Decoy-PROTAC for specific degradation of “Undruggable” STAT3 transcription factor
Source: Cell Death Dis. 2025 Mar 21;16(1):197. doi: 10.1038/s41419-025-07535-x (PMC11928565; doi:10.1038/s41419-025-07535-x)
Supplement: Supplementary file 1 — Supporting Information [file 41419_2025_7535_MOESM1_ESM.pdf]

Supporting Information

Decoy-PROTAC for Specific Degradation of “Undruggable” STAT3 Transcription Factor

Shiqing Li[1‡], Xin Wang[1‡], Jiabao Huang[2], Xiuping Cao[1], Yana Liu[1], Shiyan Bai[1], Tao Zeng[1], Qi Chen[3], Chunsen Li[2], Chunhua Lu[1]*, and Huanghao Yang[1]*

[1] New Cornerstone Science Laboratory, MOE Key Laboratory for Analytical Science of Food Safety and Biology, College of Chemistry, Fuzhou University, Fuzhou 350108, People's Republic of China

[2] State Key Laboratory of Structural Chemistry, Fujian Institute of Research on the Structure of Matter, Chinese Academy of Sciences, Fuzhou, Fujian 350002, People's Republic of China

[3] Interdisciplinary institute for medical engineering, Fuzhou University, Fuzhou 350108, People's Republic of China

‡ These authors contributed equally.

*Corresponding Author: Chunhua Lu, E-mail: chunhualu@fzu.edu.cn; Huanghao Yang, E-mail: hhyang@fzu.edu.cn

Table of Contents

Table S1. Primer sequences……………….....…………………………………….………………….S3
Fig. S1. Schematic diagrams of the six STAT3-D………………………………………………….S4
Fig. S2. The mass spectra of the six D-PROTACs………………………………………………….S4
Fig. S3. Polyacrylamide gel electrophoresis image of the six STAT3-D………………………….....S5
Fig. S4. Three replicates of experiments showing the change trends of STAT3 and pSTAT3 proteins degradation in HeLa cell by D-PROTACs with different linker lengths………………………….....S6
Fig. S5. Three replicates of experiments showing the change trends of STAT3 and pSTAT3 proteins degradation in MCF-7 cell by D-PROTACs with different linker lengths…………………………S7
Fig. S6. Three replicates of experiments showing the change trends of STAT3 and pSTAT3 proteins degradation in HeLa cell by D11-PROTAC with different concentrations……………………….....S8
Fig. S7. Three replicates of experiments showing the change trends of STAT3 and pSTAT3 proteins degradation in MCF-7 cell by D11-PROTAC with different concentrations………………………S9
Fig. S8. Three replicates of experiments showing the change trends of STAT3 and pSTAT3 proteins degradation in HeLa cell by D11-PROTAC with different times……………………………….S10
Fig. S9. Three replicates of experiments showing the change trends of STAT3 and pSTAT3 proteins degradation in MCF-7 cell by D11-PROTAC with different times……………………………….S11
Fig. S10. Three replicates of experiments showing the change trends of STAT3 and pSTAT3 proteins degradation in HeLa cell by treatment with D11-PROTAC and proteasome inhibitor MG132…..S12
Fig. S11. Three replicates of experiments showing the change trends of STAT3 and pSTAT3 proteins degradation in MCF-7 cell by treatment with D11-PROTAC and proteasome inhibitor MG132…S13
Fig. S12. Three replicates of experiments showing the change trends of STAT3 and pSTAT3 proteins degradation in HeLa and MCF-7 cell by treatment with D11-PROTAC and VHL ligand………...S14
Fig. S13. Incubation of VHL-L, STAT3-D11, and D11-PROTAC in HeLa cell revealed that only D11-PROTAC was capable of degrading STAT3 protein……………………………………...S15
Fig. S14. Incubation of VHL-L, STAT3-D11, and D11-PROTAC in MCF-7 cell revealed that only D11-PROTAC was capable of degrading STAT3 protein……………………………………...S16
Fig. S15. Three replicates of experiments showing the change trends of STAT3, pSTAT3 and other STAT family members proteins degradation in HeLa cell by treatment with D11-PROTAC……..S17
Fig. S16. Three replicates of experiments showing the change trends of STAT3, pSTAT3 and other STAT family members proteins degradation in MCF-7 cell by treatment with D11-PROTAC…...S18
Fig. S17. Three replicates of experiments showing the change trends of STAT3, pSTAT3 and other STAT family members proteins degradation in Caco-2 cell by treatment with D11-PROTAC…...S19
Fig. S18. The crucial downstream STAT3 targets mRNA level in HeLa and MCF-7 cells (actin as housekeeping gene)……………………………………………………………………………S20
Fig. S19. Three replicates of experiments showing the change trends of STAT3, pSTAT3 and other crucial downstream STAT3 targets proteins degradation in HeLa cell……………………………S21
Fig. S20. Three replicates of experiments showing the change trends of STAT3, pSTAT3 and other crucial downstream STAT3 targets proteins degradation in MCF-7 cell…………..………………S22
Fig. S21. LO2 and MCF-10A cells viability at different concentrations of D11-PROTAC………S23
Fig. S22. In vivo fluorescence imaging………………………………………………………..S23
Fig. S23. Immunofluorescence STAT3 and PD-L1 staining images of tumors…………………..S23
Fig. S24. H&E staining of the major organs from different groups……………………………….S24

Table S1. Primer sequences.

**DNA Oligonucleotides sequence**	
STAT3-D0	5'-AAACATTTCCCGTAAATCGAAAGATT<br>TACGGG AAATG-DBCO-3'
STAT3-D7	5'-AAACATTTCCCGTAAATCGAAAGATT<br>TACGGG AAATGAAAAAAA-DBCO-3'
STAT3-D11	5'-AAACATTTCCCGTAAATCGAAAGATT<br>TACGGG AAATGAAAAAAAAAAA-DBCO-3'
STAT3-D15	5'-AAACATTTCCCGTAAATCGAAAGATTTACGGG<br>AAATGAAAAAAAAAAAAAAA-DBCO-3'
STAT3-D19	5'-AAACATTTCCCGTAAATCGAAAGATTTACGGG<br>AAATGAAAAAAAAAAAAAAAAAAA-DBCO-3'
STAT3-D23	5'-AAACATTTCCCGTAAATCGAAAGATTTACGGG<br>AAATGAAAAAAAAAAAAAAAAAAAAAAA-DBCO-3'
STAT3-D27	5'-AAACATTTCCCGTAAATCGAAAGATTTACGGG<br>AAATGAAAAAAAAAAAAAAAAAAAAAAAAAAA-DBCO-3'
**QPCR primer sequences**	
VEGF-F(homo)	5'-ATCGAGTACATCTTCAAGCCAT-3'
VEGF-R(homo)	5'-GTGAGGTTTGATCCGCATAATC-3'
c-Myc-F(homo)	5'-CCTACCCTCTCAACGACAGC-3'
c-Myc-R(homo)	5'-TTGTTCCTCCTCAGAGTCGC-3'
cyclinD1-F(homo)	5'-CAGACCTTCGTTGCCCTCTG-3'
cyclinD1-R(homo)	5'-CAGTCCGGGTCACACTTGAT-3'
BCL-2-F(homo)	5'-GACTTCGCCGAGATGTCCAG-3'
BCL-2-R(homo)	5'-GAACTCAAAGAAGGCCACAATC-3'
PD-L1-F(homo)	5'-AGGCCGAAGTCATCTGGACA-3'
PD-L1-R(homo)	5'-TGTTGATTCTCAGTGTGCTGGT-3'
actin-F(Homo)	5'-TGACGTGGACATCCGCAAAG-3'
actin-R(Homo)	5'-CTGGAAGGTGGACAGCGAGG-3'

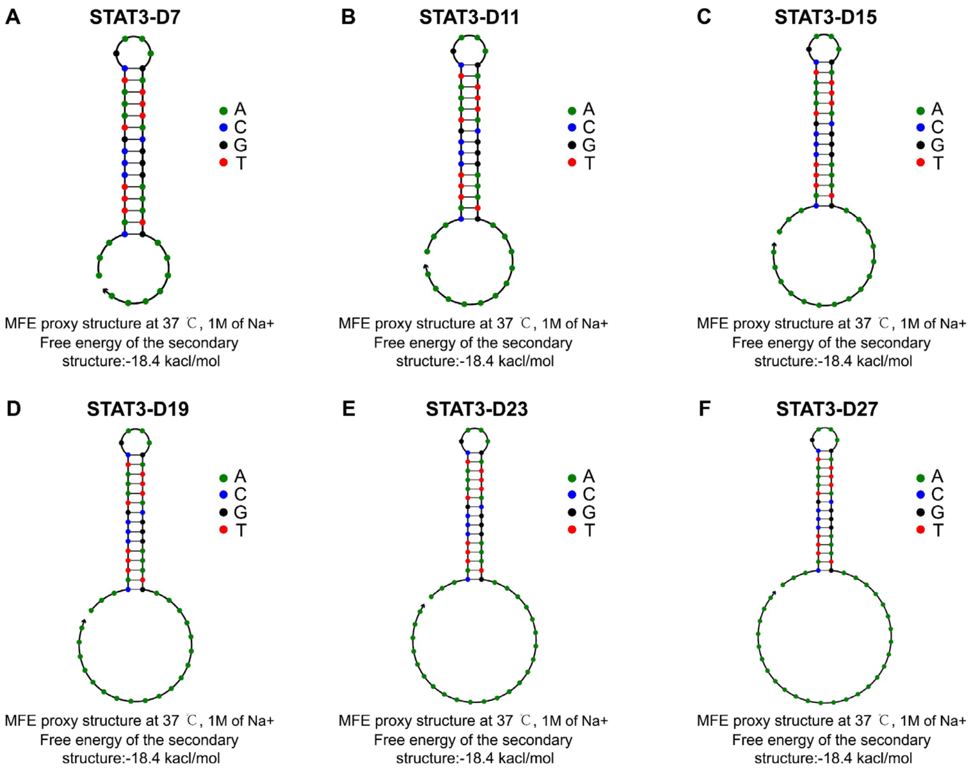

Fig. S1. Schematic diagrams of the six STAT3-D (referred to as STAT3-D7, STAT3-D11, STAT3-D15, STAT3-D19, STAT3-D23, STAT3-D27) hairpin structures.

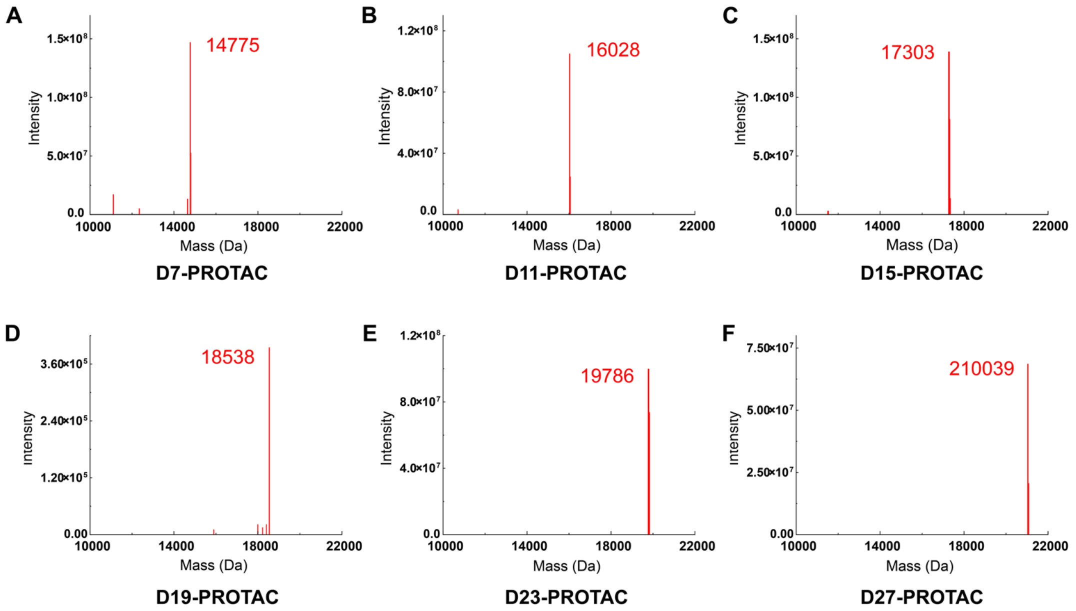

Fig. S2. The mass spectra of the six D-PROTACs (D7-PROTAC, D11-PROTAC, D15-PROTAC, D19-PROTAC, D23-PROTAC, D27-PROTAC).

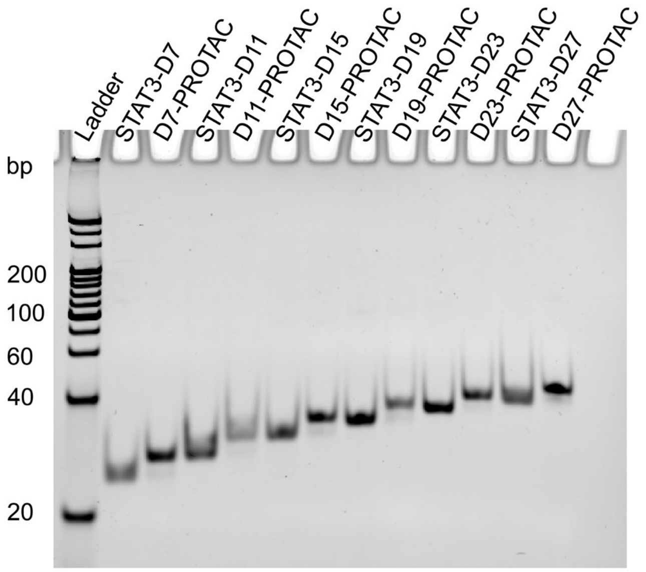

Fig. S3. Polyacrylamide gel electrophoresis image of the six STAT3-D (referred to as STAT3-D7, STAT3-D11, STAT3-D15, STAT3-D19, STAT3-D23, STAT3-D27) and six D-PROTACs (D7-PROTAC, D11-PROTAC, D15-PROTAC, D19-PROTAC, D23-PROTAC, D27-PROTAC).

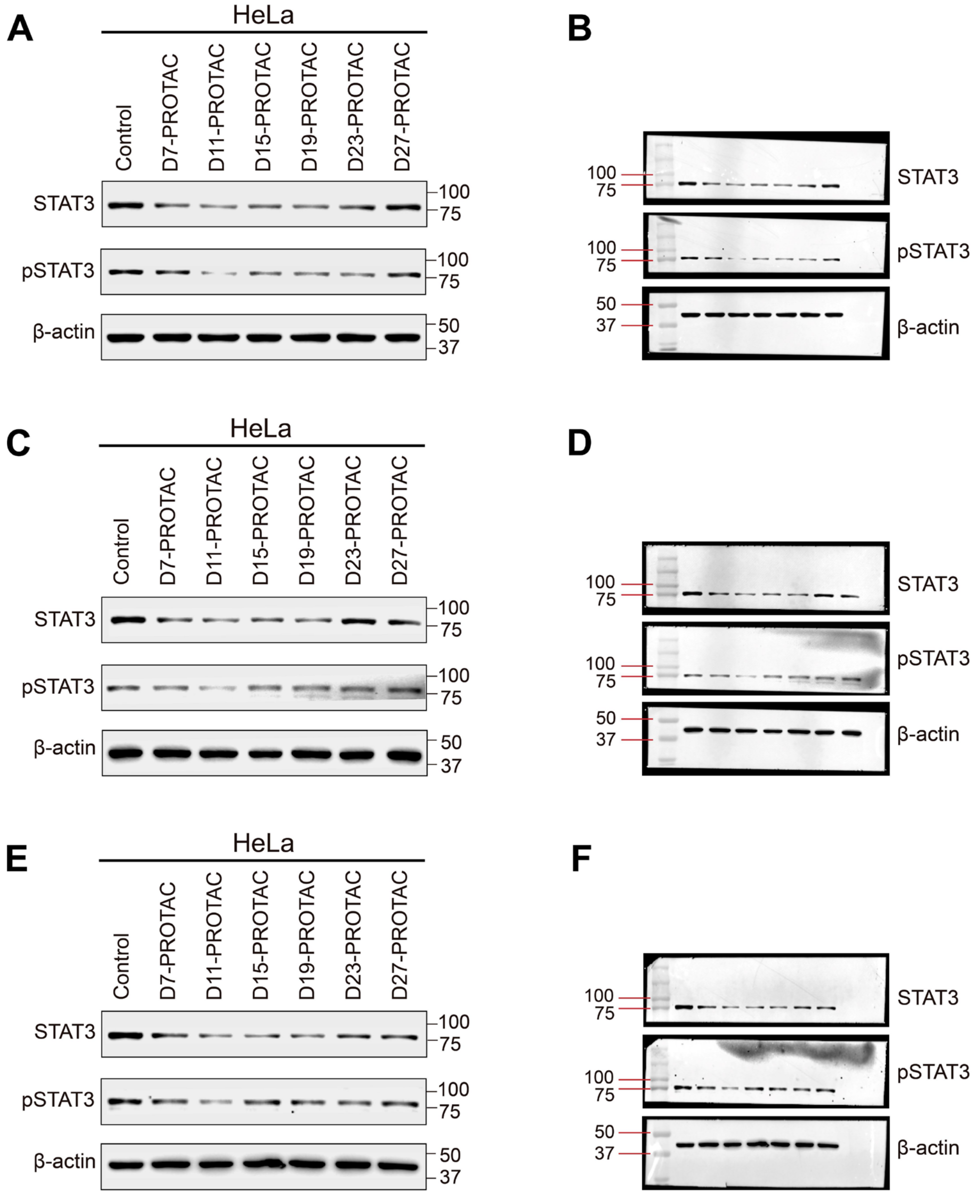

Fig. S4. (A, C, E) Three replicates of experiments showing the change trends of STAT3 and pSTAT3 proteins degradation in HeLa cell by D-PROTACs with different linker lengths (7, 11, 15, 19, 23, 27 bases). (B, D, F) Corresponding original data graphs to (A, C, E), displaying the merging of markers and protein bands. STAT3 and pSTAT3 proteins analyzed through WB in this research were run on the same SDS-PAGE gel and transferred to the same PVDF membrane. Following the assessment of pSTAT3 expression levels, the antibodies were stripped using a stripping buffer, re-blocked, and then re-incubated with STAT3 antibodies to detect STAT3 expression levels. **The data used in Fig. 1B in the main text is Fig. S4A.**

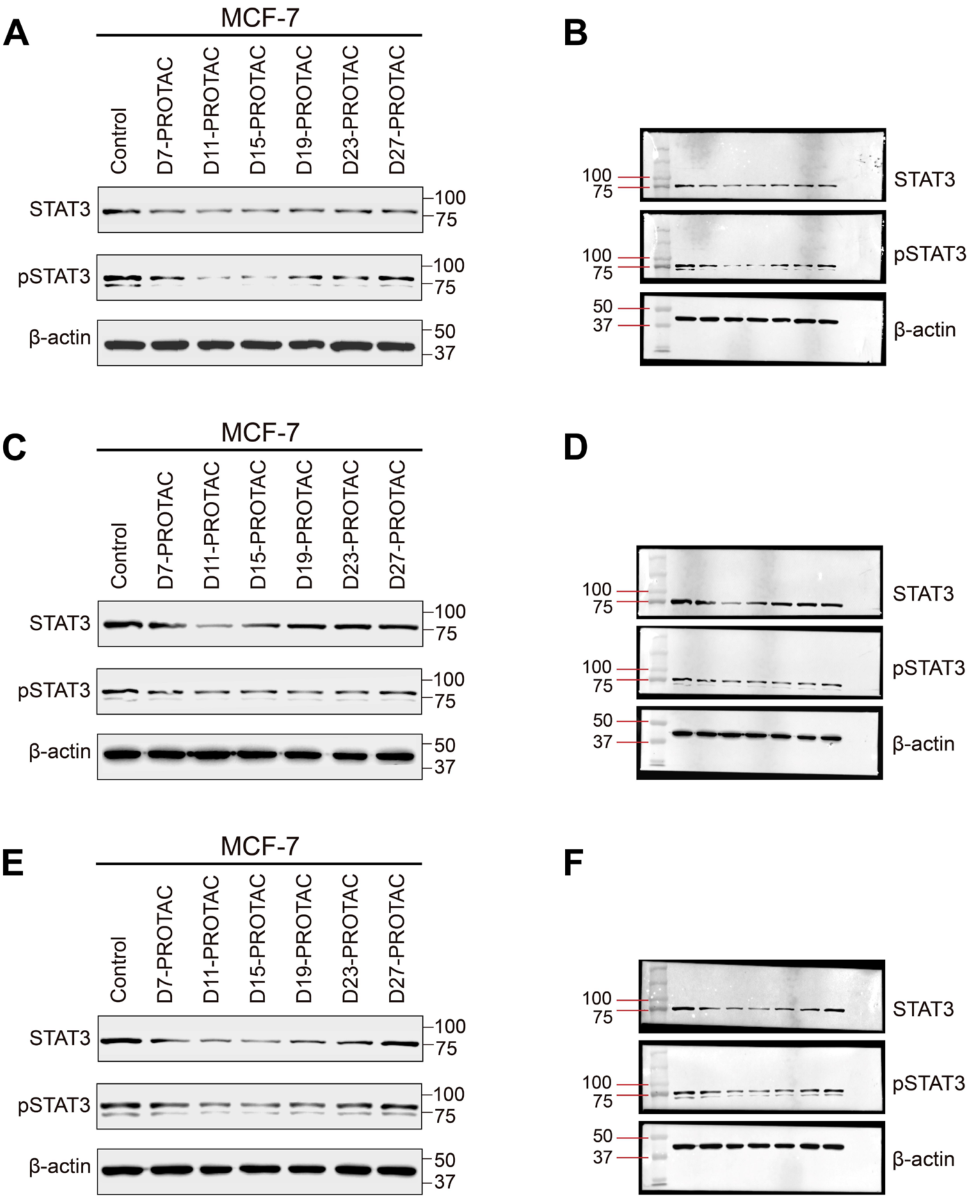

Fig. S5. (A, C, E) Three replicates of experiments showing the change trends of STAT3 and pSTAT3 proteins degradation in MCF-7 cell by D-PROTACs with different linker lengths (7, 11, 15, 19, 23, 27 bases). (B, D, F) Corresponding original data graphs to (A, C, E), displaying the merging of markers and protein bands. STAT3 and pSTAT3 proteins analyzed through WB in this research were run on the same SDS-PAGE gel and transferred to the same PVDF membrane. Following the assessment of pSTAT3 expression levels, the antibodies were stripped using a stripping buffer, re-blocked, and then re-incubated with STAT3 antibodies to detect STAT3 expression levels. **The data used in Fig. 1D in the main text is Fig. S5A.**

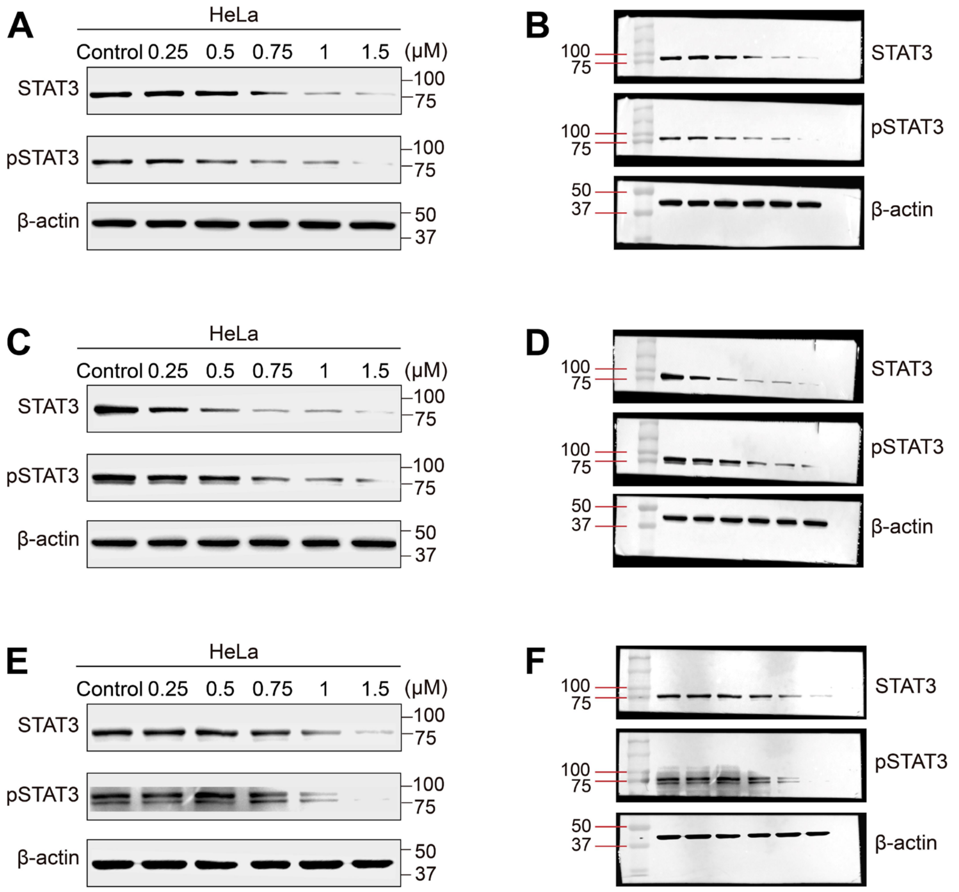

Fig. S6. (A, C, E) Three replicates of experiments showing the change trends of STAT3 and pSTAT3 proteins degradation in HeLa cell by D11-PROTAC with different concentrations (0.25, 0.5, 0.75, 1 and 1.5 μM). (B, D, F) Corresponding original data graphs to (A, C, E), displaying the merging of markers and protein bands. STAT3 and pSTAT3 proteins analyzed through WB in this research were run on the same SDS-PAGE gel and transferred to the same PVDF membrane. Following the assessment of pSTAT3 expression levels, the antibodies were stripped using a stripping buffer, re-blocked, and then re-incubated with STAT3 antibodies to detect STAT3 expression levels. **The data used in Fig. 2A in the main text is Fig. S6A.**

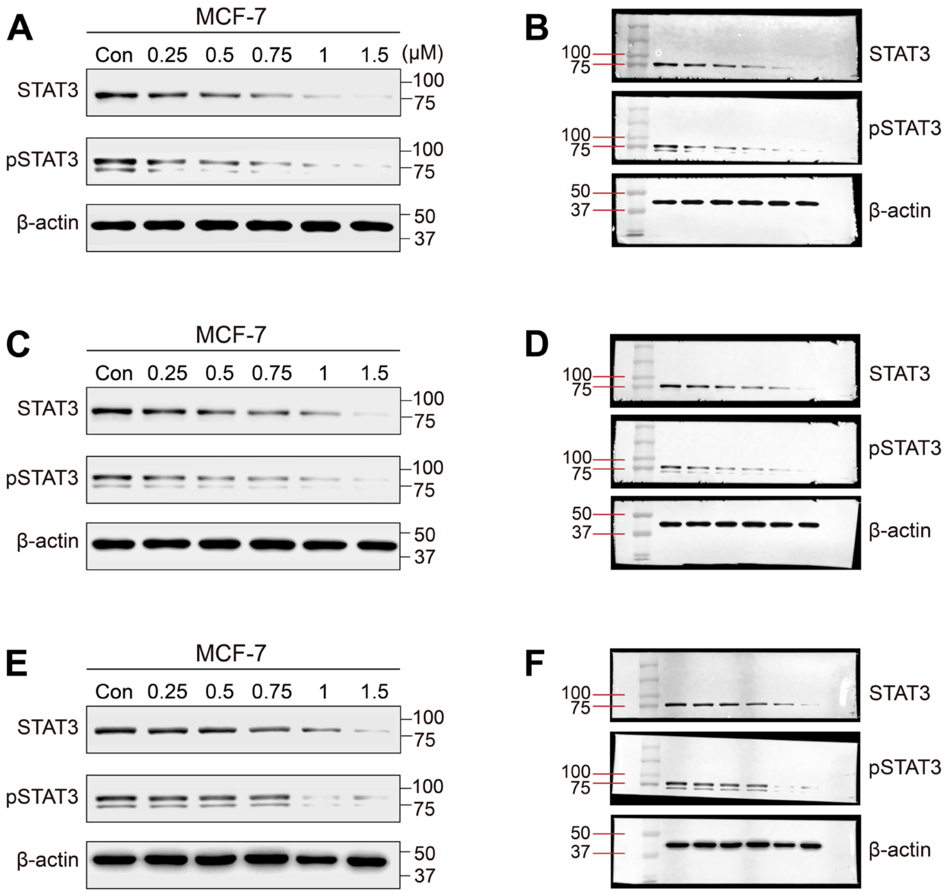

Fig. S7. (A, C, E) Three replicates of experiments showing the change trends of STAT3 and pSTAT3 proteins degradation in MCF-7 cell by D11-PROTAC with different concentrations (0.25, 0.5, 0.75, 1 and 1.5 μM). (B, D, F) Corresponding original data graphs to (A, C, E), displaying the merging of markers and protein bands. STAT3 and pSTAT3 proteins analyzed through WB in this research were run on the same SDS-PAGE gel and transferred to the same PVDF membrane. Following the assessment of pSTAT3 expression levels, the antibodies were stripped using a stripping buffer, re-blocked, and then re-incubated with STAT3 antibodies to detect STAT3 expression levels. **The data used in Fig. 2C in the main text is Fig. S7A.**

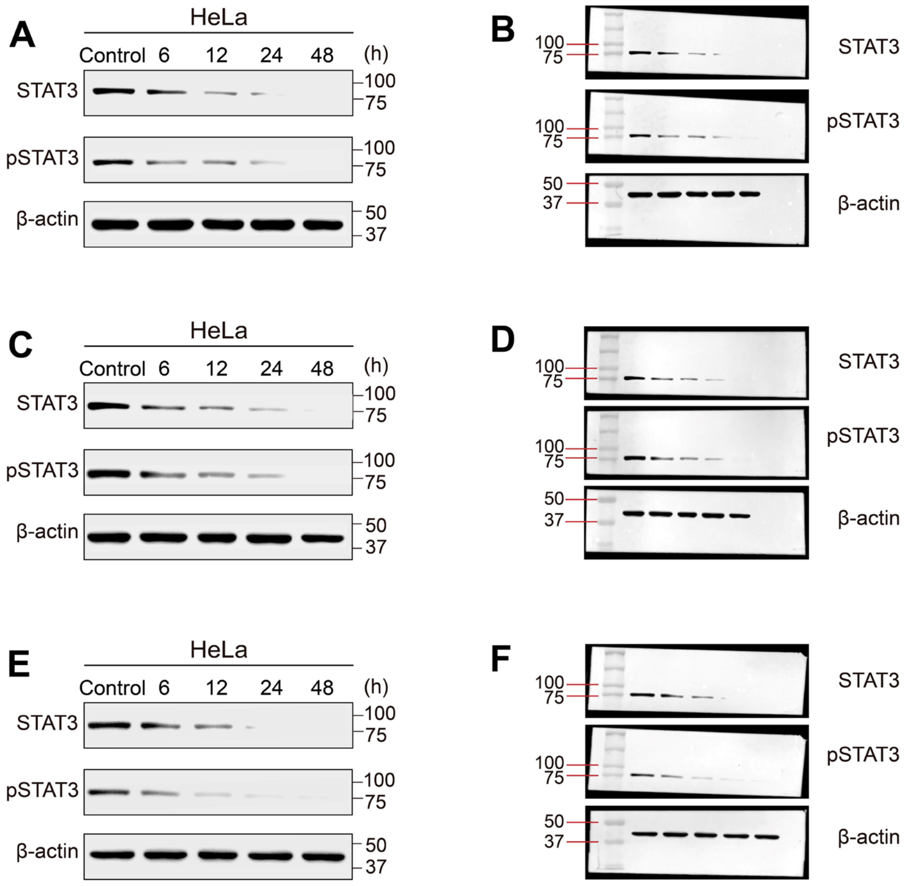

Fig. S8. (A, C, E) Three replicates of experiments showing the change trends of STAT3 and pSTAT3 proteins degradation in HeLa cell by D11-PROTAC with different times (6, 12, 24 and 48 h). (B, D, F) Corresponding original data graphs to (A, C, E), displaying the merging of markers and protein bands. STAT3 and pSTAT3 proteins analyzed through WB in this research were run on the same SDS-PAGE gel and transferred to the same PVDF membrane. Following the assessment of pSTAT3 expression levels, the antibodies were stripped using a stripping buffer, re-blocked, and then re-incubated with STAT3 antibodies to detect STAT3 expression levels. **The data used in Fig. 2E in the main text is Fig. S8A.**

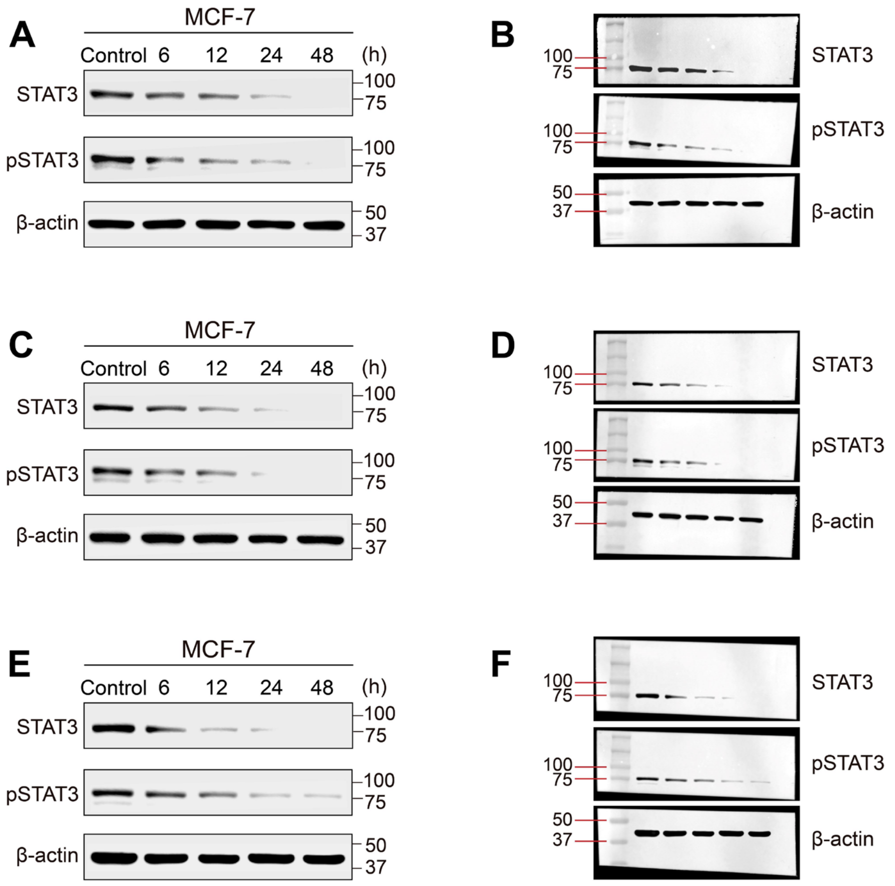

Fig. S9. (A, C, E) Three replicates of experiments showing the change trends of STAT3 and pSTAT3 proteins degradation in MCF-7 cell by D11-PROTAC with different times (6, 12, 24 and 48 h). (B, D, F) Corresponding original data graphs to (A, C, E), displaying the merging of markers and protein bands. STAT3 and pSTAT3 proteins analyzed through WB in this research were run on the same SDS-PAGE gel and transferred to the same PVDF membrane. Following the assessment of pSTAT3 expression levels, the antibodies were stripped using a stripping buffer, re-blocked, and then re-incubated with STAT3 antibodies to detect STAT3 expression levels. **The data used in Fig. 2G in the main text is Fig. S9A.**

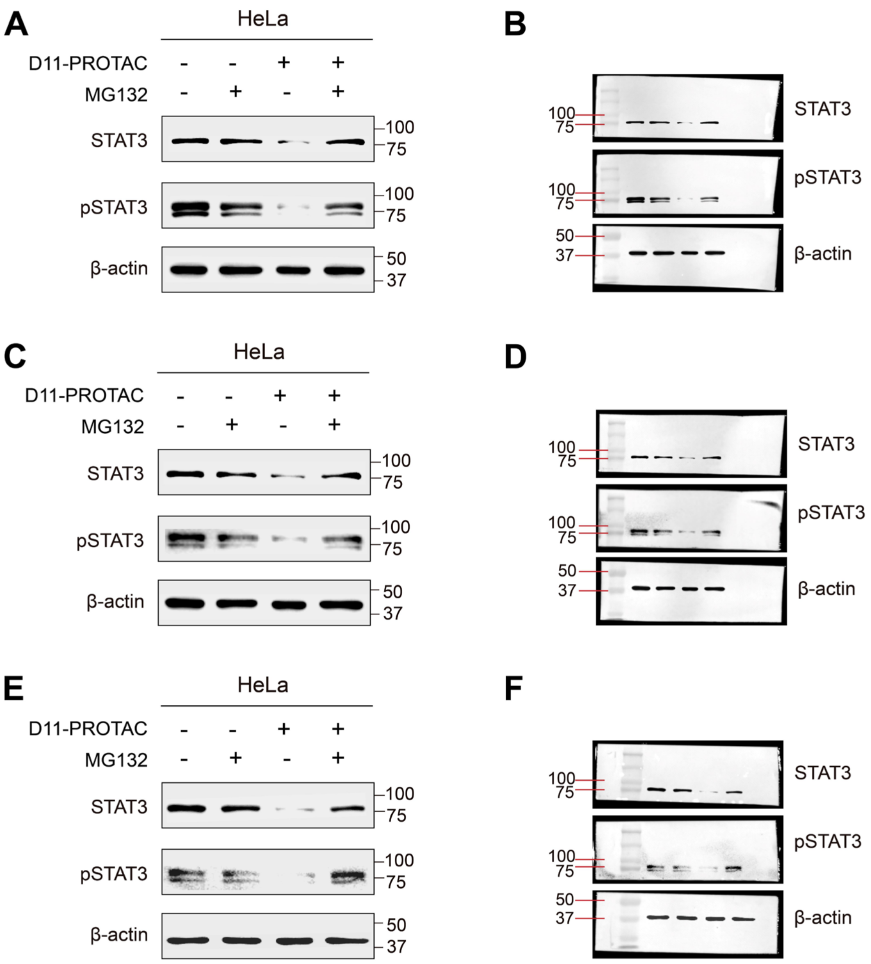

Fig. S10. (A, C, E) Three replicates of experiments showing the change trends of STAT3 and pSTAT3 proteins degradation in HeLa cell by treatment with D11-PROTAC and proteasome inhibitor MG132. (B, D, F) Corresponding original data graphs to (A, C, E), displaying the merging of markers and protein bands. STAT3 and pSTAT3 proteins analyzed through WB in this research were run on the same SDS-PAGE gel and transferred to the same PVDF membrane. Following the assessment of pSTAT3 expression levels, the antibodies were stripped using a stripping buffer, re-blocked, and then re-incubated with STAT3 antibodies to detect STAT3 expression levels. **The data used in Fig. 2I in the main text is Fig. S10A.**

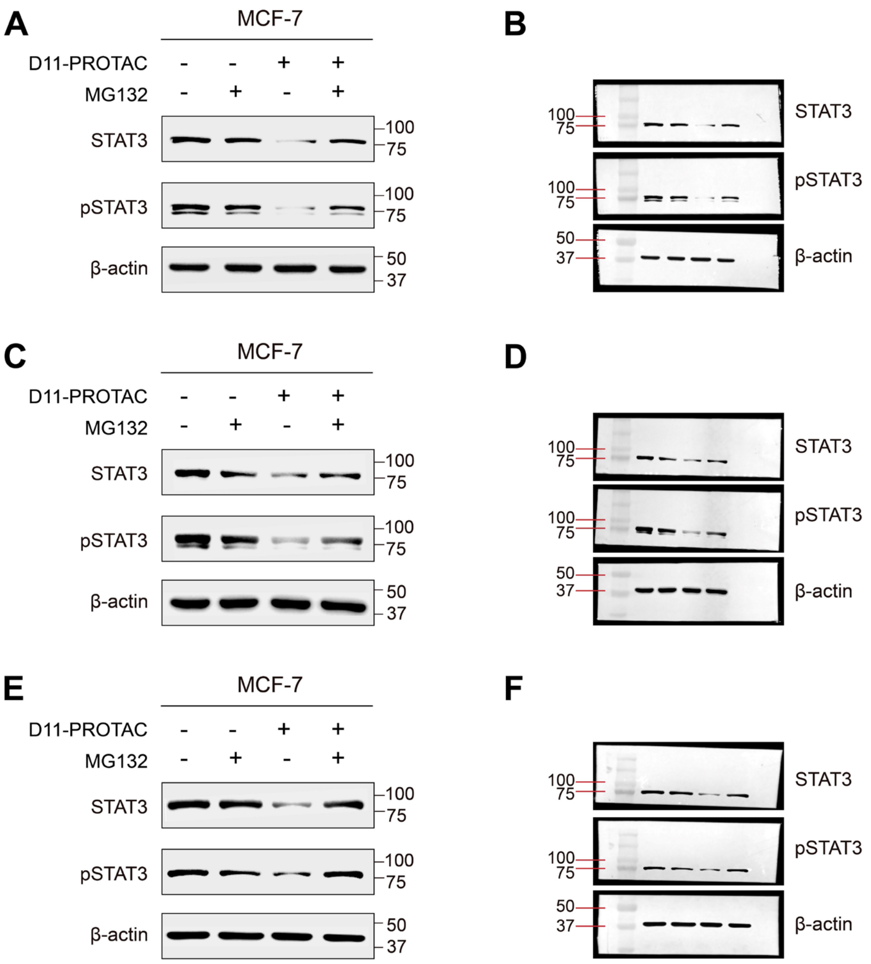

Fig. S11. (A, C, E) Three replicates of experiments showing the change trends of STAT3 and pSTAT3 proteins degradation in MCF-7 cell by treatment with D11-PROTAC and proteasome inhibitor MG132. (B, D, F) Corresponding original data graphs to (A, C, E), displaying the merging of markers and protein bands. STAT3 and pSTAT3 proteins analyzed through WB in this research were run on the same SDS-PAGE gel and transferred to the same PVDF membrane. Following the assessment of pSTAT3 expression levels, the antibodies were stripped using a stripping buffer, re-blocked, and then re-incubated with STAT3 antibodies to detect STAT3 expression levels. **The data used in Fig. 2K in the main text is Fig. S11A.**

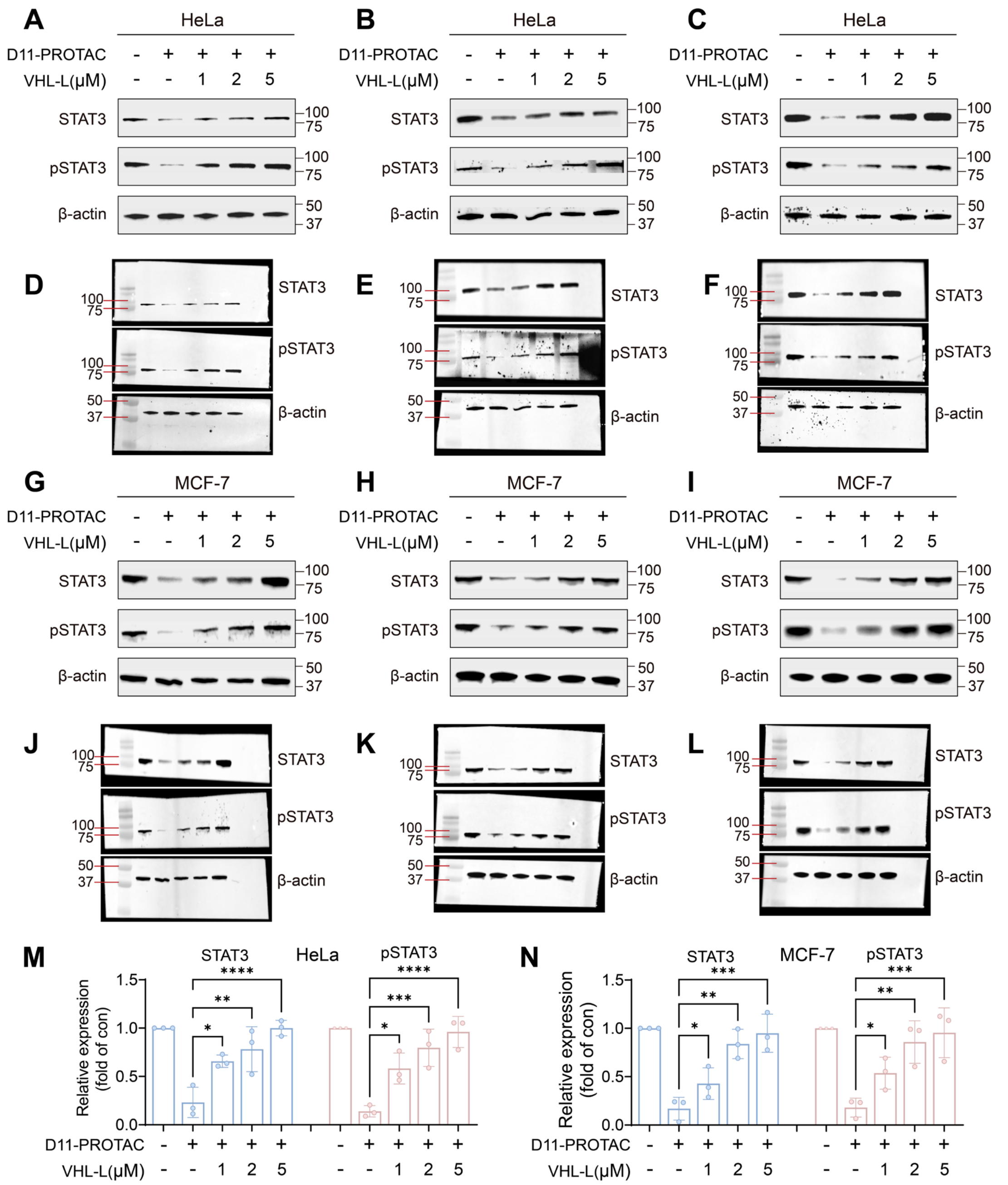

Fig. S12. (A-C, G-I) Three replicates of experiments showing the change trends of STAT3 and pSTAT3 proteins degradation in HeLa and MCF-7 cell by treatment with D11-PROTAC and VHL ligand (VHL-L). (D-F, J-L) Corresponding original data graphs to (A-C, G-I), displaying the merging of markers and protein bands. STAT3 and pSTAT3 proteins analyzed through WB in this research were run on the same SDS-PAGE gel and transferred to the same PVDF membrane. Following the assessment of pSTAT3 expression levels, the antibodies were stripped using a stripping buffer, re-blocked, and then re-incubated with STAT3 antibodies to detect STAT3 expression levels. The data are presented as the mean: SD values; $n = 3$. *$P < 0.05$, **$P < 0.01$, ***$P < 0.001$, and ****$P < 0.0001$ vs. the control group.

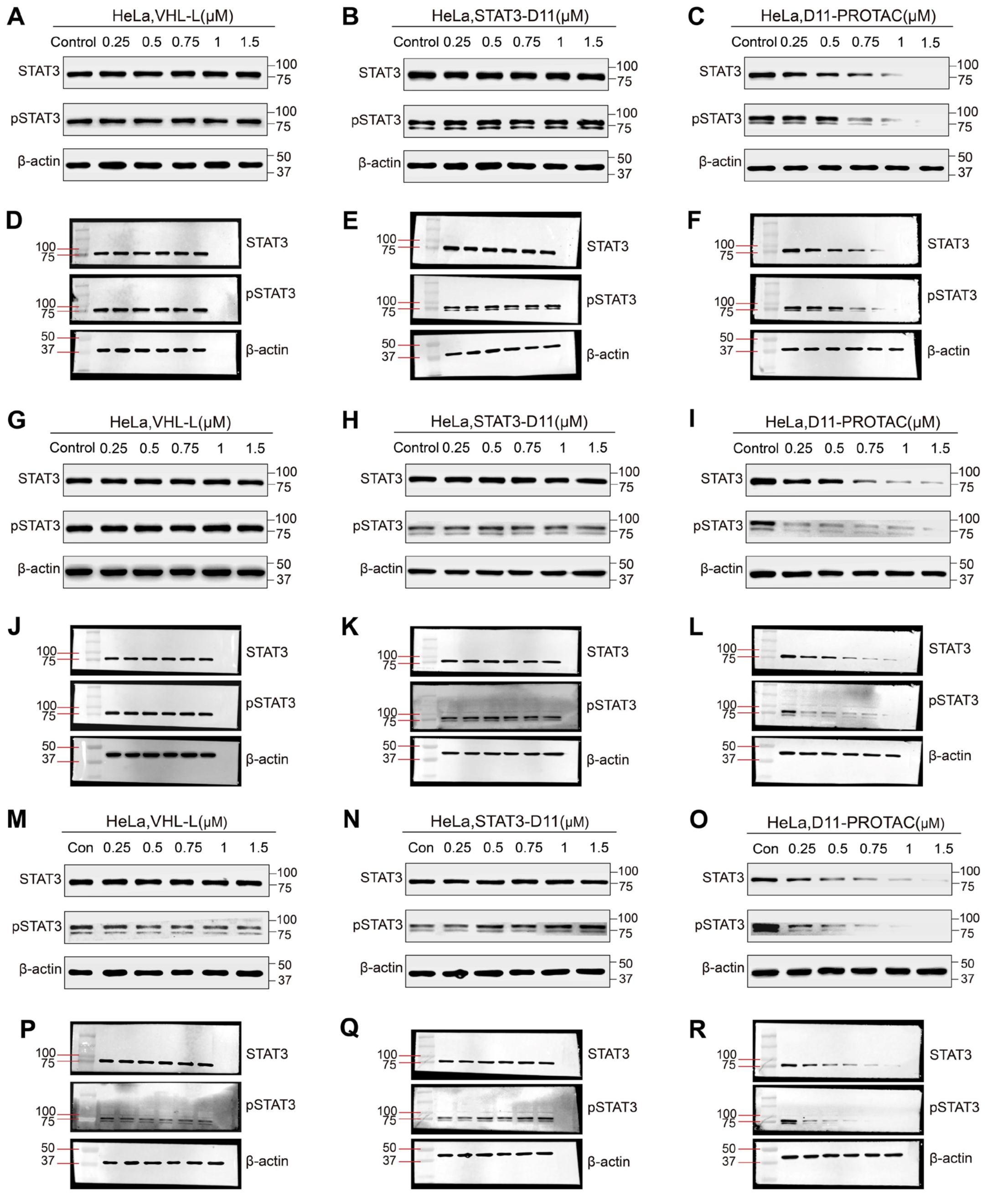

Fig. S13. Incubation of VHL-L, STAT3-D11, and D11-PROTAC in HeLa cell revealed that only D11-PROTAC was capable of degrading STAT3 protein. (A-C, G-I, M-O) The three replicates of experiments showing the change trends of STAT3 and pSTAT3 proteins degradation in HeLa cell by incubation of VHL-L, STAT3-D11, and D11-PROTAC at a concentration of 1.5 μM for 24 h and analyzed by WB. (D-F, J-L, P-R) Corresponding original data graphs to (A-C, G-I, M-O), displaying the merging of markers and protein bands. STAT3 and pSTAT3 proteins analyzed through WB in this research were run on the same SDS-PAGE gel and transferred to the same PVDF membrane. Following the assessment of pSTAT3 expression levels, the antibodies were stripped using a stripping buffer, re-blocked, and then re-incubated with STAT3 antibodies to detect STAT3 expression levels.

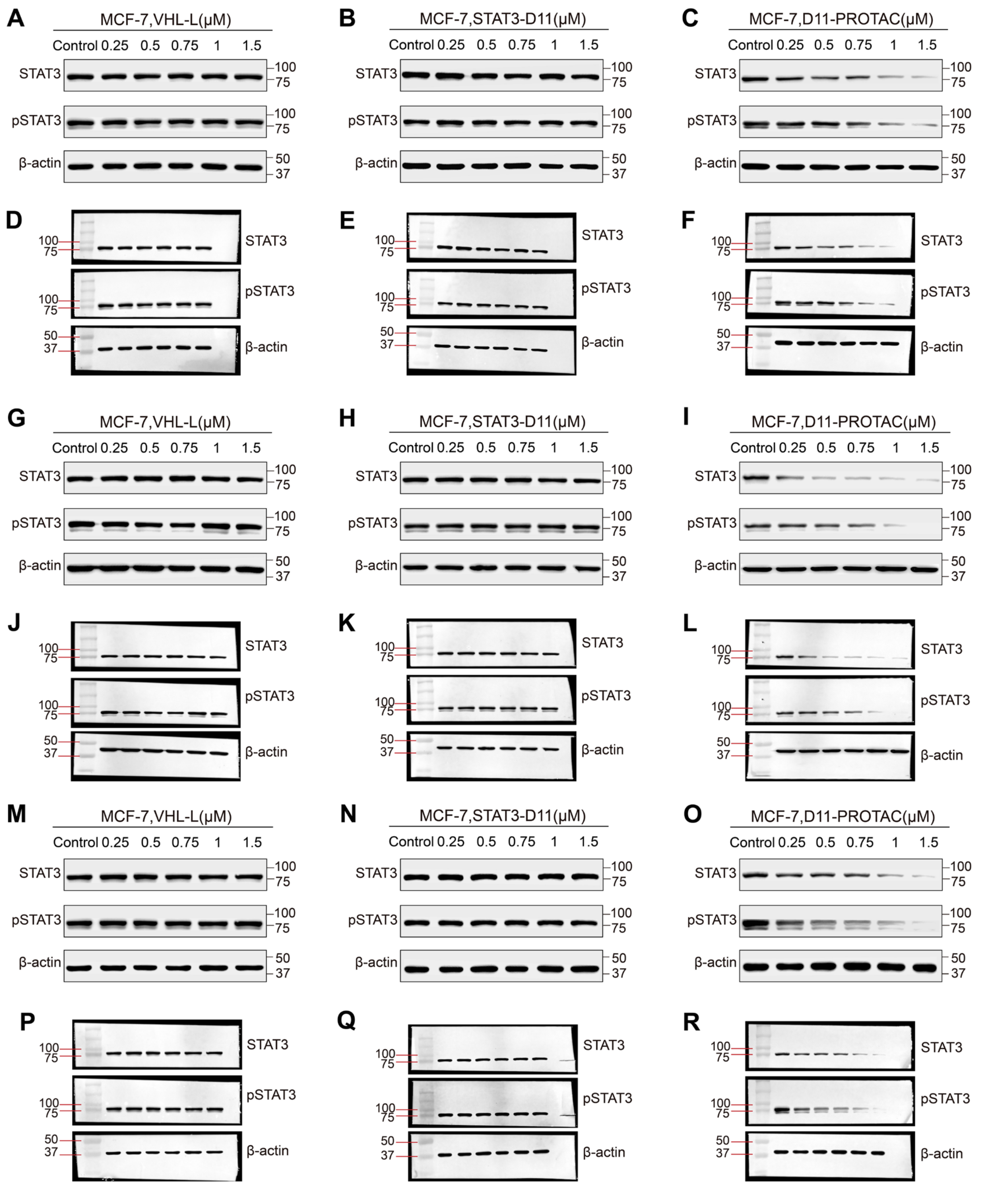

Fig. S14. Incubation of VHL-L, STAT3-D11, and D11-PROTAC in MCF-7 cell revealed that only D11-PROTAC was capable of degrading STAT3 protein. (A-C, G-I, M-O) The three replicates of experiments showing the change trends of STAT3 and pSTAT3 proteins degradation in MCF-7 cell by incubation of VHL-L, STAT3-D11, and D11-PROTAC at a concentration of 1.5 μM for 24 h and analyzed by WB. (D-F, J-L, P-R) Corresponding original data graphs to (A-C, G-I, M-O), displaying the merging of markers and protein bands. STAT3 and pSTAT3 proteins analyzed through WB in this research were run on the same SDS-PAGE gel and transferred to the same PVDF membrane. Following the assessment of pSTAT3 expression levels, the antibodies were stripped using a stripping buffer, re-blocked, and then re-incubated with STAT3 antibodies to detect STAT3 expression levels.

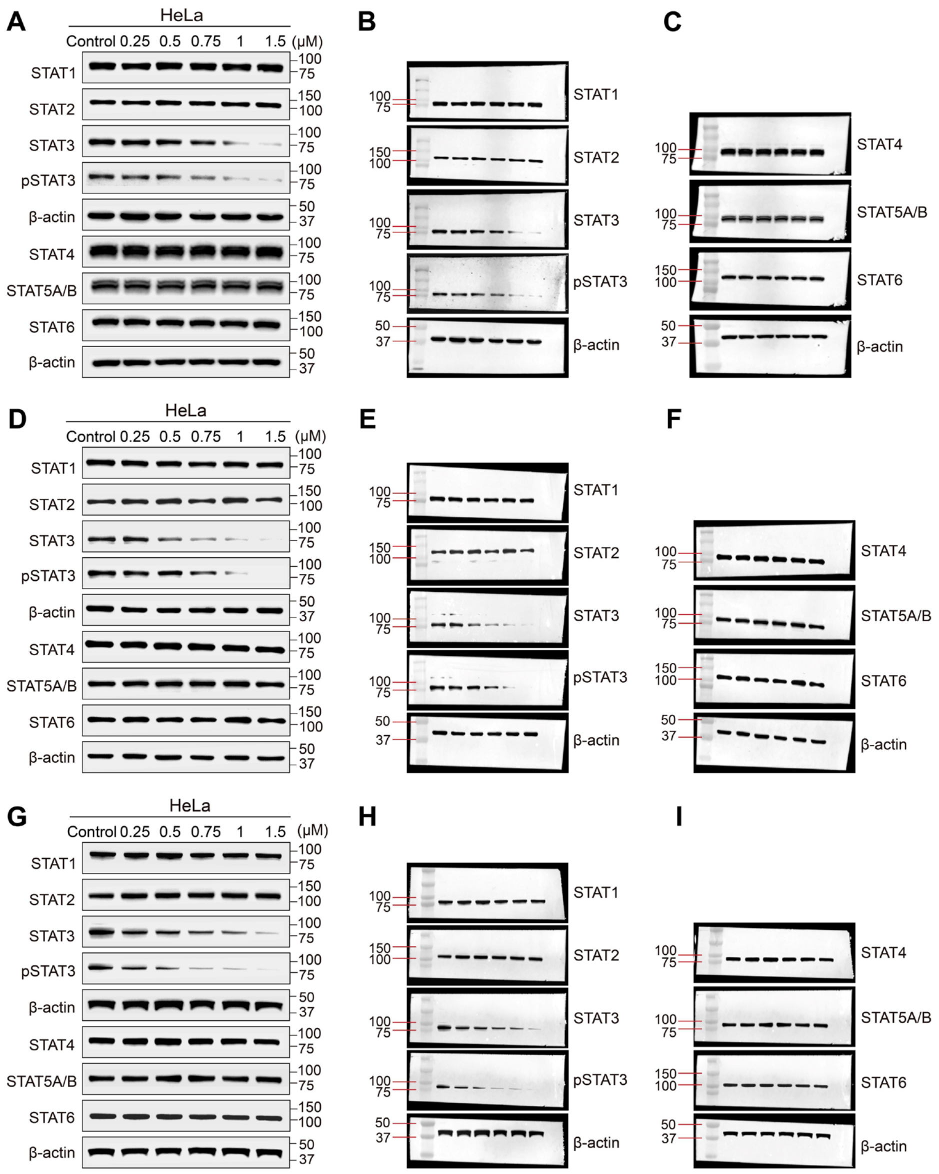

Fig. S15. (A, C, E) Three replicates of experiments showing the change trends of STAT3, pSTAT3 and other STAT family members proteins degradation in HeLa cell by treatment with D11-PROTAC. (B, D, F) Corresponding original data graphs to (A, C, E), displaying the merging of markers and protein bands. STAT1, STAT2, STAT3 and pSTAT3 proteins analyzed through WB in this research were run on the same SDS-PAGE gel and transferred to the same PVDF membrane. Following the assessment of pSTAT3 expression levels, the antibodies were stripped using a stripping buffer, re-blocked, and then re-incubated with STAT3 antibodies to detect STAT3 expression levels. The protein expression levels of STAT1 and STAT2 were detected sequentially following this procedure. STAT4, STAT5A/B and STAT6 were also imaged on the same PVDF membrane to detect their protein expression levels in the order of STAT4, STAT5A/B and STAT6. **The data used in Fig. 2M in the main text is Fig. S15A.**

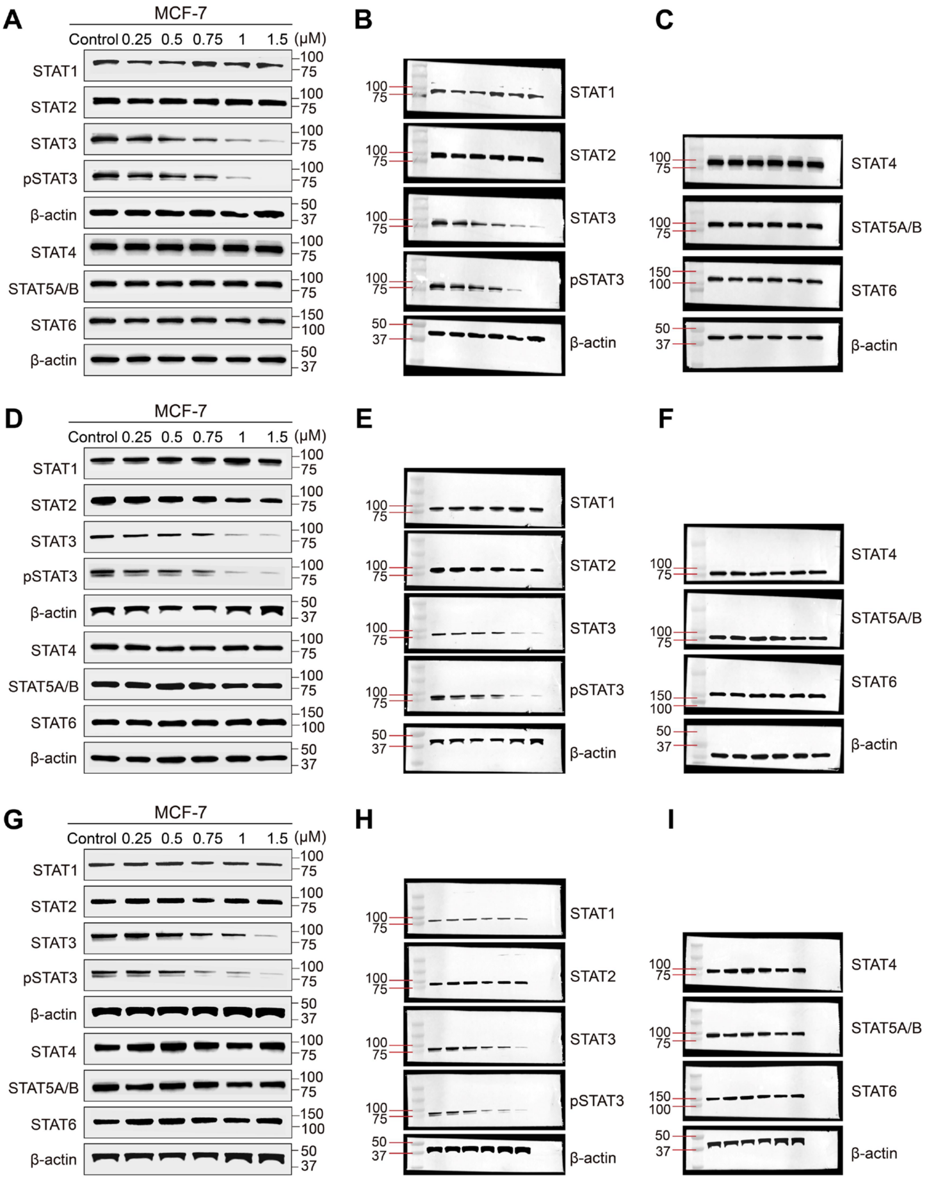

Fig. S16. (A, C, E) Three replicates of experiments showing the change trends of STAT3, pSTAT3 and other STAT family members proteins degradation in MCF-7 cell by treatment with D11-PROTAC. (B, D, F) Corresponding original data graphs to (A, C, E), displaying the merging of markers and protein bands. STAT1, STAT2, STAT3 and pSTAT3 proteins analyzed through WB in this research were run on the same SDS-PAGE gel and transferred to the same PVDF membrane. Following the assessment of pSTAT3 expression levels, the antibodies were stripped using a stripping buffer, re-blocked, and then re-incubated with STAT3 antibodies to detect STAT3 expression levels. The protein expression levels of STAT1 and STAT2 were detected sequentially following this procedure. STAT4, STAT5A/B and STAT6 were also imaged on the same PVDF membrane to detect their protein expression levels in the order of STAT4, STAT5A/B and STAT6. **The data used in Fig. 2N in the main text is Fig. S16A.**

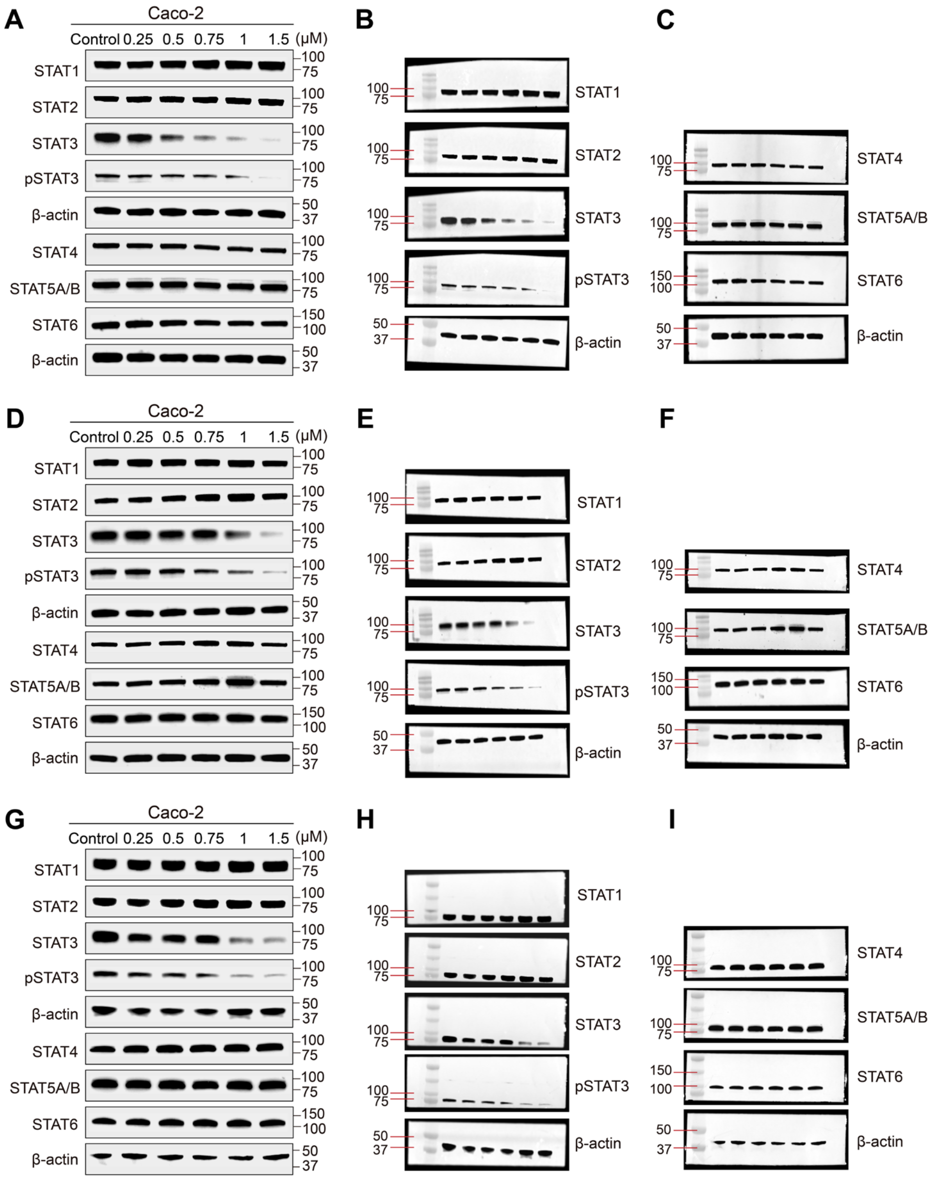

Fig. S17. (A, C, E) Three replicates of experiments showing the change trends of STAT3, pSTAT3 and other STAT family members proteins degradation in Caco-2 cell by treatment with D11-PROTAC. (B, D, F) Corresponding original data graphs to (A, C, E), displaying the merging of markers and protein bands. STAT1, STAT2, STAT3 and pSTAT3 proteins analyzed through WB in this research were run on the same SDS-PAGE gel and transferred to the same PVDF membrane. Following the assessment of pSTAT3 expression levels, the antibodies were stripped using a stripping buffer, re-blocked, and then re-incubated with STAT3 antibodies to detect STAT3 expression levels. The protein expression levels of STAT1 and STAT2 were detected sequentially following this procedure. STAT4, STAT5A/B and STAT6 were also imaged on the same PVDF membrane to detect their protein expression levels in the order of STAT4, STAT5A/B and STAT6.

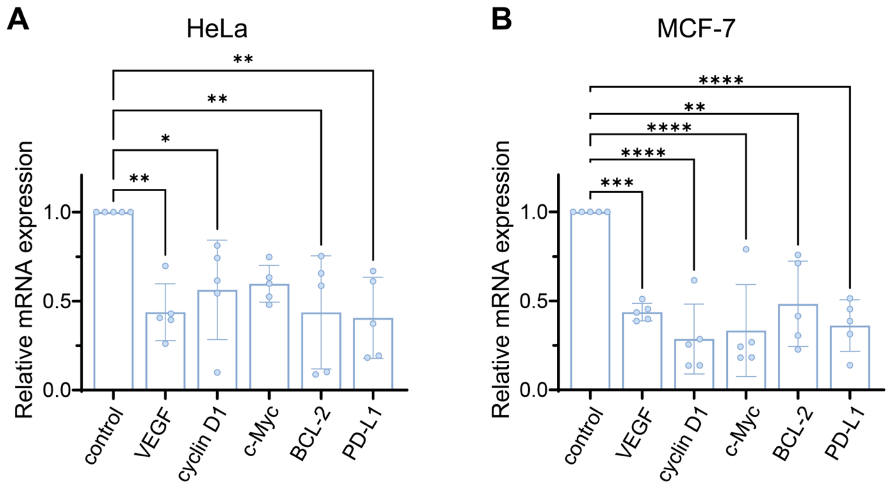

Fig. S18. The crucial downstream STAT3 targets mRNA level in HeLa and MCF-7 cells (β-actin as housekeeping gene). The data are presented as the mean: SD values; $n = 5$. *$P < 0.05$, **$P < 0.01$, ***$P < 0.001$, and ****$P < 0.0001$ vs. the control group.

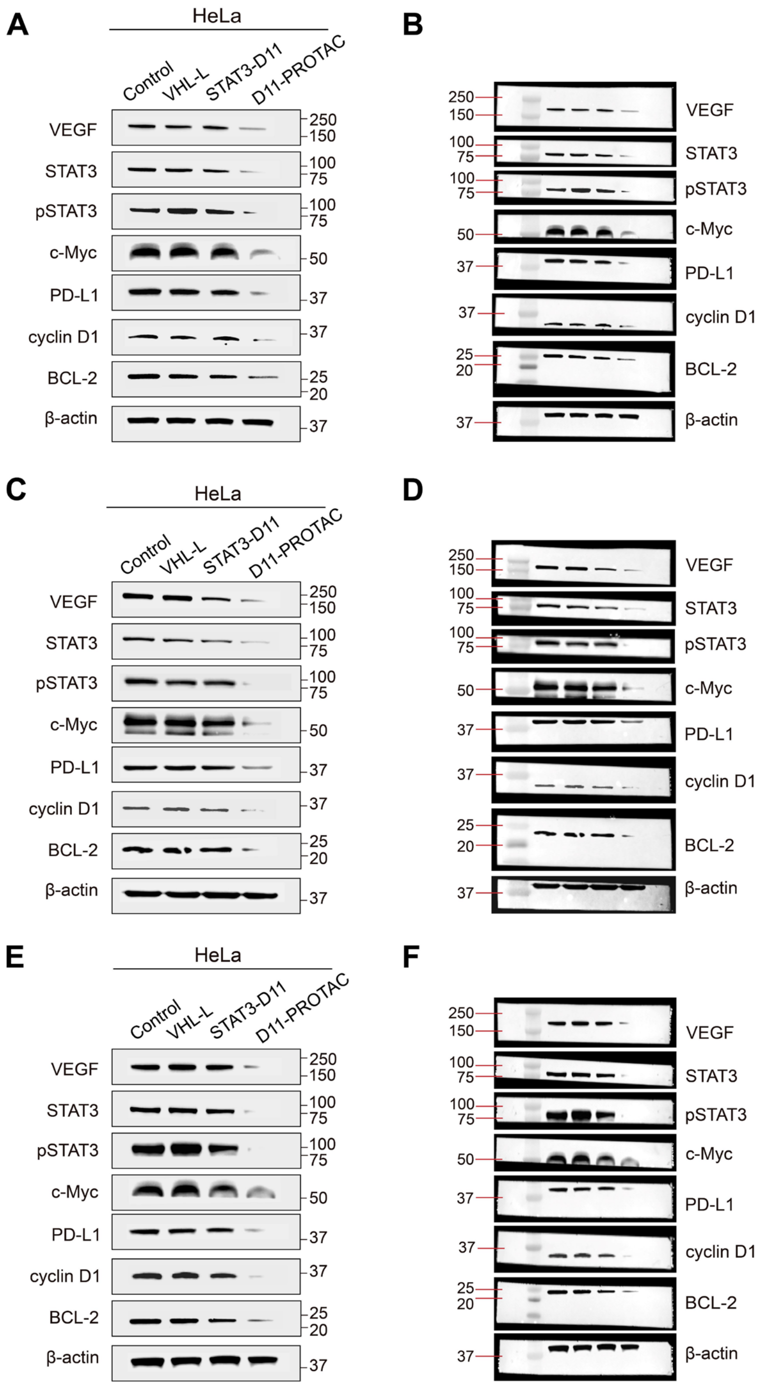

Fig. S19. (A, C, E) Three replicates of experiments showing the change trends of STAT3, pSTAT3 and other crucial downstream STAT3 targets proteins (VEGF, c-Myc, PD-L1, cyclin D1 and BCL-2) degradation in HeLa cell by treatment with D11-PROTAC. (B, D, F) Corresponding original data graphs to (A, C, E), displaying the merging of markers and protein bands. All proteins analyzed through WB in this research were run on the same SDS-PAGE gel and transferred to the same PVDF membrane. Following the assessment of pSTAT3 expression levels, the antibodies were stripped using a stripping buffer, re-blocked, and then re-incubated with STAT3 antibodies to detect STAT3 expression levels. Cyclin D1, PD-L1 and β-actin were also imaged on the same PVDF membrane to detect their protein expression levels in the order of cyclin D1, PD-L1 and β-actin. **The data used in Fig. 3B, 4B and 5B in the main text is Fig. S19A.**

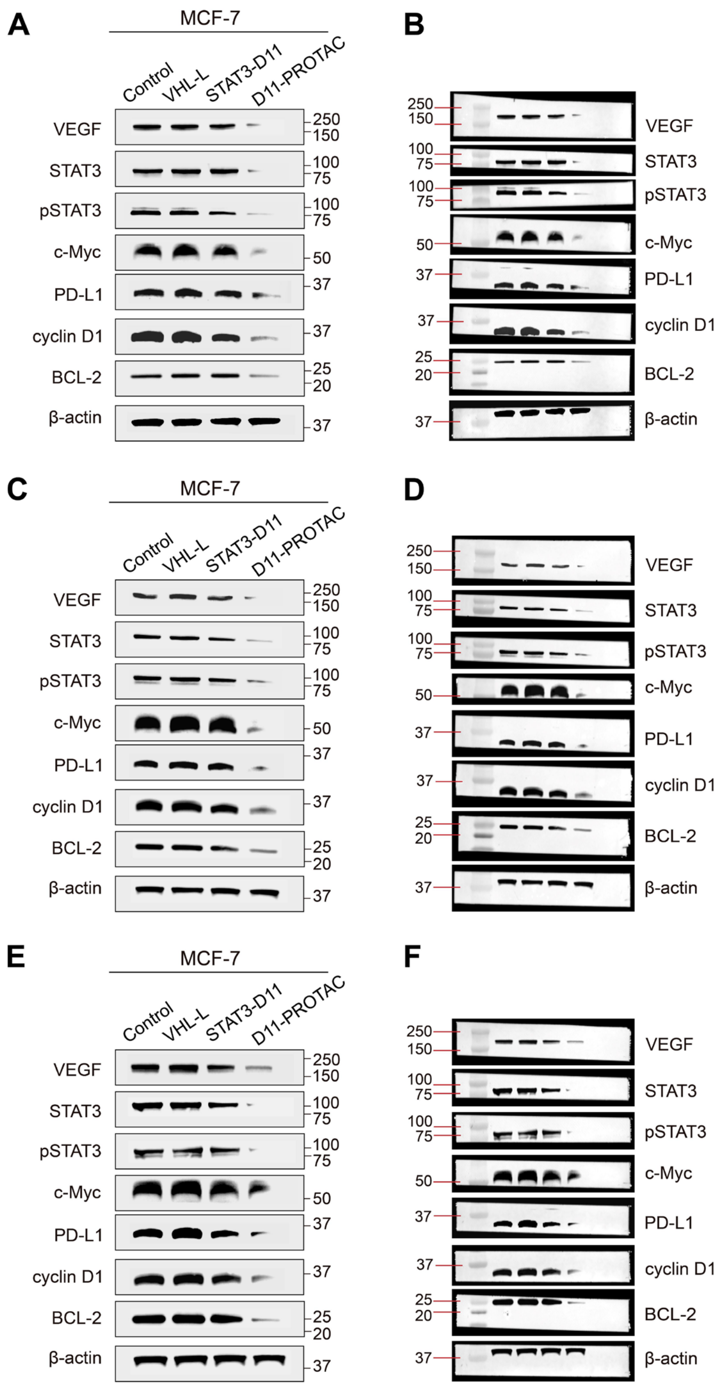

Fig. S20. (A, C, E) Three replicates of experiments showing the change trends of STAT3, pSTAT3 and other crucial downstream STAT3 targets proteins (VEGF, c-Myc, PD-L1, cyclin D1 and BCL-2) degradation in MCF-7 cell by treatment with D11-PROTAC. (B, D, F) Corresponding original data graphs to (A, C, E), displaying the merging of markers and protein bands. All proteins analyzed through WB in this research were run on the same SDS-PAGE gel and transferred to the same PVDF membrane. Following the assessment of pSTAT3 expression levels, the antibodies were stripped using a stripping buffer, re-blocked, and then re-incubated with STAT3 antibodies to detect STAT3 expression levels. Cyclin D1, PD-L1 and β-actin were also imaged on the same PVDF membrane to detect their protein expression levels in the order of cyclin D1, PD-L1 and β-actin. **The data used in Fig. 3D, 4D and 5D in the main text is Fig. S20A.**

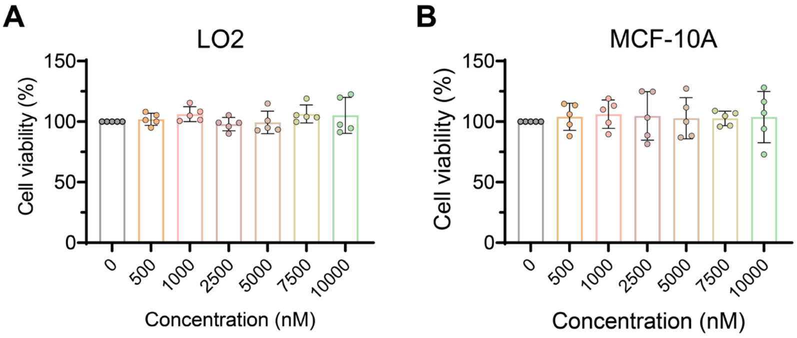

Fig. S21. CCK-8 assay: LO2 and MCF-10A cells viability at different concentrations of D11-PROTAC for 48 h.

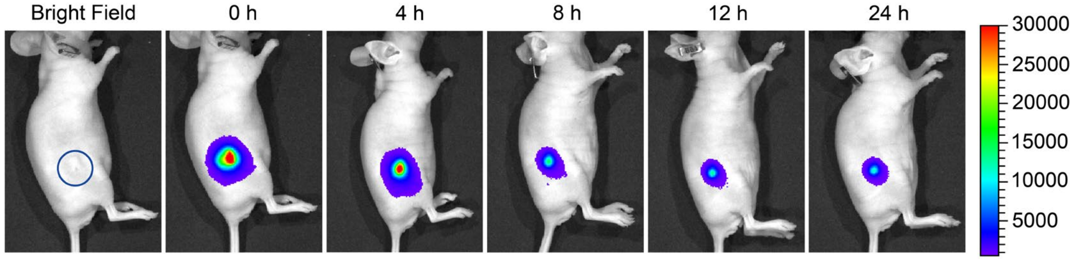

Fig. S22. In vivo fluorescent images of MCF-7 tumor-bearing mouse at 0, 4, 8, 12, and 24 h after intratumoral injection of Cy5-modified D11-PROTAC.

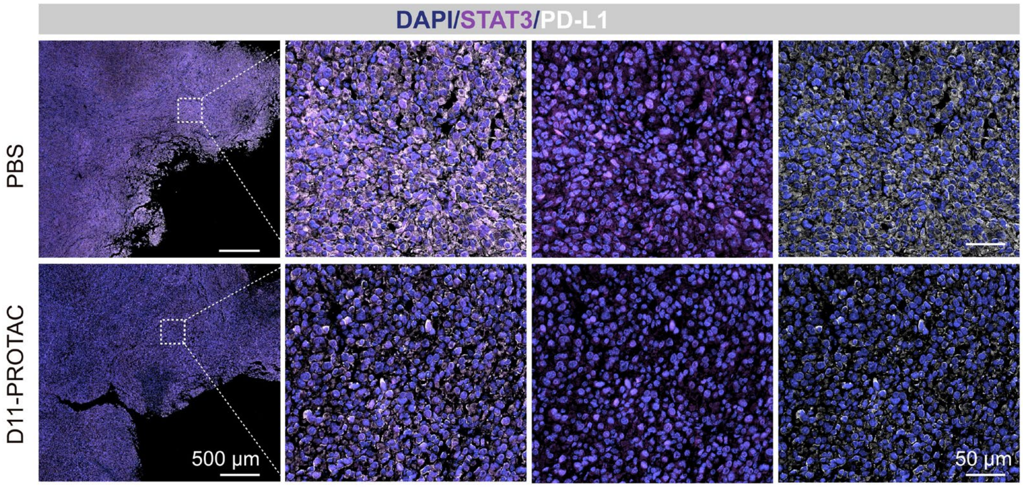

Fig. S23. Immunofluorescence STAT3 and PD-L1 staining images of tumors from different groups.

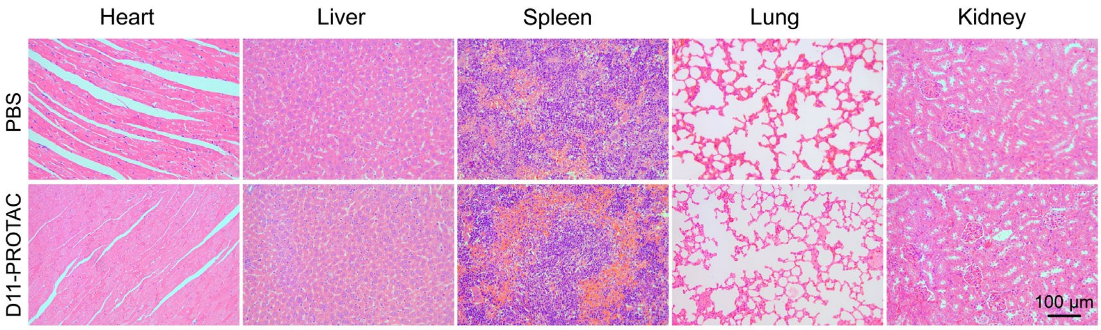

Fig. S24. H&E staining of the major organs (heart, liver, spleen, lung, and kidney) on the 28th day from different groups.
