# Supplementary material for: Decoy-PROTAC for specific degradation of “Undruggable” STAT3 transcription factor
Source: Cell Death Dis. 2025 Mar 21;16(1):197. doi: 10.1038/s41419-025-07535-x (PMC11928565; doi:10.1038/s41419-025-07535-x)
Supplement: Supplementary file 2 — western blots original data [file 41419_2025_7535_MOESM2_ESM.pdf]

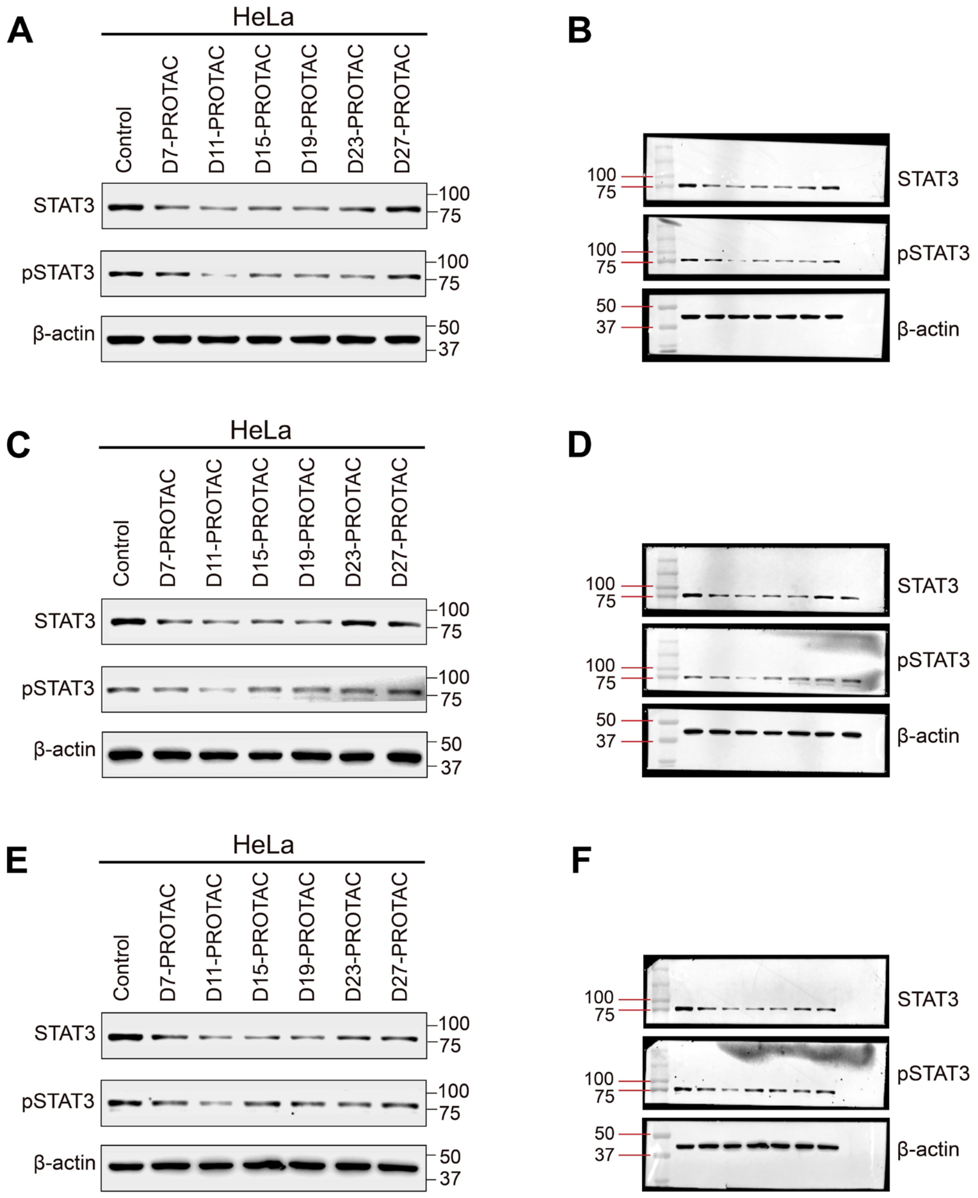

Fig. S4. (A, C, E) Three replicates of experiments showing the change trends of STAT3 and pSTAT3 proteins degradation in HeLa cell by D-PROTACs with different linker lengths (7, 11, 15, 19, 23, 27 bases). (B, D, F) Corresponding original data graphs to (A, C, E), displaying the merging of markers and protein bands. STAT3 and pSTAT3 proteins analyzed through WB in this research were run on the same SDS-PAGE gel and transferred to the same PVDF membrane. Following the assessment of pSTAT3 expression levels, the antibodies were stripped using a stripping buffer, re-blocked, and then re-incubated with STAT3 antibodies to detect STAT3 expression levels. **The data used in Fig. 1B in the main text is Fig. S4A.**

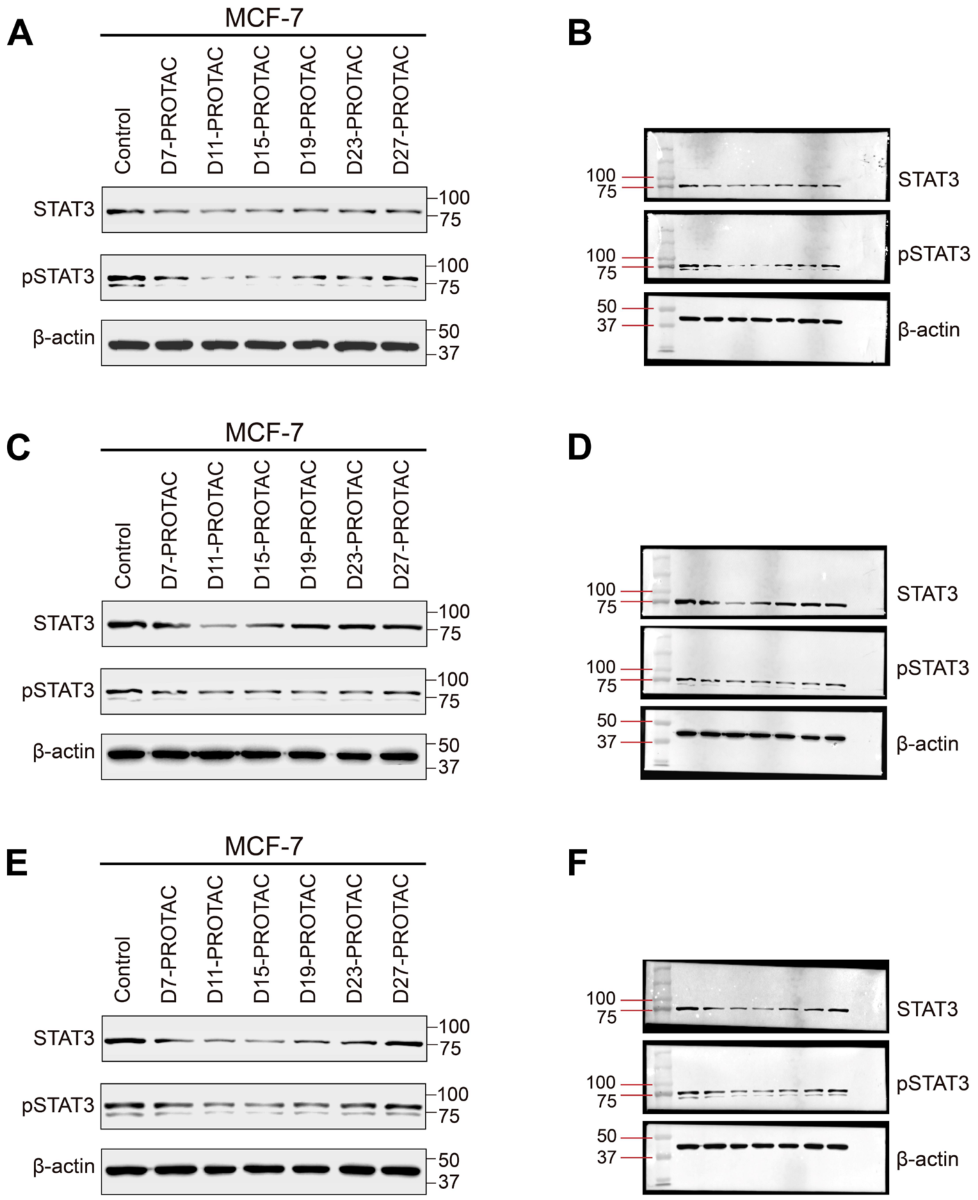

Fig. S5. (A, C, E) Three replicates of experiments showing the change trends of STAT3 and pSTAT3 proteins degradation in MCF-7 cell by D-PROTACs with different linker lengths (7, 11, 15, 19, 23, 27 bases). (B, D, F) Corresponding original data graphs to (A, C, E), displaying the merging of markers and protein bands. STAT3 and pSTAT3 proteins analyzed through WB in this research were run on the same SDS-PAGE gel and transferred to the same PVDF membrane. Following the assessment of pSTAT3 expression levels, the antibodies were stripped using a stripping buffer, re-blocked, and then re-incubated with STAT3 antibodies to detect STAT3 expression levels. **The data used in Fig. 1D in the main text is Fig. S5A.**

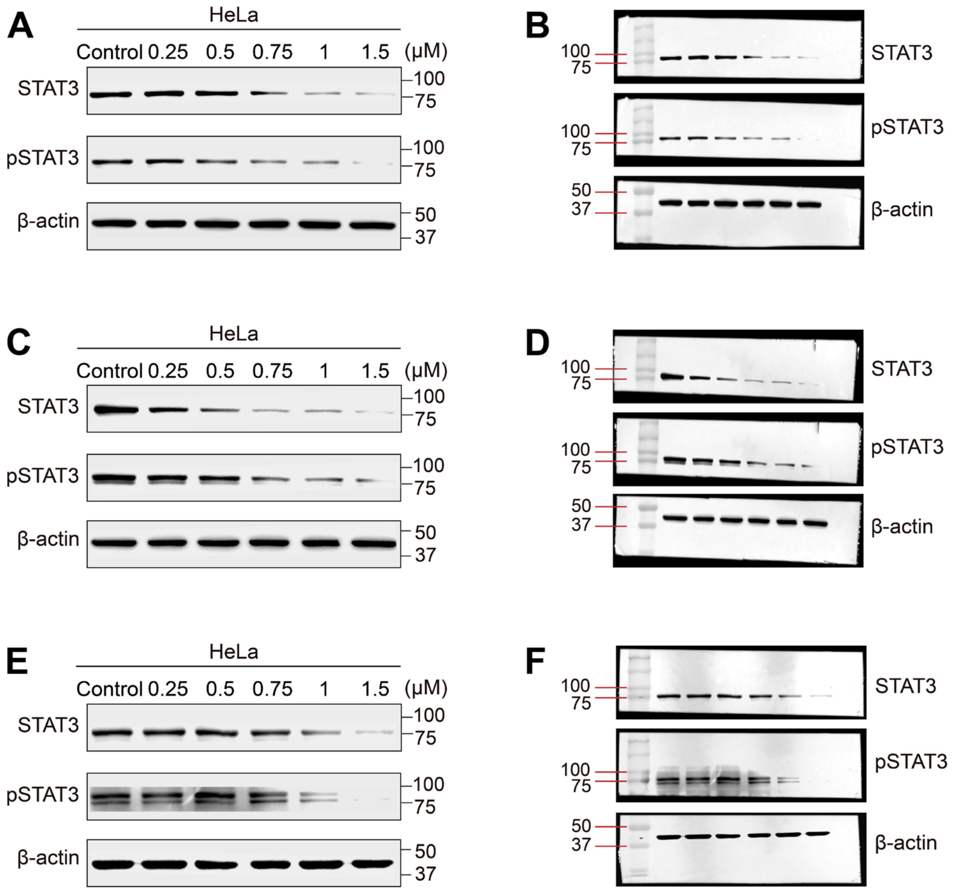

Fig. S6. (A, C, E) Three replicates of experiments showing the change trends of STAT3 and pSTAT3 proteins degradation in HeLa cell by D11-PROTAC with different concentrations (0.25, 0.5, 0.75, 1 and 1.5 μM). (B, D, F) Corresponding original data graphs to (A, C, E), displaying the merging of markers and protein bands. STAT3 and pSTAT3 proteins analyzed through WB in this research were run on the same SDS-PAGE gel and transferred to the same PVDF membrane. Following the assessment of pSTAT3 expression levels, the antibodies were stripped using a stripping buffer, re-blocked, and then re-incubated with STAT3 antibodies to detect STAT3 expression levels. **The data used in Fig. 2A in the main text is Fig. S6A.**

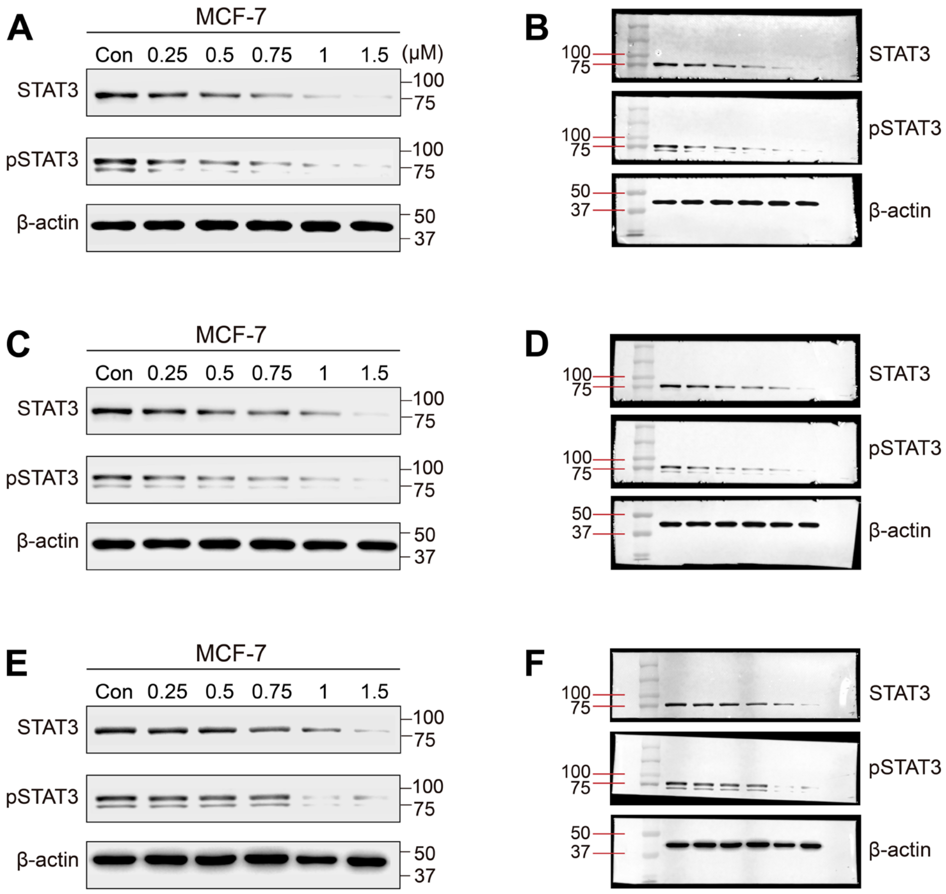

Fig. S7. (A, C, E) Three replicates of experiments showing the change trends of STAT3 and pSTAT3 proteins degradation in MCF-7 cell by D11-PROTAC with different concentrations (0.25, 0.5, 0.75, 1 and 1.5 μM). (B, D, F) Corresponding original data graphs to (A, C, E), displaying the merging of markers and protein bands. STAT3 and pSTAT3 proteins analyzed through WB in this research were run on the same SDS-PAGE gel and transferred to the same PVDF membrane. Following the assessment of pSTAT3 expression levels, the antibodies were stripped using a stripping buffer, re-blocked, and then re-incubated with STAT3 antibodies to detect STAT3 expression levels. **The data used in Fig. 2C in the main text is Fig. S7A.**

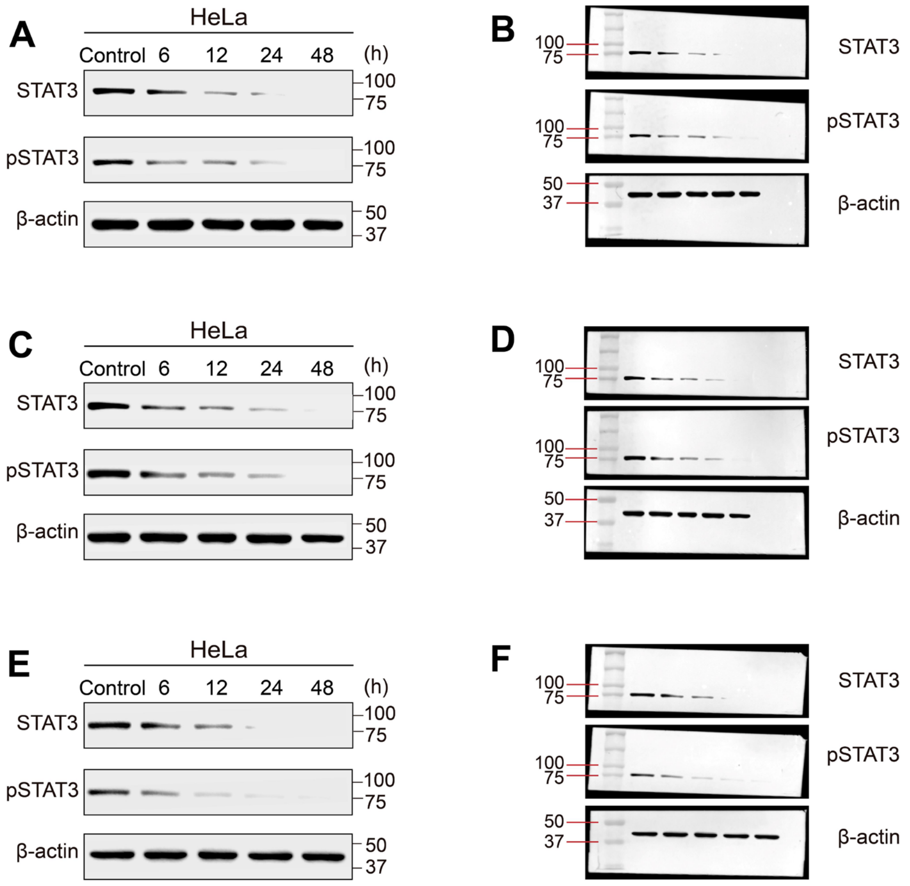

Fig. S8. (A, C, E) Three replicates of experiments showing the change trends of STAT3 and pSTAT3 proteins degradation in HeLa cell by D11-PROTAC with different times (6, 12, 24 and 48 h). (B, D, F) Corresponding original data graphs to (A, C, E), displaying the merging of markers and protein bands. STAT3 and pSTAT3 proteins analyzed through WB in this research were run on the same SDS-PAGE gel and transferred to the same PVDF membrane. Following the assessment of pSTAT3 expression levels, the antibodies were stripped using a stripping buffer, re-blocked, and then re-incubated with STAT3 antibodies to detect STAT3 expression levels. **The data used in Fig. 2E in the main text is Fig. S8A.**

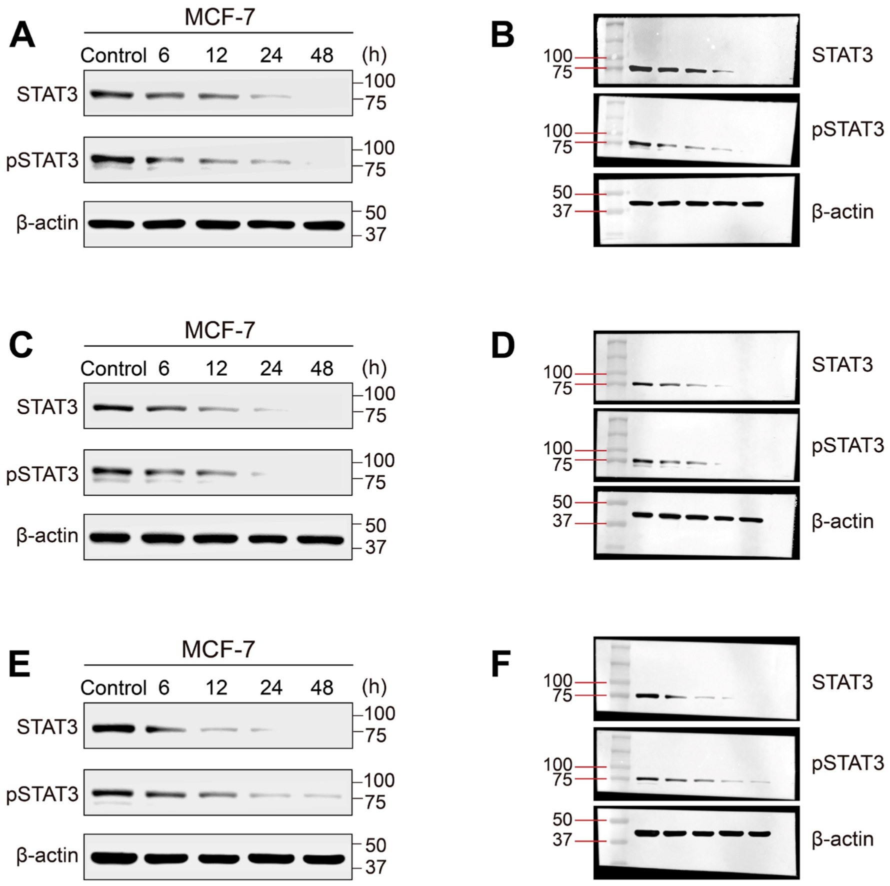

Fig. S9. (A, C, E) Three replicates of experiments showing the change trends of STAT3 and pSTAT3 proteins degradation in MCF-7 cell by D11-PROTAC with different times (6, 12, 24 and 48 h). (B, D, F) Corresponding original data graphs to (A, C, E), displaying the merging of markers and protein bands. STAT3 and pSTAT3 proteins analyzed through WB in this research were run on the same SDS-PAGE gel and transferred to the same PVDF membrane. Following the assessment of pSTAT3 expression levels, the antibodies were stripped using a stripping buffer, re-blocked, and then re-incubated with STAT3 antibodies to detect STAT3 expression levels. **The data used in Fig. 2G in the main text is Fig. S9A.**

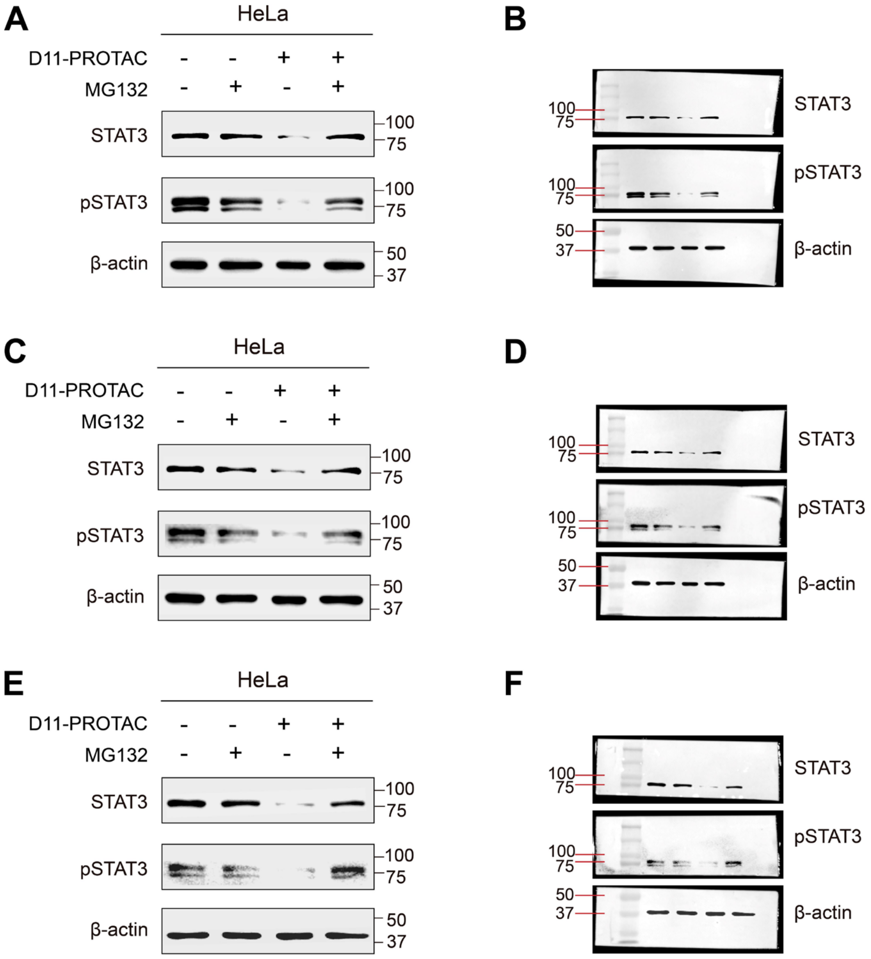

Fig. S10. (A, C, E) Three replicates of experiments showing the change trends of STAT3 and pSTAT3 proteins degradation in HeLa cell by treatment with D11-PROTAC and proteasome inhibitor MG132. (B, D, F) Corresponding original data graphs to (A, C, E), displaying the merging of markers and protein bands. STAT3 and pSTAT3 proteins analyzed through WB in this research were run on the same SDS-PAGE gel and transferred to the same PVDF membrane. Following the assessment of pSTAT3 expression levels, the antibodies were stripped using a stripping buffer, re-blocked, and then re-incubated with STAT3 antibodies to detect STAT3 expression levels. **The data used in Fig. 2I in the main text is Fig. S10A.**

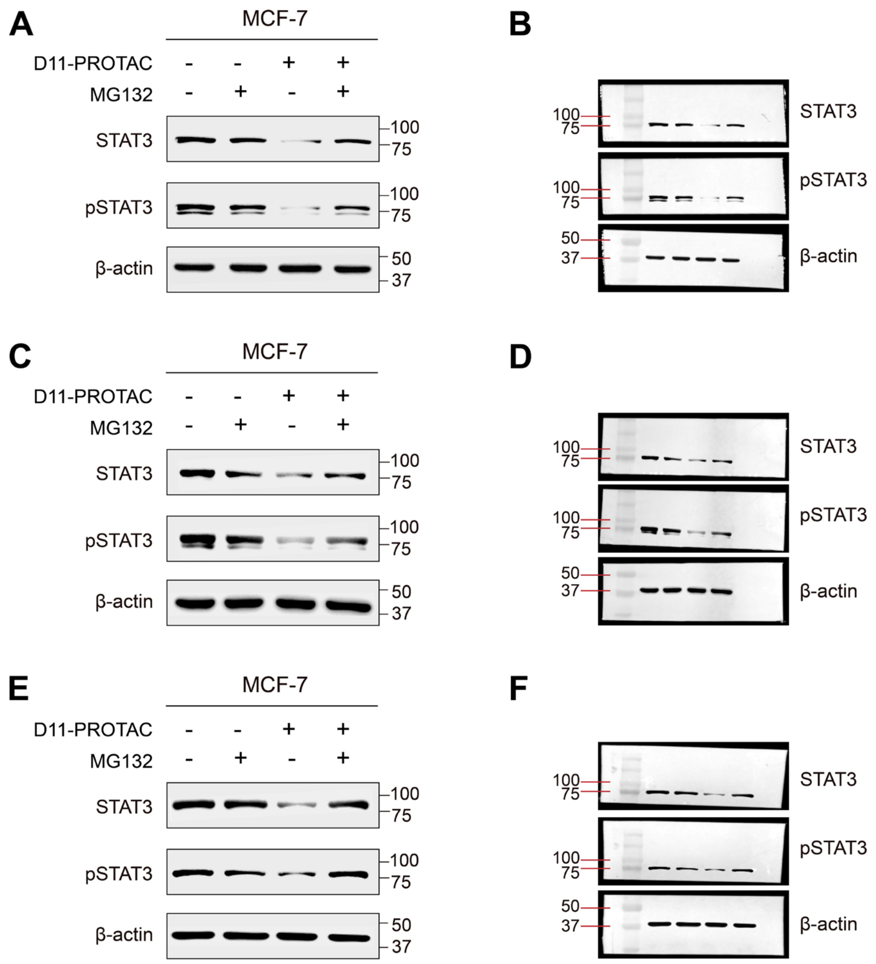

Fig. S11. (A, C, E) Three replicates of experiments showing the change trends of STAT3 and pSTAT3 proteins degradation in MCF-7 cell by treatment with D11-PROTAC and proteasome inhibitor MG132. (B, D, F) Corresponding original data graphs to (A, C, E), displaying the merging of markers and protein bands. STAT3 and pSTAT3 proteins analyzed through WB in this research were run on the same SDS-PAGE gel and transferred to the same PVDF membrane. Following the assessment of pSTAT3 expression levels, the antibodies were stripped using a stripping buffer, re-blocked, and then re-incubated with STAT3 antibodies to detect STAT3 expression levels. **The data used in Fig. 2K in the main text is Fig. S11A.**

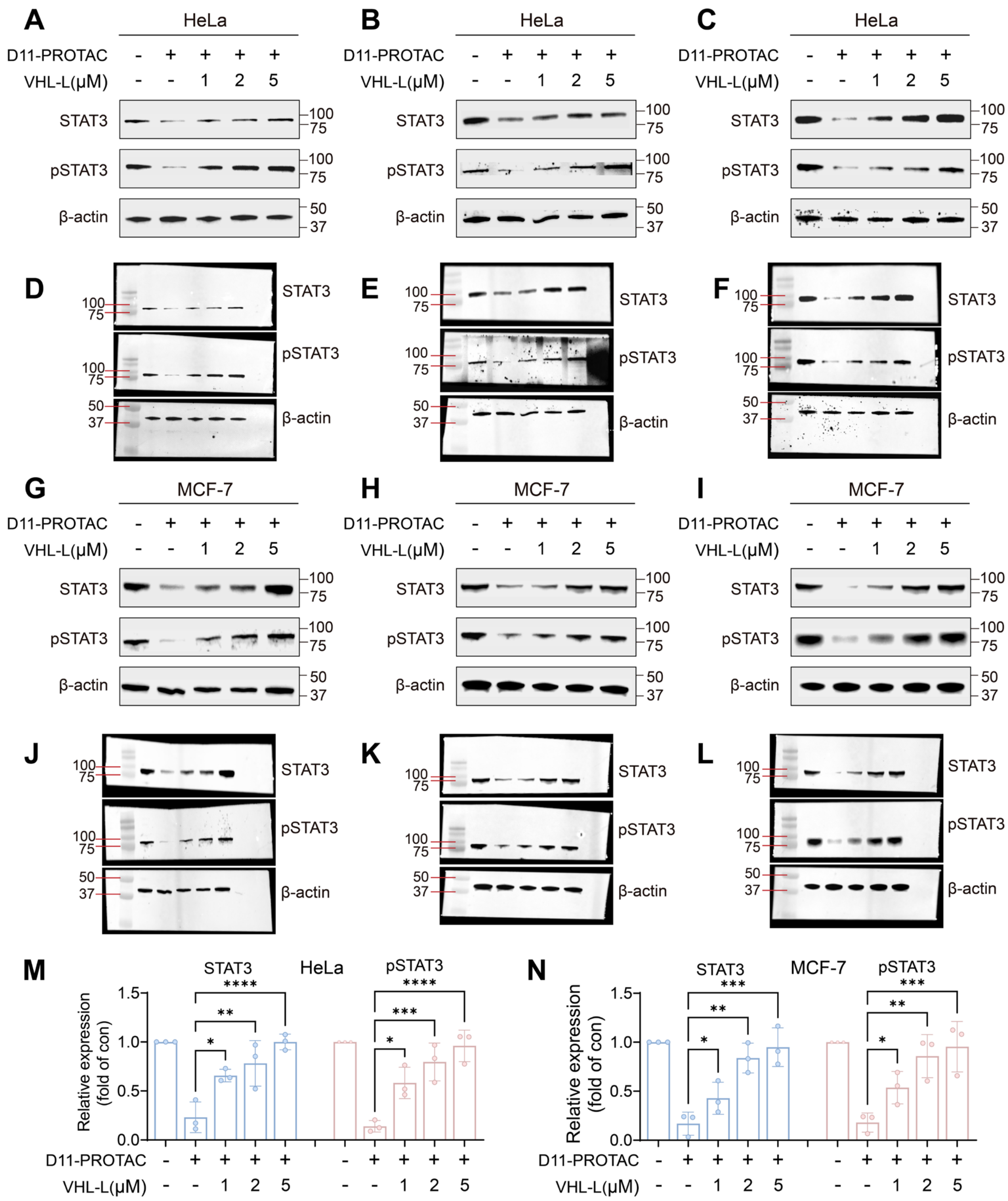

Fig. S12. (A-C, G-I) Three replicates of experiments showing the change trends of STAT3 and pSTAT3 proteins degradation in HeLa and MCF-7 cell by treatment with D11-PROTAC and VHL ligand (VHL-L). (D-F, J-L) Corresponding original data graphs to (A-C, G-I), displaying the merging of markers and protein bands. STAT3 and pSTAT3 proteins analyzed through WB in this research were run on the same SDS-PAGE gel and transferred to the same PVDF membrane. Following the assessment of pSTAT3 expression levels, the antibodies were stripped using a stripping buffer, re-blocked, and then re-incubated with STAT3 antibodies to detect STAT3 expression levels. The data are presented as the mean: SD values; $n = 3$. $^*P < 0.05$, $^{**}P < 0.01$, $^{***}P < 0.001$, and $^{****}P < 0.0001$ vs. the control group.

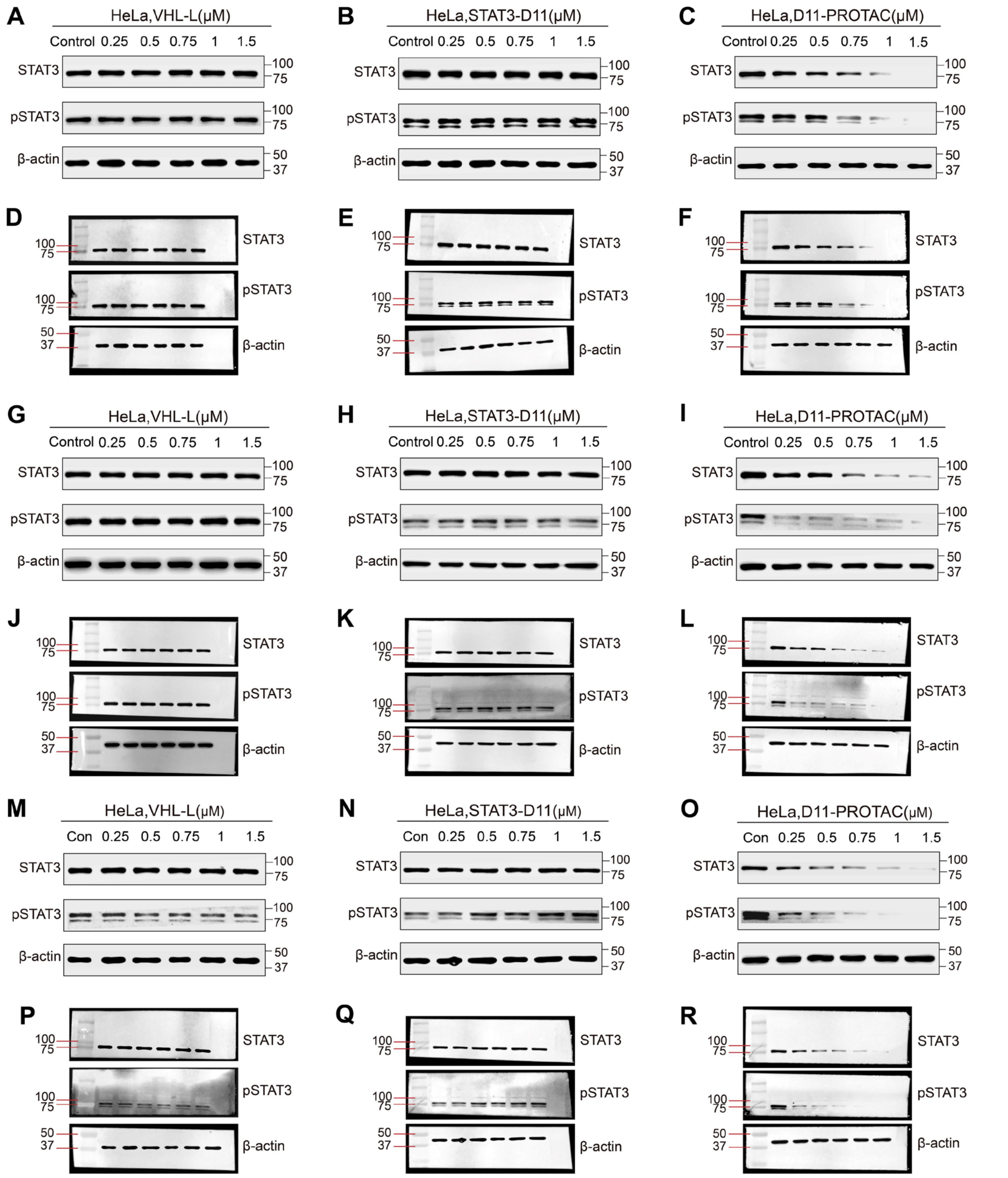

Fig. S13. Incubation of VHL-L, STAT3-D11, and D11-PROTAC in HeLa cell revealed that only D11-PROTAC was capable of degrading STAT3 protein. (A-C, G-I, M-O) The three replicates of experiments showing the change trends of STAT3 and pSTAT3 proteins degradation in HeLa cell by incubation of VHL-L, STAT3-D11, and D11-PROTAC at a concentration of 1.5 μM for 24 h and analyzed by WB. (D-F, J-L, P-R) Corresponding original data graphs to (A-C, G-I, M-O), displaying the merging of markers and protein bands. STAT3 and pSTAT3 proteins analyzed through WB in this research were run on the same SDS-PAGE gel and transferred to the same PVDF membrane. Following the assessment of pSTAT3 expression levels, the antibodies were stripped using a stripping buffer, re-blocked, and then re-incubated with STAT3 antibodies to detect STAT3 expression levels.

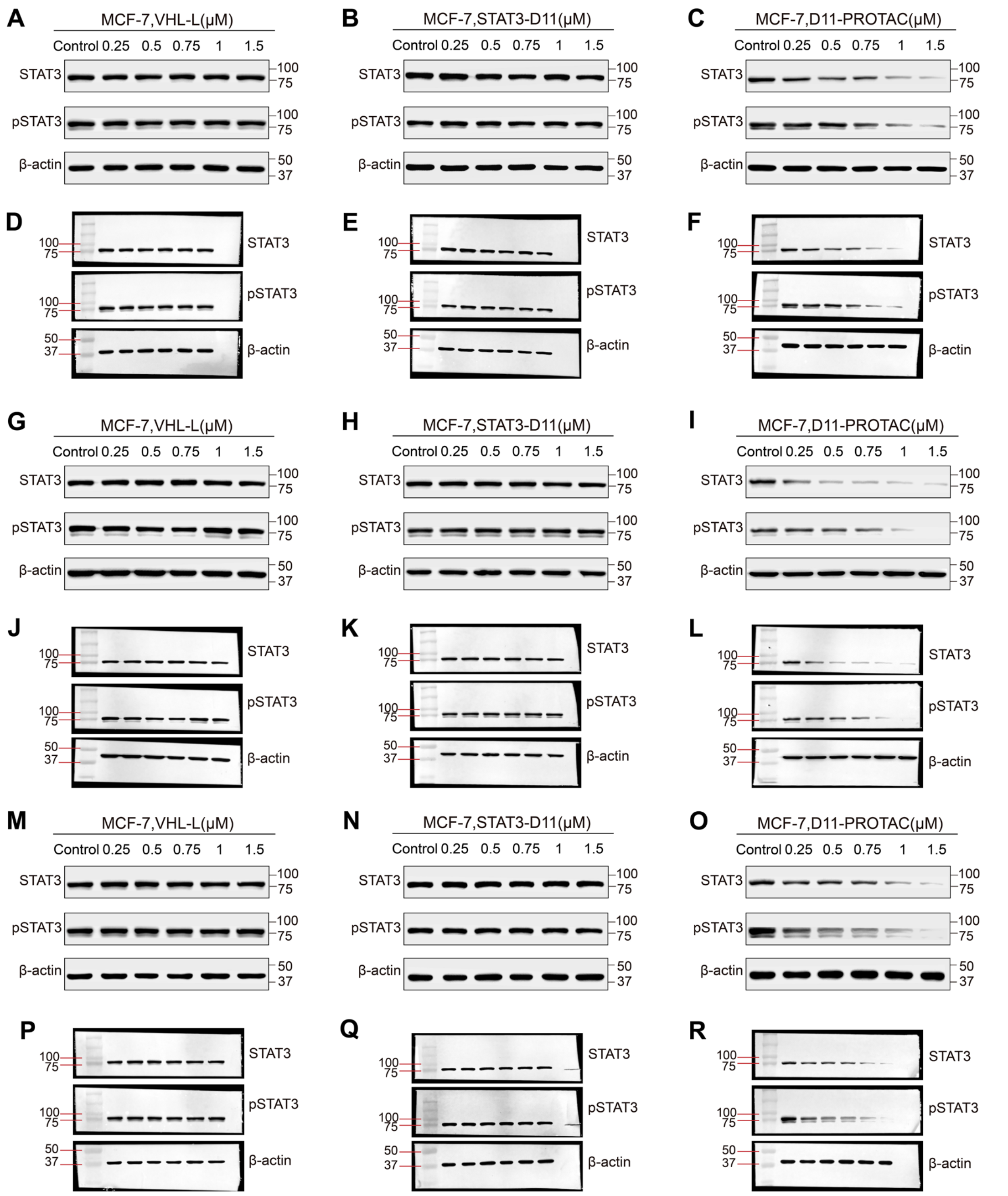

Fig. S14. Incubation of VHL-L, STAT3-D11, and D11-PROTAC in MCF-7 cell revealed that only D11-PROTAC was capable of degrading STAT3 protein. (A-C, G-I, M-O) The three replicates of experiments showing the change trends of STAT3 and pSTAT3 proteins degradation in MCF-7 cell by incubation of VHL-L, STAT3-D11, and D11-PROTAC at a concentration of 1.5 μM for 24 h and analyzed by WB. (D-F, J-L, P-R) Corresponding original data graphs to (A-C, G-I, M-O), displaying the merging of markers and protein bands. STAT3 and pSTAT3 proteins analyzed through WB in this research were run on the same SDS-PAGE gel and transferred to the same PVDF membrane. Following the assessment of pSTAT3 expression levels, the antibodies were stripped using a stripping buffer, re-blocked, and then re-incubated with STAT3 antibodies to detect STAT3 expression levels.

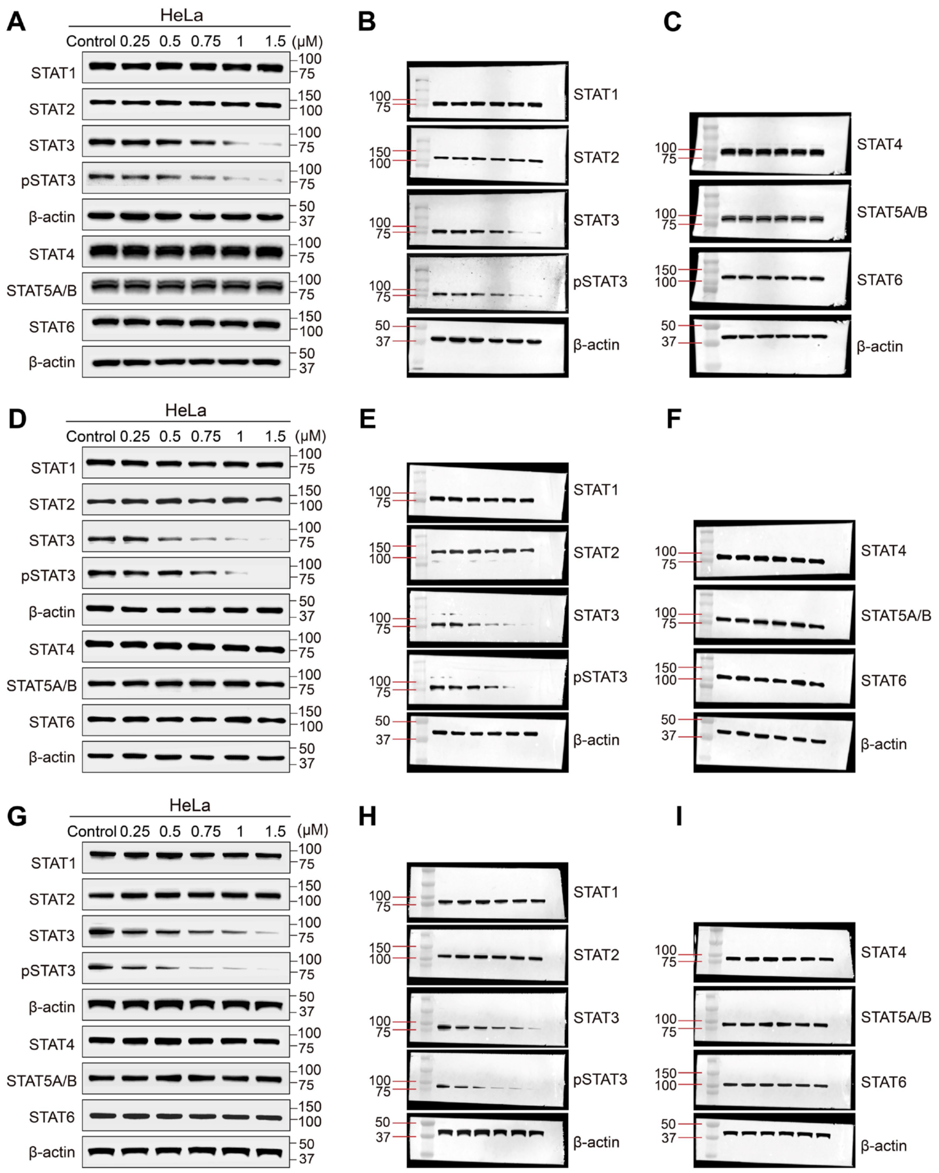

Fig. S15. (A, C, E) Three replicates of experiments showing the change trends of STAT3, pSTAT3 and other STAT family members proteins degradation in HeLa cell by treatment with D11-PROTAC. (B, D, F) Corresponding original data graphs to (A, C, E), displaying the merging of markers and protein bands. STAT1, STAT2, STAT3 and pSTAT3 proteins analyzed through WB in this research were run on the same SDS-PAGE gel and transferred to the same PVDF membrane. Following the assessment of pSTAT3 expression levels, the antibodies were stripped using a stripping buffer, re-blocked, and then re-incubated with STAT3 antibodies to detect STAT3 expression levels. The protein expression levels of STAT1 and STAT2 were detected sequentially following this procedure. STAT4, STAT5A/B and STAT6 were also imaged on the same PVDF membrane to detect their protein expression levels in the order of STAT4, STAT5A/B and STAT6. **The data used in Fig. 2M in the main text is Fig. S15A.**

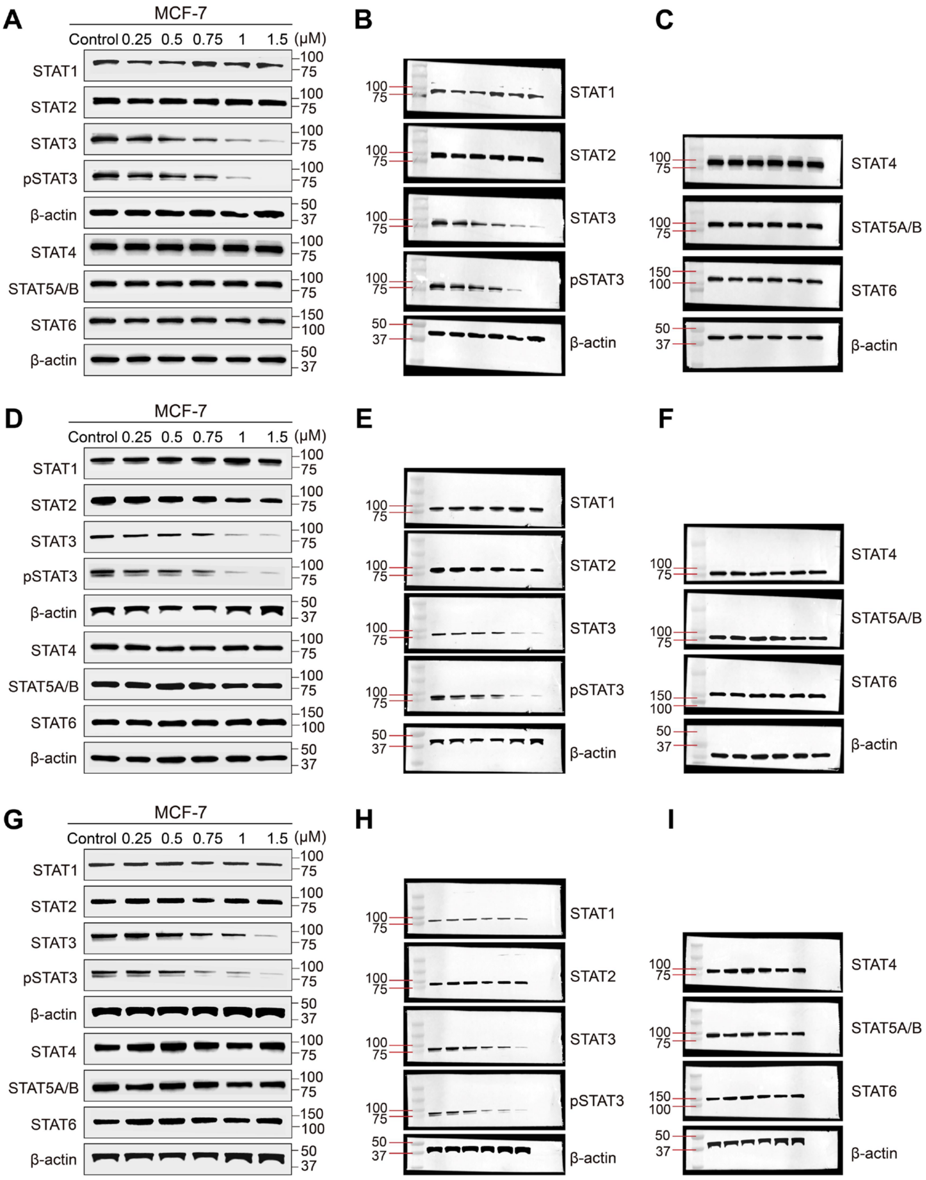

Fig. S16. (A, C, E) Three replicates of experiments showing the change trends of STAT3, pSTAT3 and other STAT family members proteins degradation in MCF-7 cell by treatment with D11-PROTAC. (B, D, F) Corresponding original data graphs to (A, C, E), displaying the merging of markers and protein bands. STAT1, STAT2, STAT3 and pSTAT3 proteins analyzed through WB in this research were run on the same SDS-PAGE gel and transferred to the same PVDF membrane. Following the assessment of pSTAT3 expression levels, the antibodies were stripped using a stripping buffer, re-blocked, and then re-incubated with STAT3 antibodies to detect STAT3 expression levels. The protein expression levels of STAT1 and STAT2 were detected sequentially following this procedure. STAT4, STAT5A/B and STAT6 were also imaged on the same PVDF membrane to detect their protein expression levels in the order of STAT4, STAT5A/B and STAT6. **The data used in Fig. 2N in the main text is Fig. S16A.**

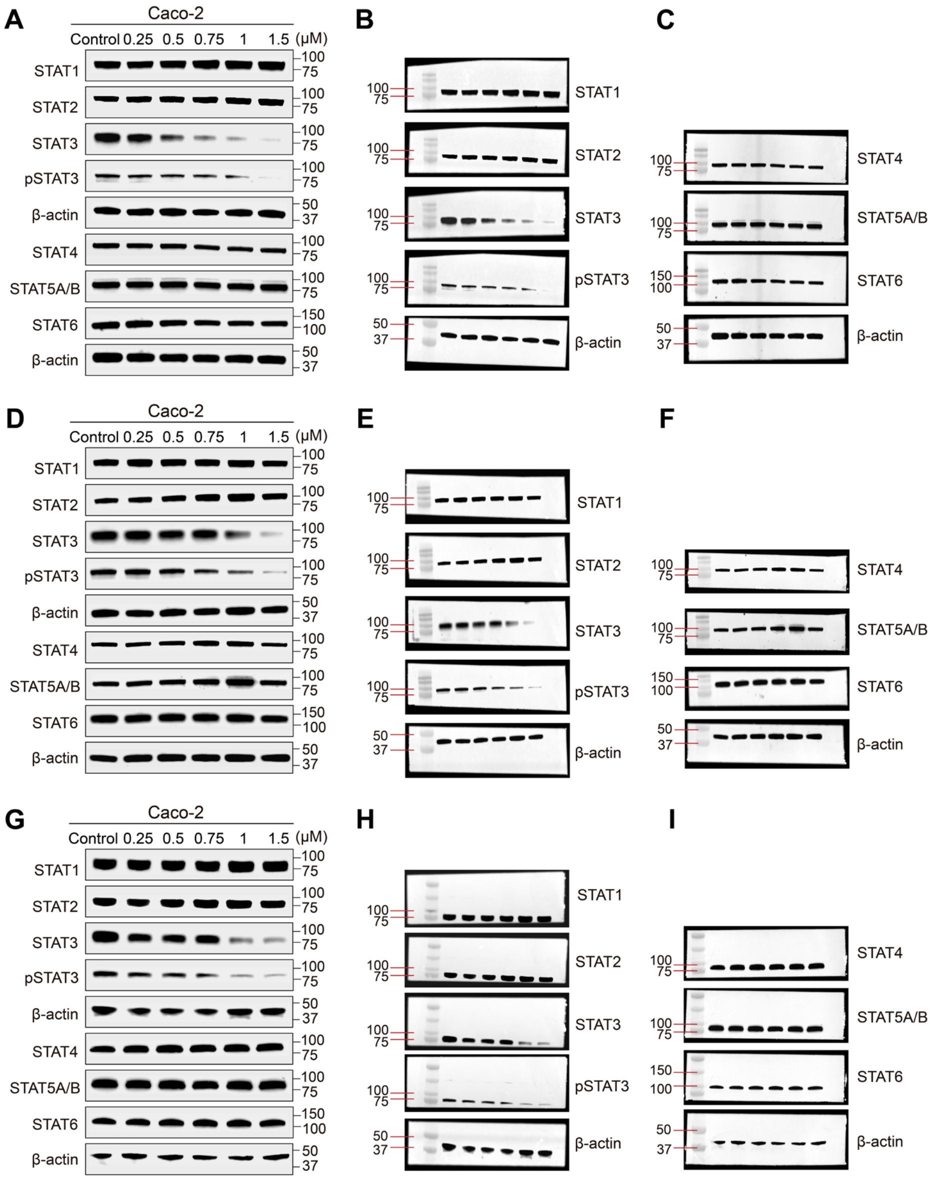

Fig. S17. (A, C, E) Three replicates of experiments showing the change trends of STAT3, pSTAT3 and other STAT family members proteins degradation in Caco-2 cell by treatment with D11-PROTAC. (B, D, F) Corresponding original data graphs to (A, C, E), displaying the merging of markers and protein bands. STAT1, STAT2, STAT3 and pSTAT3 proteins analyzed through WB in this research were run on the same SDS-PAGE gel and transferred to the same PVDF membrane. Following the assessment of pSTAT3 expression levels, the antibodies were stripped using a stripping buffer, re-blocked, and then re-incubated with STAT3 antibodies to detect STAT3 expression levels. The protein expression levels of STAT1 and STAT2 were detected sequentially following this procedure. STAT4, STAT5A/B and STAT6 were also imaged on the same PVDF membrane to detect their protein expression levels in the order of STAT4, STAT5A/B and STAT6.

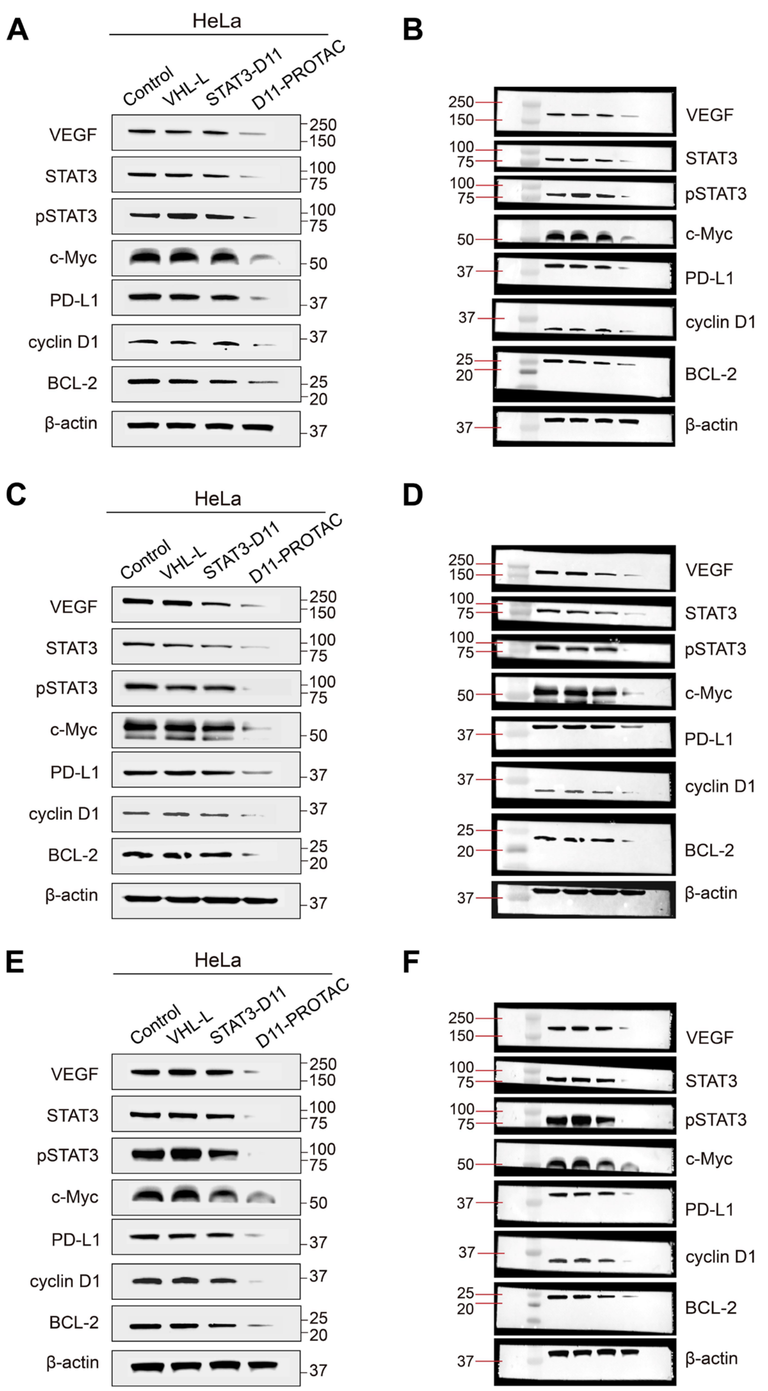

Fig. S19. (A, C, E) Three replicates of experiments showing the change trends of STAT3, pSTAT3 and other crucial downstream STAT3 targets proteins (VEGF, c-Myc, PD-L1, cyclin D1 and BCL-2) degradation in HeLa cell by treatment with D11-PROTAC. (B, D, F) Corresponding original data graphs to (A, C, E), displaying the merging of markers and protein bands. All proteins analyzed through WB in this research were run on the same SDS-PAGE gel and transferred to the same PVDF membrane. Following the assessment of pSTAT3 expression levels, the antibodies were stripped using a stripping buffer, re-blocked, and then re-incubated with STAT3 antibodies to detect STAT3 expression levels. Cyclin D1, PD-L1 and β-actin were also imaged on the same PVDF membrane to detect their protein expression levels in the order of cyclin D1, PD-L1 and β-actin. **The data used in Fig. 3B, 4B and 5B in the main text is Fig. S19A.**

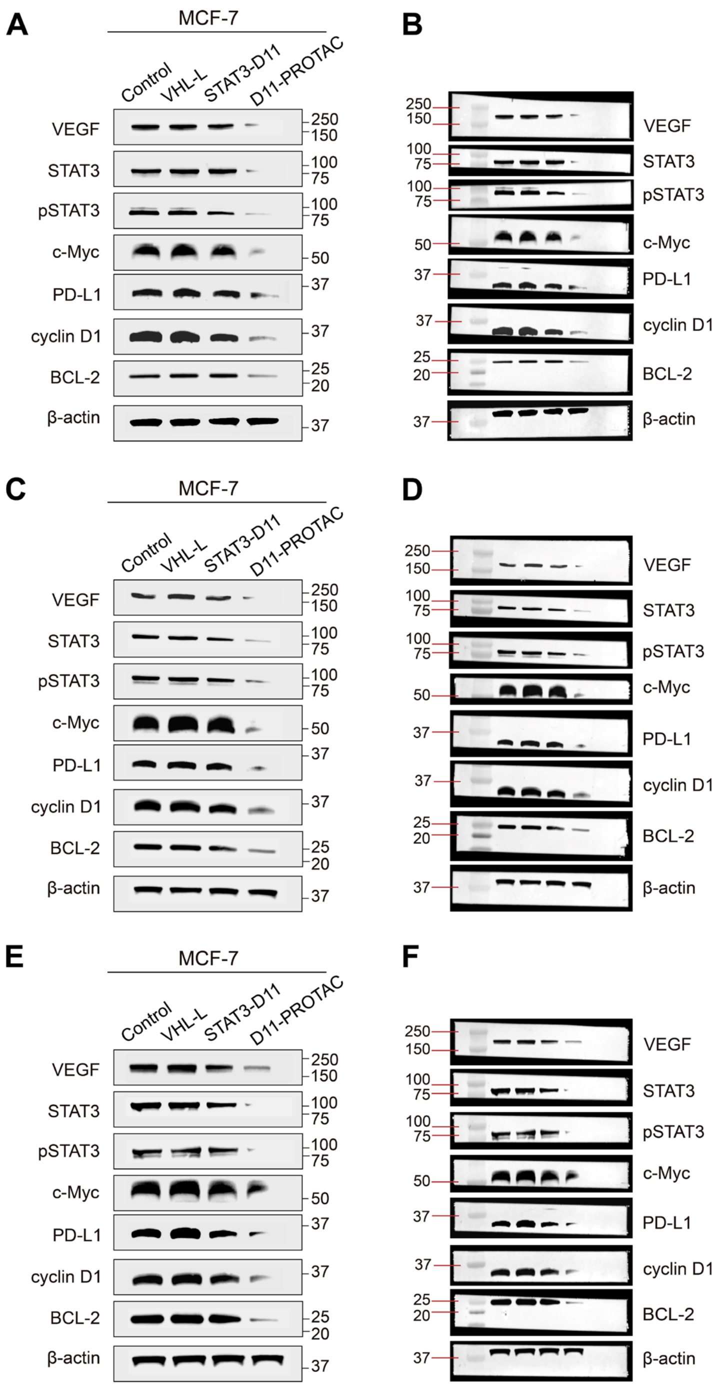

Fig. S20. (A, C, E) Three replicates of experiments showing the change trends of STAT3, pSTAT3 and other crucial downstream STAT3 targets proteins (VEGF, c-Myc, PD-L1, cyclin D1 and BCL-2) degradation in MCF-7 cell by treatment with D11-PROTAC. (B, D, F) Corresponding original data graphs to (A, C, E), displaying the merging of markers and protein bands. All proteins analyzed through WB in this research were run on the same SDS-PAGE gel and transferred to the same PVDF membrane. Following the assessment of pSTAT3 expression levels, the antibodies were stripped using a stripping buffer, re-blocked, and then re-incubated with STAT3 antibodies to detect STAT3 expression levels. Cyclin D1, PD-L1 and β-actin were also imaged on the same PVDF membrane to detect their protein expression levels in the order of cyclin D1, PD-L1 and β-actin. **The data used in Fig. 3D, 4D and 5D in the main text is Fig. S20A.**
